# Rh(II)-mediated domino [4 + 1]-annulation of α-cyanothioacetamides using diazoesters: A new entry for the synthesis of multisubstituted thiophenes

**DOI:** 10.3762/bjoc.13.253

**Published:** 2017-11-30

**Authors:** Jury J Medvedev, Ilya V Efimov, Yuri M Shafran, Vitaliy V Suslonov, Vasiliy A Bakulev, Valerij A Nikolaev

**Affiliations:** 1 Department of Organic Chemistry, St-Petersburg State University, 26 University pr., 198504, Saint-Petersburg, Russia; 2 Institute of Chemistry and Technology, Ural Federal University, 19 Mira Str., 620002, Ekaterinburg, Russia; 3 Center for X-ray Diffraction Studies, St-Petersburg State University, 26 University pr., 198504, Saint-Petersburg, Russia

**Keywords:** [4 + 1]-annulation, catalysis, diazo compounds, domino reactions, thiophenes

## Abstract

A new approach towards the synthesis of multisubstituted thiophenes is elaborated based on Rh(II)-catalyzed domino reactions of acyclic diazoesters with α-cyanothioacetamides. It provides a way for the preparation of 5-amino-3-(alkoxycarbonylamino)thiophene-2-carboxylates, 2-(5-amino-2-methoxycarbonylthiophene-3-yl)aminomalonates and (2-cyano-5-aminothiophene-3-yl)carbamates with the preparative yields of up to 67%. It was also shown that α-cyanothioacetamides easily interact with dirhodium carboxylates to give rather stable 2:1 complexes, resulting in an evident decrease in the efficiency of the catalytic process at moderate temperatures (20–30 °C).

## Introduction

**Scheme 1:**
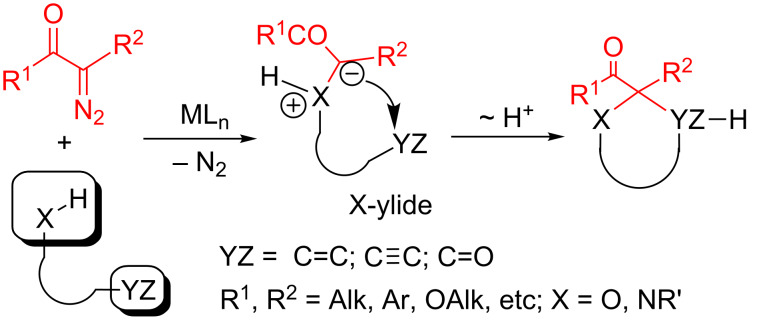
General scheme for intramolecular heterocylization of intermediate X-ylides.

In recent years the diversified reactivity of metal carbenes, catalytically generated from diazocarbonyl compounds, has found wide application in organic synthesis [1–16]. A particular interest was attracted recently to domino reactions of diazo compounds with intermediate formation of ylides [7–21]. Thus, it was for example shown that ammonium or oxonium ylides generated in the course of intermolecular processes can be easily trapped by ketones, imines, α,β-unsaturated carbonyl compounds, activated multiple bonds, or other nucleophiles to furnish heterocyclic cores [22–32]. Similar intramolecular transformations of intermediate ylides with several nucleophilic reaction centers in the initial substrate, are also possible. The known examples of such reactions are for instance syntheses of multisubstituted indolines [23–25], pyrrolidines [26–29], dihydropyrroles [29], tetrahydrofurans [27–28], and 2,5-dihydrofurans [31–32], which proceed as intramolecular interaction of generated ammonium or oxonium ylides with carbonyl groups [23,27–28], C=C double [24–26], or C≡C triple [29–32] bonds in the structure of the initial molecule (Scheme 1).

Intermolecular reaction of metal carbenes with thioamides usually generates thiocarbonyl ylides which leads for example to enaminones [14,33–35] or, in the reaction with *N*-phenyl maleimide, gives rise to formation of S-containing heterocycles by 1,3-dipolar cycloaddition [35]. However, to the best of our knowledge, there are no literature data on analogous intramolecular reactions of C=S ylides involving thiocarbonyl and any other nucleophilic group within the same molecule.

The main objective of our current research was to study Rh(II)-catalyzed reactions of diazocarbonyl compounds with α-cyanothioacetamides, bearing both thioamide and cyano groups in their structure. Based on the known literature findings [36–38] one might expect that a catalytic reaction of diazocarbonyl compounds with α-cyanothioacetamides would first of all affect the electron-rich sulfur atom of the C=S group, leading to the generation of intermediate thiocarbonyl ylides, which would further react intramolecularly with the cyano group to produce a heterocyclic structure. One cannot exclude an alternative route when the carbenoid interacts with the cyano group to yield an oxazole heterocycle [39–45]. Herein we present the first detailed results of this study.

## Results and Discussion

**Figure 1:**
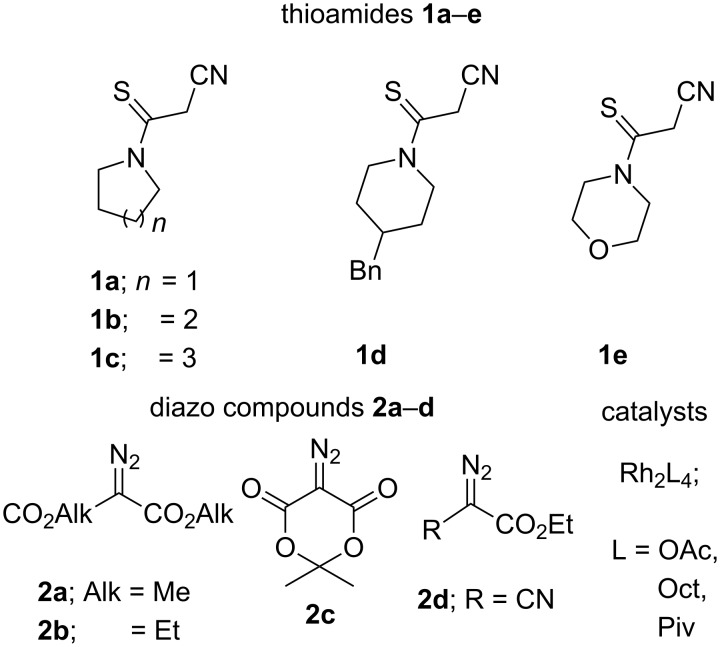
Thioamides **1a**–**e**, diazoesters **2a**–**d** and Rh(II)-catalysts used in the project.

To determine the scope and limitations of these reactions, several thioamides **1a**–**e** of cyanoacetic acid (differing in the structure of substituents in the amino fragment) and diazoesters of three types: acyclic diazomalonates **2a**,**b**, their cyclic analogue, 5-diazo-2,2-dimethyl-1,3-dioxane-4,6-dione (diazo Meldrum’s acid, **2c**), as well as α-cyanodiazoacetic ester **2d** were used in the study (Figure 1).

Dirhodium carboxylates [Rh_2_(OAc)_4_, Rh_2_(Oct)_4_ and Rh_2_(Piv)_4_] which were found to be the most effective catalysts in reactions of diazo carbonyl compounds with different substrates [46–47], were employed in this research.

At first, we studied reactions of thioamides **1** with diazomalonates **2a**,**b**, which usually display high reactivity in Rh-catalyzed transformations [26,48–51] (Table 1). Traditionally, similar catalytic reactions of diazomalonates occur under relatively mild conditions [26]. However, our initial attempts to carry out the processes in CH_2_Cl_2_ at room temperature or on heating with dirhodium tetraacetate or the even more active dirhodium tetraoctanoate, did not lead to a notable decomposition of diazoesters **2a**,**b**. At the same time it was found that at elevated temperatures, for example on refluxing the diazoester **2a** with thioacetamide **1a** in the presence of Rh_2_(OAc)_4_ (2 mol %) in benzene solution during 5.5 h, the complete decomposition of **2a** took place to produce 2,4-diaminothiophenes **3a** and **4a** in 51 and 35% yields, respectively (Table 1, entry 1). The products were separated and individually isolated by means of preparative chromatography on silica gel.

**Table 1:** Rh(II)-Catalyzed reactions of diazomalonates **2a,b** with α-cyanothioacetamides **1a**–**e**.

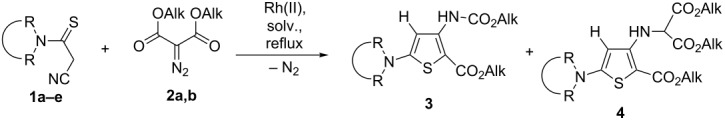						

entry	reactants	catalyst	solvent, reaction time	yield, %^a^		

				**3**	**4**	total (**3** + **4**)
				
1^b^	**1a**; **2a**	Rh_2_(OAc)_4_	PhH, 6 h	**3a**, 51 (55)	**4a**, 35 (36)	86 (91)
2^b^	**1a**; **2a**	Rh_2_(OAc)_4_	PhMe, 3 h	**3a**, 38	**4a**, 42	80
3^c^	**1a**; **2a**	Rh_2_(Oct)_4_	PhH, 2 h	**3a**, 35	**4a**, 23	58
4^b^	**1a**; **2a**	Rh_2_(Piv)_4_	PhH, 2 h	**3a**, 48 (58)	**4a**, 27 (33)	75 (91)
5^c^	**1a**; **2a**	Rh_2_(Piv)_4_	PhH, 1.5 h	**3a**, 21	**4a**, 32	53
6^b^	**1a**; **2b**	Rh_2_(Piv)_4_	PhH, 3 h	**3a’**, 44 (67)	**4a’**, 20 (30)	64 (97)
7^b^	**1b**; **2a**	Rh_2_(OAc)_4_	PhMe, 8 h^d^	**3b**, 27	**4b**, 25	52
8^b^	**1c**; **2a**	Rh_2_(OAc)_4_	PhMe, 6 h^d^	**3c**, 35	**4c**, 26	61
9^b^	**1d**; **2a**	Rh_2_(OAc)_4_	PhH, 8 h	**3d**, 30	**4d**, 24	54
10^b^	**1e**; **2a**	Rh_2_(OAc)_4_	PhMe, 6 h^d^	**3e**, 33	**4e**, 27	60

^a^Isolated yields are indicated in the table. The calculated yields based on reacted thioamide **1** are indicated in brackets. ^b^1.2 equiv of diazo compound **2** were used. ^c^2.1 equiv of diazo compound **2** were used. ^d^Reaction time in PhH was >20 h.

The same reaction with **2a** in toluene solution (reflux at 110 °C) instead of benzene lead to a decrease of the yield of thiophene **3a** (to 38%) in favour of thiophene **4a** (42%, Table 1, entry 2). However, even under these fairly severe conditions, it took from 3 to 6 h to achieve a complete conversion of diazomalonate, which is unusual for Rh(II)-catalyzed reactions of this diazo compound [26].

Application of more active catalysts like dirhodium tetraoctanoate or tetrapivalate makes it possible to reduce the reaction time at reflux in benzene to 2 h (Table 1, entries 3 and 4). In the case of dirhodium tetraoctanoate, the yields of the compounds **3a** and **4a** comprised 35 and 23% (Table 1, entry 3), while when using dirhodium tetrapivalate they were 48 and 27%, respectively (Table 1, entry 4). Thus the total yield of the main reaction products **3a**, **4a** increased to 75% (91% based on the reacted thioamide **1a**). However, a full conversion of thioamide **1a** in these experiments was not achieved in spite of the 1.2-fold excess of diazomalonate **2a** used in the process. Further increasing the amount of diazomalonate **2a** (up to 2.1 equiv) in the reaction with rhodium tetrapivalate resulted in the enhancement of the yield of thiophene **4a** (up to 32%), though the total yield of the main reaction products **3a** + **4a** therewith decreased to 53% (Table 1, entry 5).

Replacing diazomalonate **2a** by diethyl diazomalonate (**2b**) did not essentially change the yields of the main products, **3a’** and **4a’** (44 and 20%, respectively; Table 1, entry 6).

Thus the experiments with diazomalonate **2a** and thioacetamide **1a** demonstrated that the most appropriate conditions for the catalytic reaction of a diazo carbonyl compound with thioamides, were the employment of an 1.2-fold excess of diazoester **2**, Rh_2_(OAc)_4_ or Rh_2_(OPiv)_4_ as the catalysts, and performing the reaction at 80–110 °C.

To determine scope and limitations of the process, a series of experiments with diazomalonate **2a** and thioamides **1b**–**e** of different structure in the presence of Rh_2_(OAc)_4_ were carried out (Table 1, entries 7–10). It was found that within this series, the reactivity of thioamides depended significantly on the size of the substituent on the nitrogen atom of the thioamide group. Thus, decomposition of diazomalonate **2a** in the presence of thioacetamide **1d** bearing a bulky substituent occurred in boiling benzene within 8 h to afford thiophenes **3d** and **4d** in 30 and 24% yields (Table 1, entry 9). At the same time, thioamides **1b**,**c**,**e** with less bulky alkyl groups under similar conditions (reflux in benzene) reacted with diazomalonate **2a** much slower (Table 1; >20 h). That is why, to achieve satisfactory results, these reactions were carried out under reflux in toluene solution (110 °C; 6–8 h) giving rise to thiophenes **3b**,**c**,**e** and **4b**,**c**,**e** in 27–35% and 25–27% yields, respectively (Table 1, entries 7, 8 and 10).

**Figure 2:**
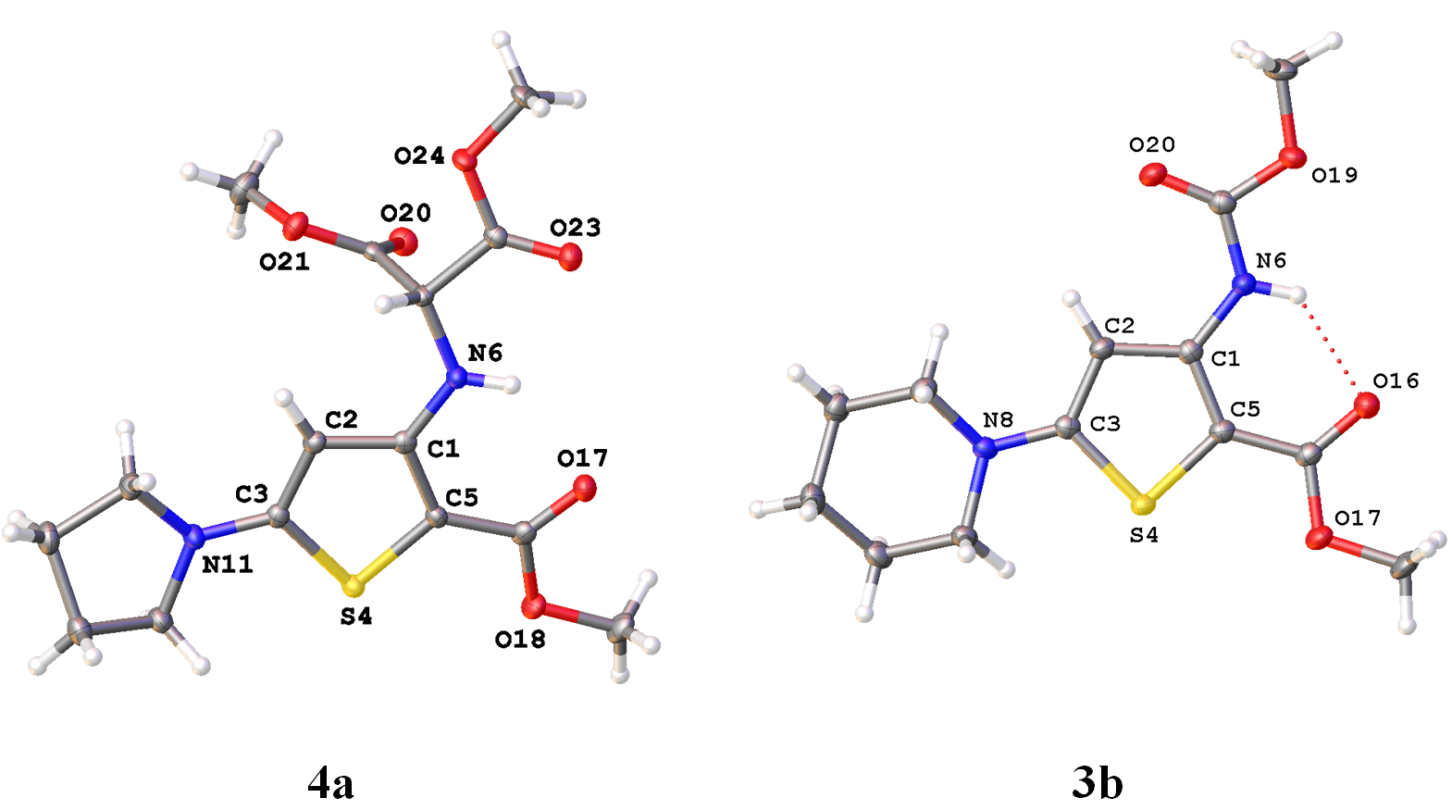
The structures of compounds **4a** and **3b** according to the data of X-ray analysis (Olex2 plot with 50% prohability level of ellipsoids).

The structures of reaction products **3** and **4** were established by a standard set of spectroscopic methods (^1^H, ^13^C NMR, HRMS) and, in the case of thiophenes **4a** and **3b**, was also confirmed by X-ray analysis (Figure 2). In the ^1^H NMR spectra of these compounds, the characteristic signals of NH protons are observed in the range of 9.84–9.68 and 7.96–7.80 ppm for **3a**–**e** and **4a**–**e**, respectively. The signals of thiophene H-4 and C-4 atoms in the ^1^H and ^13^C NMR spectra of compounds **3a**–**e** and **4a**–**e** are seen as singlets in the range of 6.95–6.58, 5.53–5.20 ppm and 97.7–93.7, 92.3–88.3 ppm, respectively.

Attempts to extend the reaction under study to the cyclic analogue of diazomalonate, diazoisopropylidenemalonate **2c**, were unsuccessful. Here, Rh_2_(OAc)_4_, Rh_2_(Piv)_4_ and Rh_2_(Oct)_4_ have been used as the catalysts, but with none of them a detectable conversion of the reagents was achieved. By and large this observation correlates with the literature data regarding relative inertness of the cyclic diazoester **2c** in Rh(II)-catalyzed reactions in comparison with diazomalonates and the other diazo compounds [26,52–53].

**Scheme 2:**
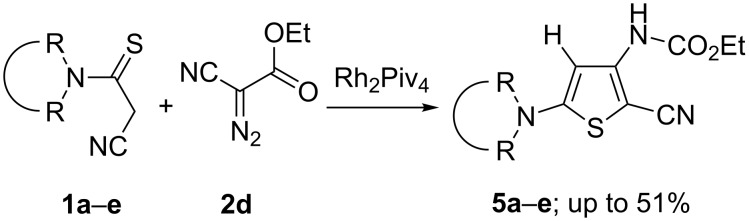
Rh(II)-Catalyzed reactions of α-diazocyanoacetic ester **2d** with α-cyanothioacetamides **1a–e**.

The investigation of diazocyanoacetic ester **2d** in reactions with thioamides **1**, catalyzed by dirhodium pivalate, had shown that they produced the structural analogues of carboxylates **3**, namely thiophenes **5**. At the same time no concurrent formation of aminomalonates of type **4** in the reaction media was detected in these processes. Furthermore, it turned out that diazocyanoester **2d** (in contrast to dialkyl diazomalonates **2a**,**b**) easily decomposed in the presence of dirhodium pivalate in methylene chloride even at room temperature (Scheme 2).

**Figure 3:**
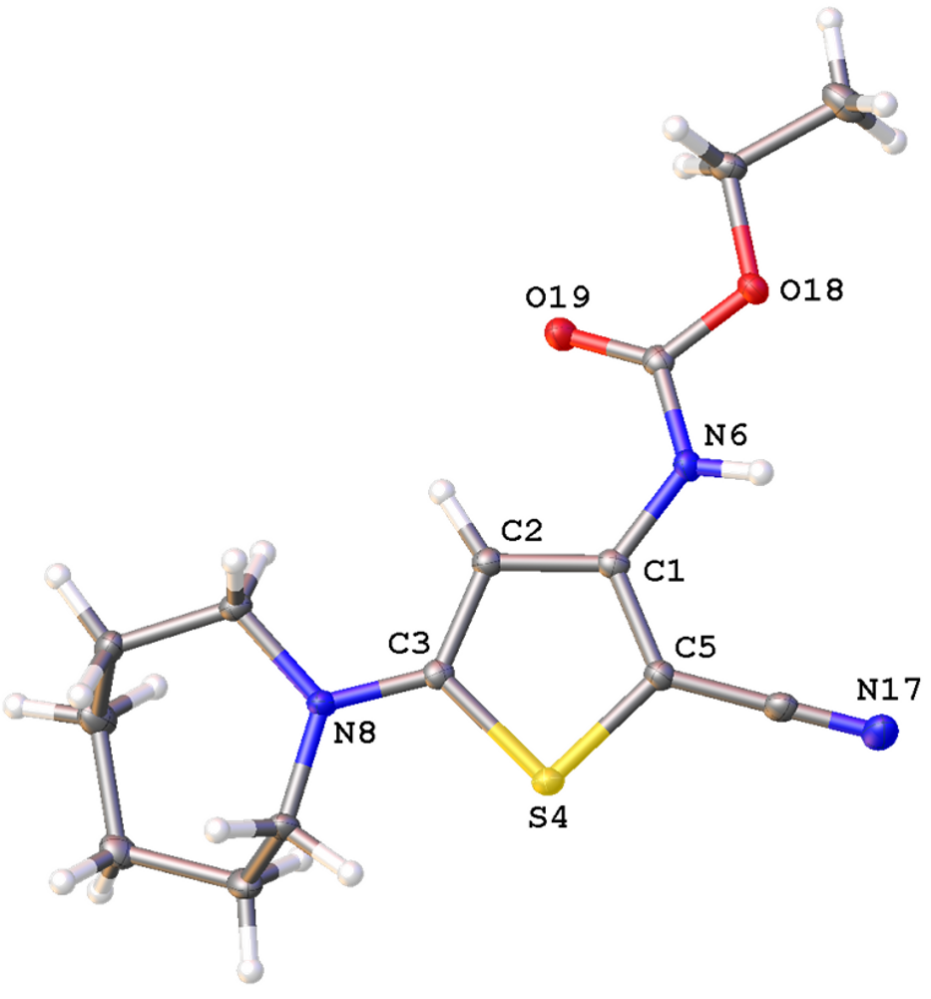
The structure of thiophene **5c** according to the data of X-ray analysis (Olex2 plot with 50% probability level of ellipsoids).

The catalytic reactions of diazocyanoester **2d** with the thioamides **1a**–**e** gave rise to thiophenes **5b**–**e** in the yields of up to 51%. The structure of the isolated thiophenes **5a**–**e** was established by a regular set of spectroscopic data, whereupon the structure of compound **5c** was in addition confirmed by X-ray analysis (Figure 3).

In the ^1^H NMR spectra of these compounds, the characteristic signals of NH protons are observed in the range of 7.15–7.09 ppm for **5a**–**e**. The signals of thiophene H-4 and C-4 atoms in the ^1^H and ^13^C spectra of the compounds **5a**–**e** are seen as the singlets at the range of 6.40–6.78 and 96.8–92.8 ppm, respectively.

Hence it was established that the main products of diazoesters **2a**,**b**,**d** in reactions with thioacetamides **1a**–**e**, catalyzed by rhodium complexes, were sulfur-containing heterocycles, 5-amino-3-(alkoxycarbonylamino)thiophene-2-carboxylates **3**, 2-(5-amino-2-methoxycarbonylthiophen-3-yl)aminomalonates **4** and (2-cyano-5-aminothiophen-3-yl)carbamates **5**, which could be formally referred to as the derivatives of carbamates **3**, **5** and heteroaromatic amines **4**.

**Scheme 3:**
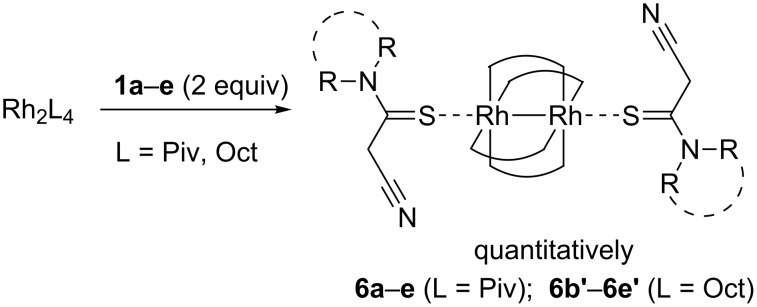
Interaction of thioacetamide **1e** with dirhodium pivalate to produce complex **6e**.

**Figure 4:**
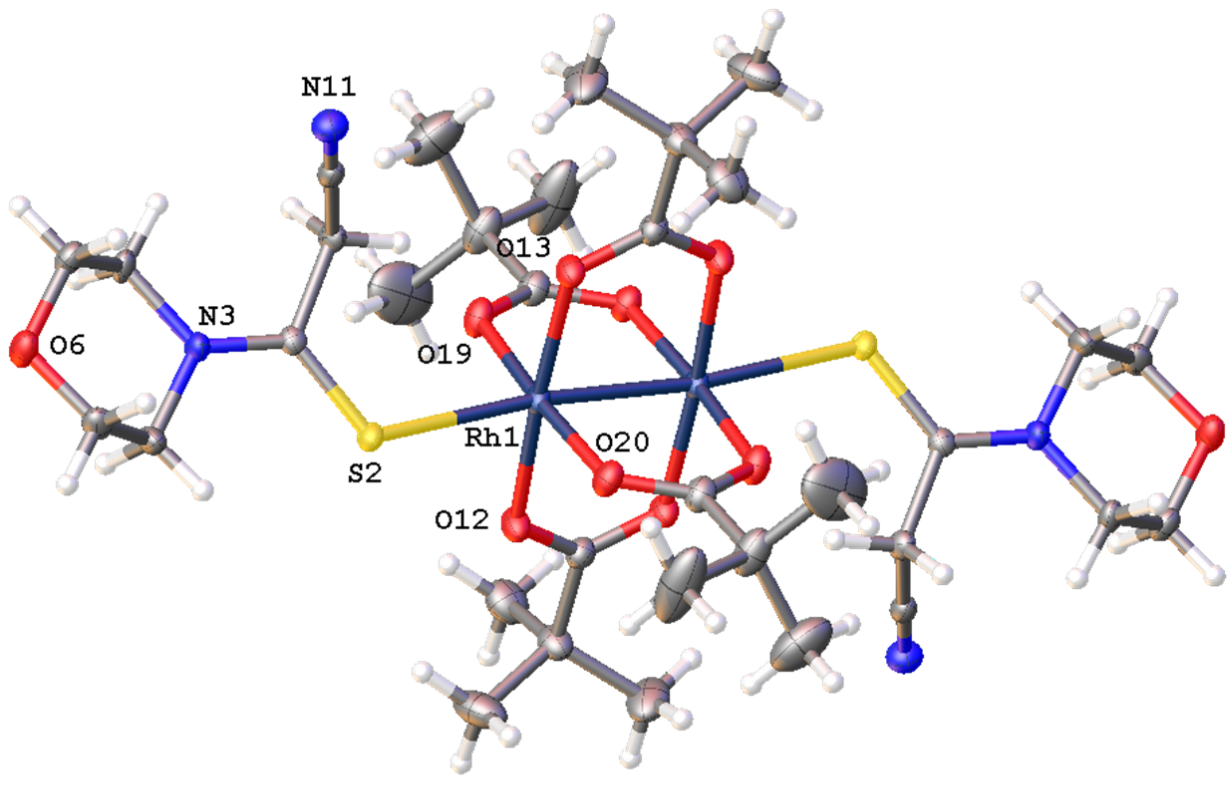
The structure of the complex **6e** according to the data of X-ray analysis (Olex2 plot with 50% probability level of ellipsoids).

To elucidate the reasons for the low reactivity of diazoesters **2a**,**b** with thioamides **1** in the considered catalytic processes, the interaction of thioacetamides **1a**–**e** with Rh_2_(Piv)_4_ and Rh_2_(Oct)_4_ was studied (Scheme 3). The reaction was carried out in methylene chloride at room temperature using a 2-fold excess of thioacetamides **1**. It produced almost quantitatively dark green adducts **6a**–**e**, **6b’**–**e’**, which structure was confirmed by analytical methods, X-ray analysis for complex **6e** (Figure 4), and by analogy with literature data [54–55]. According to the literature findings, dirhodium(II) tetracarboxylates can form 1:2 adducts with such neutral ligands as thioamides [54–55] and thioesters [56–58]. The reactions give adducts with up to quantitative yields which is an evidence of high reactivity of rhodium complexes in these processes [54–55]. This literature data brought us to the suggestion that low reactivity of diazoesters **2** toward thioamides **1** in the presence of Rh(II)-catalysts is caused by binding the two latter chemicals to furnish the adducts of the type **6** where both axial active sites of the catalyst are inaccessible for further interaction with diazoesters **2**.

**Scheme 4:**
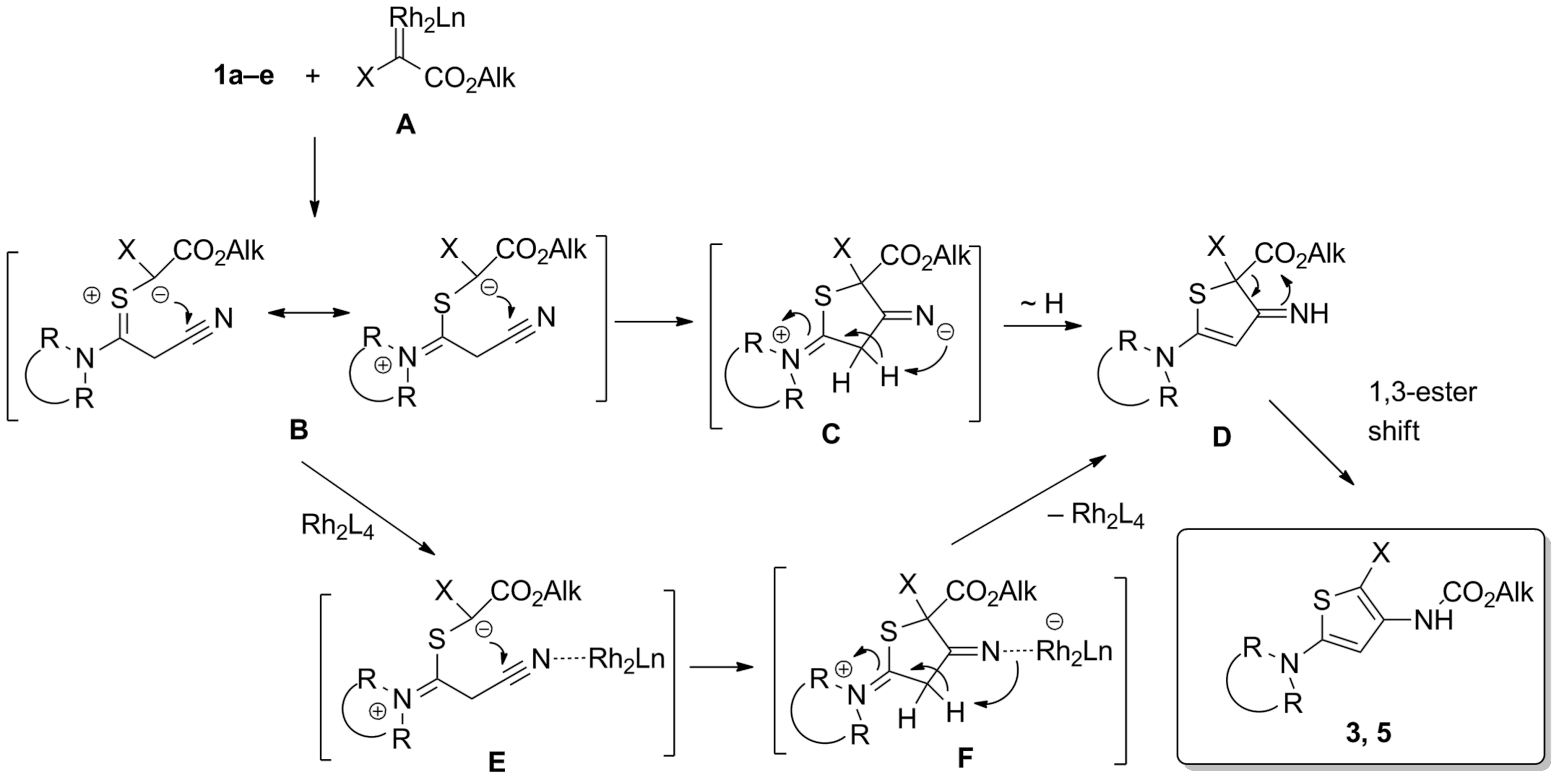
The assumed mechanism for the formation of thiophenes **3**, **5**.

To verify this assumption, a separate experiment was performed by the example of dimethyl diazomalonate (**2a**) decomposition with the obtained Rh-complex **6e**. And it was demonstrated that under these conditions the same (as with Rh_2_L_4_) thiophenes **3e** and **4e** were formed. In this connection it is believed that at the elevated temperatures (80–110 °C) a partial dissociation of these complexes into original components **1** and Rh-catalyst takes place that directs the whole process into the ‘carbenoid channel’, as illustrated in Scheme 4. Initially generated from diazoester **2** carbenoid **A** attacks the sulfur atom of thioamide **1** to give the key intermediate S-ylide **B** [36–38,59–60], which is stabilized by ‘thioamide resonance’ [36–38]. The anion center of S-ylide **B** then attacks the carbon atom of the cyano group leading to intermediate **C** [61] which further turns into (imino)dihydrothiophene **D** through intra- or intermolecular transfer of a proton from the activated CH_2_ group. And finally a 1,3-shift of the alkoxycarbonyl group [62] in intermediate **D** completes the process to furnish thiophenes **3** or **5**.

Principally, thiophenes **3** and **5** could be derived from the S-ylide **B** in a somewhat different way, as for instance: coordination **E** of cyano group in S-ylide with a rhodium catalyst [63] gives rise to zwitterion **F** with a negative charge located on the rhodium atom, followed by recovery of the catalyst, proton transfer and, finally, 1,3-shift of alkoxycarbonyl group in the intermediate imine **D** to produce thiophenes **3** and **5**.

However, this pathway seems to be less probable, since intramolecular cyclization **B** → **C** should have lower activation energy relative to intermolecular interaction of C=S ylide with the rhodium catalyst.

**Scheme 5:**
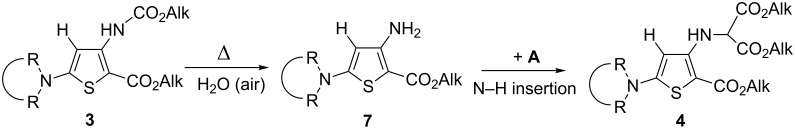
The plausible mechanism for the formation of thiophenes **4**.

Within the adopted general scheme, the occurrence of thiophenes **4** could be rationalized by partial hydrolysis of carbamates **3** under the reaction conditions with the initial formation of the primary heteroaromatic amines **7**. The latter then interact with carbenoids **A**, to produce thiophenes **4** through an ordinary N–H insertion process [6–13] (Scheme 5).

To the best of our knowledge, the discovered processes are the first examples of intramolecular reactions of thiocarbonyl ylides with cyano groups, acting as an electrophile, with subsequent 1,3-migration of the alkoxycarbonyl moiety to produce the corresponding thiophenes. These reactions represent a new facile one-pot preparative method for the synthesis of 2,4-(diamino)thiophenes from the available reagents. The known findings on the synthesis of (diamino)thiophenes are limited to several articles on the transformations of 2-chlorothioacet-amides [64–65] and reactions of oxathioles [66] or 5-methylthiophenes [67] with amines.

Bearing a potential practical application of these compounds in mind, it is worthy to note that the structures comprising a 2,4-(diamino)thiophene fragment possess photosensitivity and hence could be used in OLED devices [68]. Also these compounds display high nucleophilicity in reactions with electrophiles which are often accompanied by a variety of thiophene heterocycle transformations, and hence could be successfully used for their further functionalization [69].

## Conclusion

In summary, the investigation of Rh(II)-catalyzed domino reactions of acyclic diazoesters with α-cyanothioacetamides lead to elaboration of a new approach for the synthesis of multisubstituted thiophenes. These reactions could be applied for the preparation of 5-amino-3-(alkoxycarbonylamino)thiophen-2-carboxylates **3** (yields up to 67%), 2-(5-amino-2-methoxycarbonylthiophen-3-yl)aminomalonates **4** (up to 36%) and (2-cyano-5-aminothiophen-3-yl)carbamates **5** (up to 51%). It was also shown that α-cyanothioacetamides react with dirhodium carboxylates to form rather stable 2:1 complexes, that somewhat decreases the efficiency of the catalytic process at moderate temperatures (20–30 °C).

## Supporting Information

File 1 NMR spectra of all new compounds and data of X-ray analysis.

File 2 Experimental procedures and characterization data of all new compounds.
