# Supplementary material for: Rh(II)-mediated domino [4 + 1]-annulation of α-cyanothioacetamides using diazoesters: A new entry for the synthesis of multisubstituted thiophenes
Source: Beilstein J Org Chem. 2017 Nov 30;13:2569–76. doi: 10.3762/bjoc.13.253 (PMC5727864; doi:10.3762/bjoc.13.253)

Supporting Information

for

Rh(II)-mediated domino [4 + 1]-annulation of α-cyanothioacetamides using diazoesters: A new entry for the synthesis of multisubstituted thiophenes

Jury J. Medvedev,[1] Ilya V. Efimov,[2] Yuri M. Shafran,[2] Vitaliy V. Suslonov,[3] Vasiliy A. Bakulev*[2] and Valerij A. Nikolaev *[1]

Address: [1]Department of Organic Chemistry, St-Petersburg State University, 26 University pr., 198504, Saint-Petersburg, Russia, [2]Institute of Chemistry and Technology, Ural Federal University, 19 Mira Str., 620002, Ekaterinburg, Russia and [3]Center for X-ray Diffraction Studies, St-Petersburg State University, 26 University pr., 198504, Saint-Petersburg, Russia.

Email: V. A. Nikolaev* - valerij.nikolaev@gmail.com; V. A. Bakulev* - v.a.bakulev@urfu.ru

*Corresponding author

NMR spectra of all new compounds and data of X-ray analysis

Table of content

^{1}H NMR spectrum for compound **3a**	3
^{13}C NMR spectrum for compound **3a**	4
^{1}H NMR spectrum for compound **3a'**	5
^{13}C NMR spectrum for compound **3a'**	6
^{1}H NMR spectrum for compound **3b**	7
^{13}C NMR spectrum for compound **3b**	8
^{1}H NMR spectrum for compound **3c**	9
^{13}C NMR spectrum for compound **3c**	10
^{1}H NMR spectrum for compound **3d**	11
^{13}C NMR spectrum for compound **3d**	12
^{1}H NMR spectrum for compound **3e**	13
^{13C} NMR spectrum for compound **3e**	14
^{1}H NMR spectrum for compound **4a**	15
^{1c}C NMR spectrum for compound **4a**	16
^{1}H NMR spectrum for compound **4a'**	17
^{13}C NMR spectrum for compound 4**a'**	18
^{1}H NMR spectrum for compound **4b**	19
^{13}C NMR spectrum for compound **4b**	20
^{1}H NMR spectrum for compound **4c**	21
^{13}C NMR spectrum for compound **4c**	22
^{1}H NMR spectrum for compound **4d**	23
^{1c}C NMR spectrum for compound **4d**	24
^{1}H NMR spectrum for compound **4e**	25
^{13}C NMR spectrum for compound **4e**	26
^{1}H NMR spectrum for compound **5a**	27
^{13}C NMR spectrum for compound **5a**	28
^{1}H NMR spectrum for compound **5b**	29
^{13}C NMR spectrum for compound **5b**	30
^{1}H NMR spectrum for compound **5c**	31
^{13}C NMR spectrum for compound **5c**	32
^{1}H NMR spectrum for compound **5d**	33
^{13}C NMR spectrum for compound **5d**	34
^{1}H NMR spectrum for compound **5e**	35
^{13}C NMR spectrum for compound **5e**	36
^{1}H NMR spectrum for compound **6a**	37
^{1}H NMR spectrum for compound **6b**	38
^{1}H NMR spectrum for compound **6b'**	39
^{1}H NMR spectrum for compound **6c**	40
^{1}H NMR spectrum for compound **6c'**	41
^{1}H NMR spectrum for compound **6d**	42
^{1}H NMR spectrum for compound **6d'**	43
^{1}H NMR spectrum for compound **6e**	44
^{1}H NMR spectrum for compound **6e'**	45
Crystal structure determination for compounds **3b**, **4a**, **5c** and **7e**	46
checkCIF/PLATON report for compound **3b**	49
checkCIF/PLATON report for compound **4a**	52
checkCIF/PLATON report for compound **5c**	55
checkCIF/PLATON report for compound **7e**	56

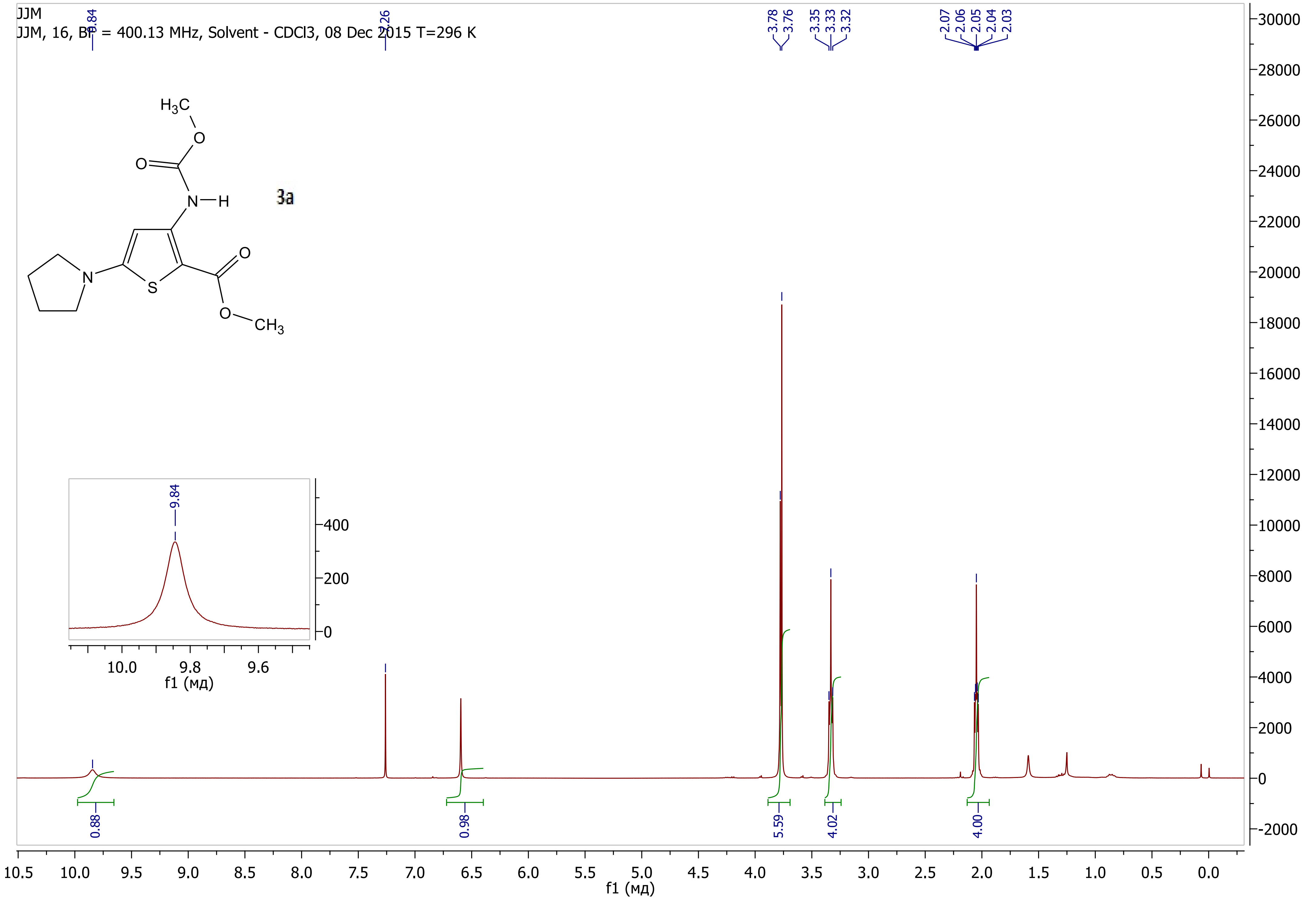
JJM
JJM, 16, BF = 400.13 MHz, Solvent - CDCl3, 08 Dec 2015 T=296 K
9.84
7.26
3.78
3.76
3.35
3.33
3.32
2.07
2.06
2.05
2.04
2.03
3a
0.88
0.98
5.59
4.02
4.00
f1 (мд)

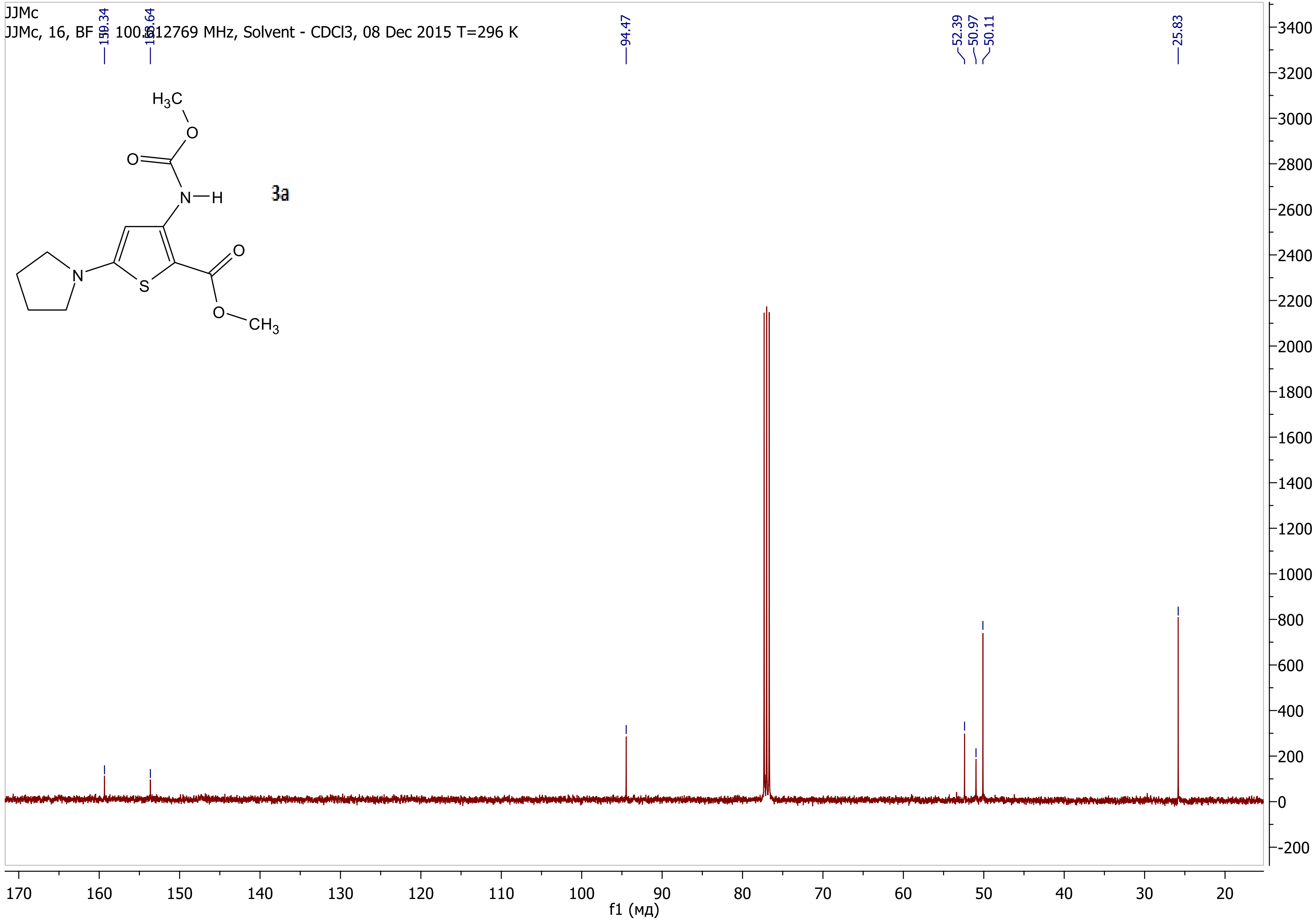
JJMc
JJMc, 16, BF 100.612769 MHz, Solvent - CDCl3, 08 Dec 2015 T=296 K
159.34
153.64
94.47
52.39
50.97
50.11
25.83
H_3C
O
O
N—H
O
S
N
O
CH_3
3a
f1 (мд)

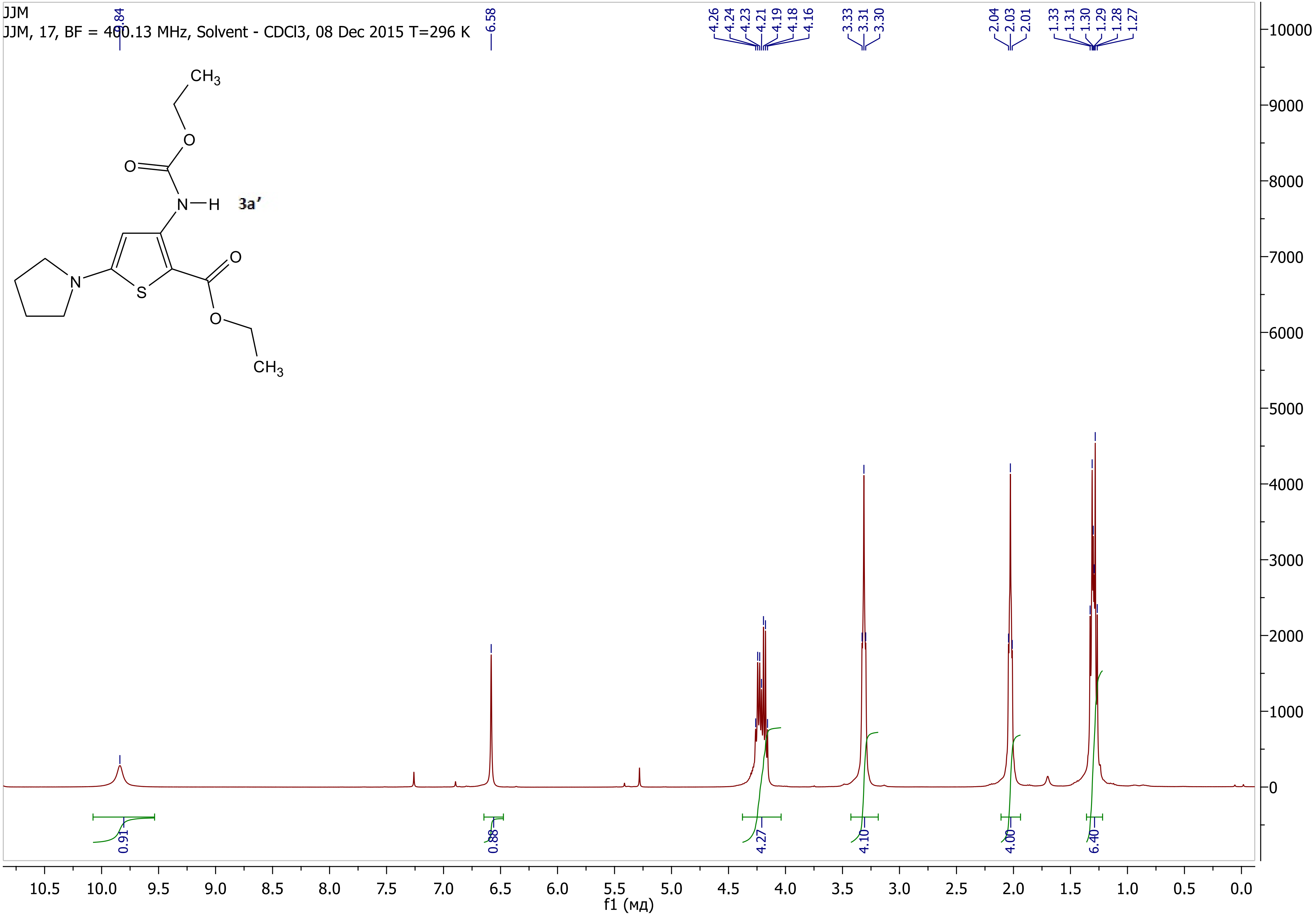
JJM
JJM, 17, BF = 400.13 MHz, Solvent - CDCl3, 08 Dec 2015 T=296 K
9.84
6.58
4.26 4.24 4.23 4.21 4.19 4.18 4.16
3.33 3.31 3.30
2.04 2.03 2.01
1.33 1.31 1.30 1.29 1.28 1.27
3a'
0.91
0.88
4.27
4.10
4.00
6.40
f1 (мд)

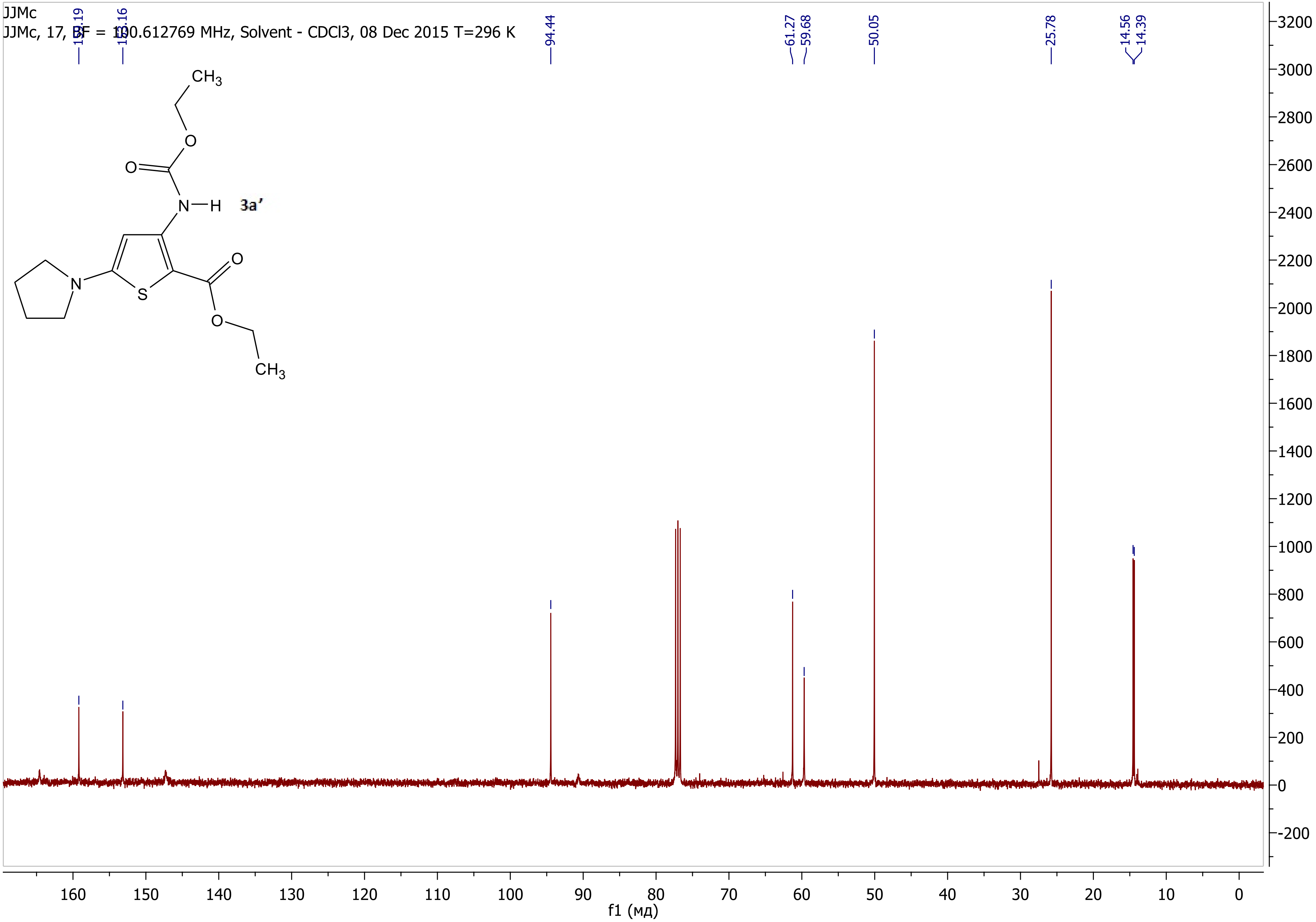

JJMc
JJMc, 17, BF = 100.612769 MHz, Solvent - CDCl3, 08 Dec 2015 T=296 K
159.19
153.16
94.44
61.27
59.68
50.05
25.78
14.56
14.39
CH3
O
O
N—H
3a’
O
N
S
O
CH3
f1 (мд)

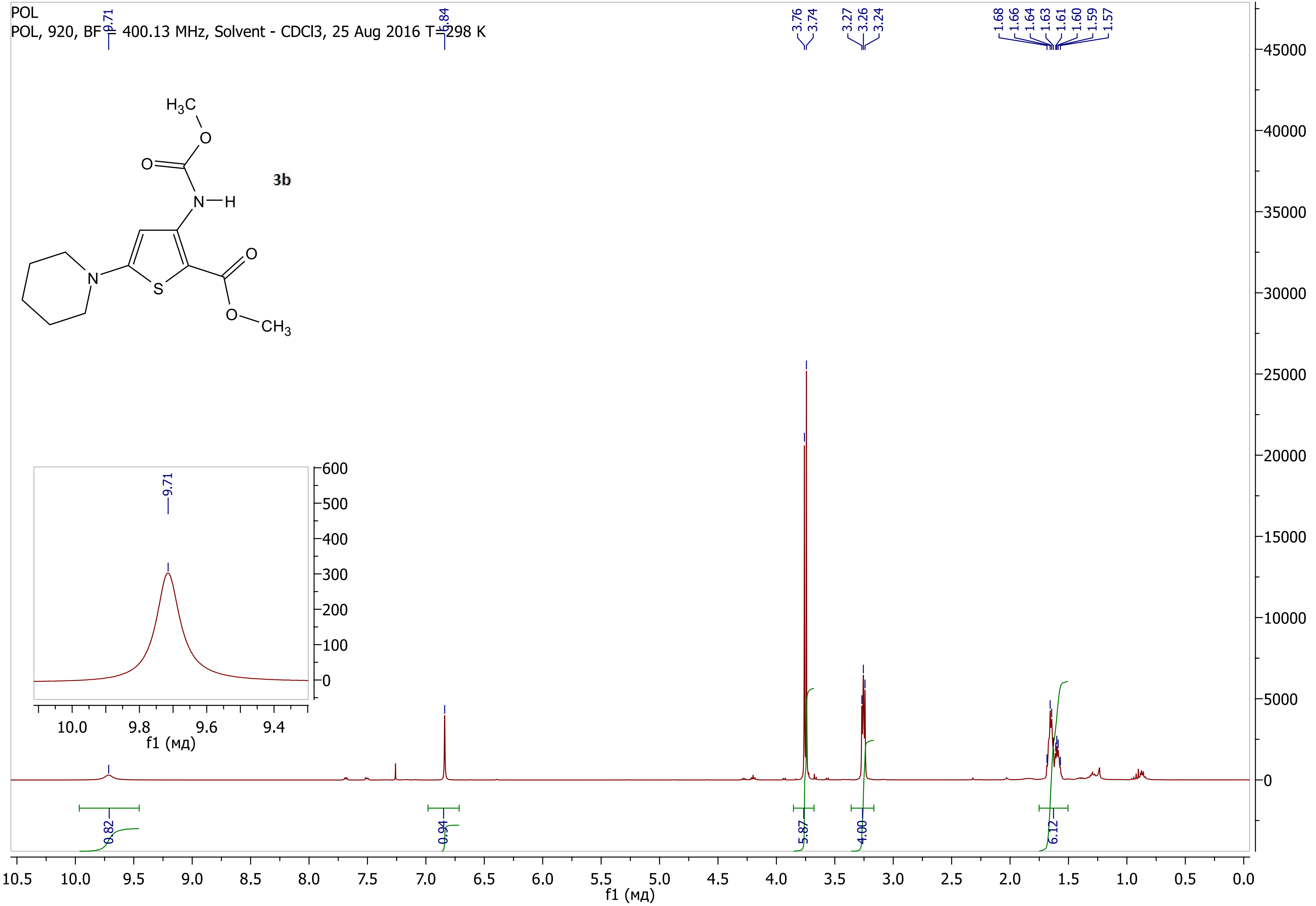
POL
POL, 920, BF = 400.13 MHz, Solvent - CDCl3, 25 Aug 2016 T=298 K
9.71
6.84
3.76
3.74
3.27
3.26
3.24
1.68
1.66
1.64
1.63
1.61
1.60
1.59
1.57
3b
0.82
0.94
5.87
4.00
6.12
f1 (мд)

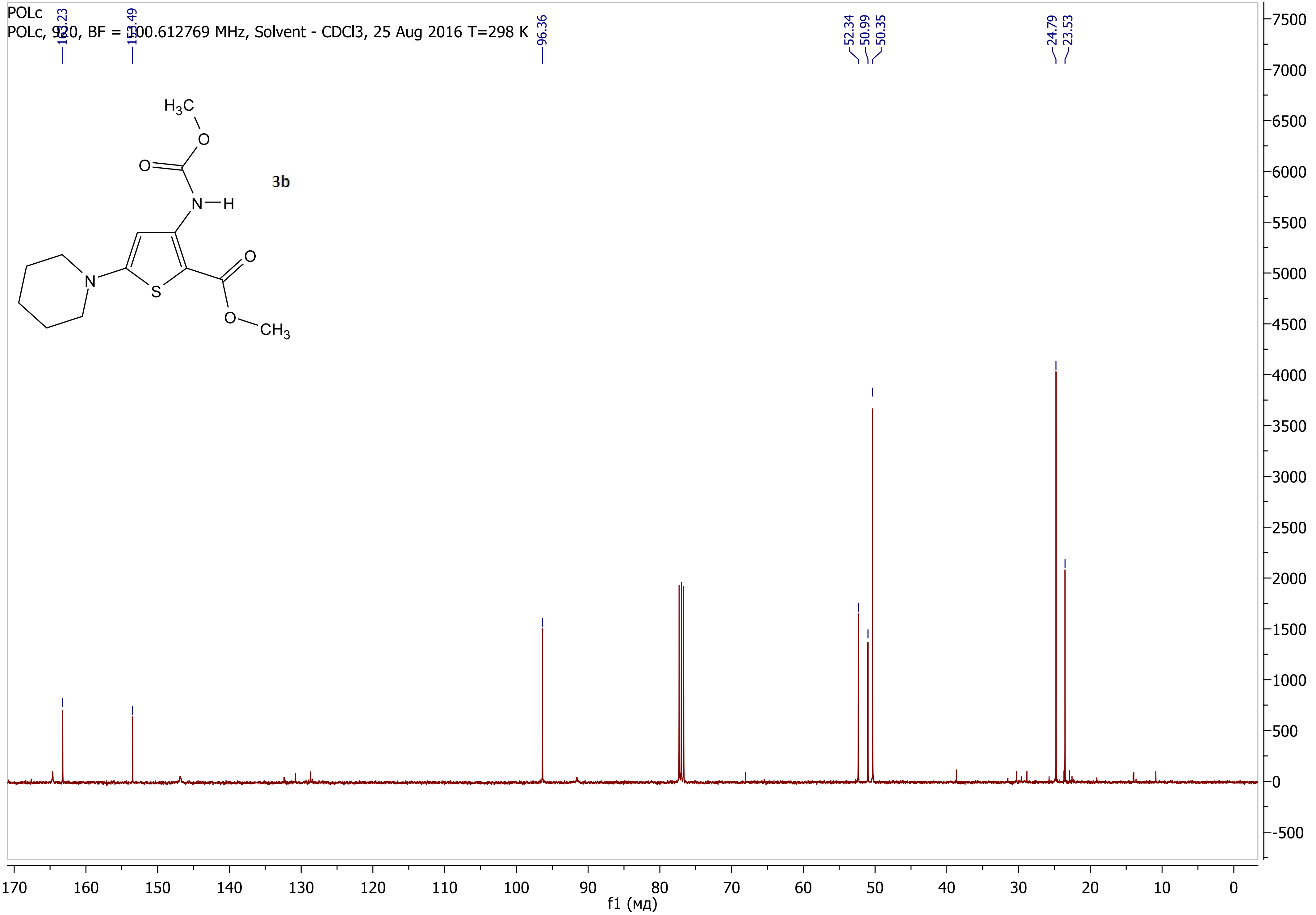
POLc
POLc, 920, BF = 100.612769 MHz, Solvent - CDCl3, 25 Aug 2016 T=298 K
163.23
153.49
96.36
52.34
50.99
50.35
24.79
23.53
3b
f1 (мд)

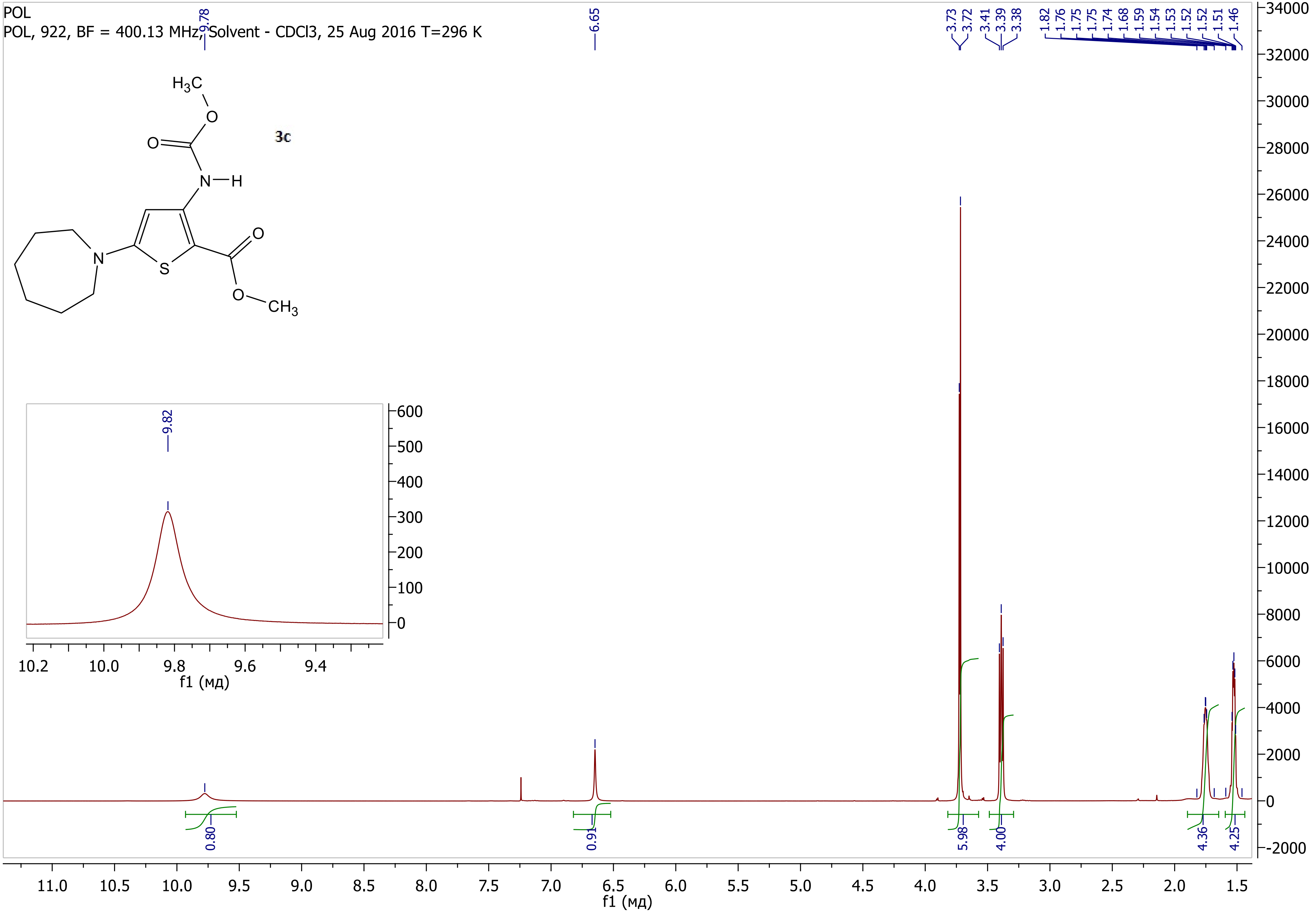
POL
POL, 922, BF = 400.13 MHz, Solvent - CDCl3, 25 Aug 2016 T=296 K
3c
9.78
6.65
3.73
3.72
3.41
3.39
3.38
1.82
1.76
1.75
1.75
1.74
1.68
1.59
1.54
1.53
1.52
1.52
1.51
1.46
9.82
0.80
0.91
5.98
4.00
4.36
4.25
f1 (мд)

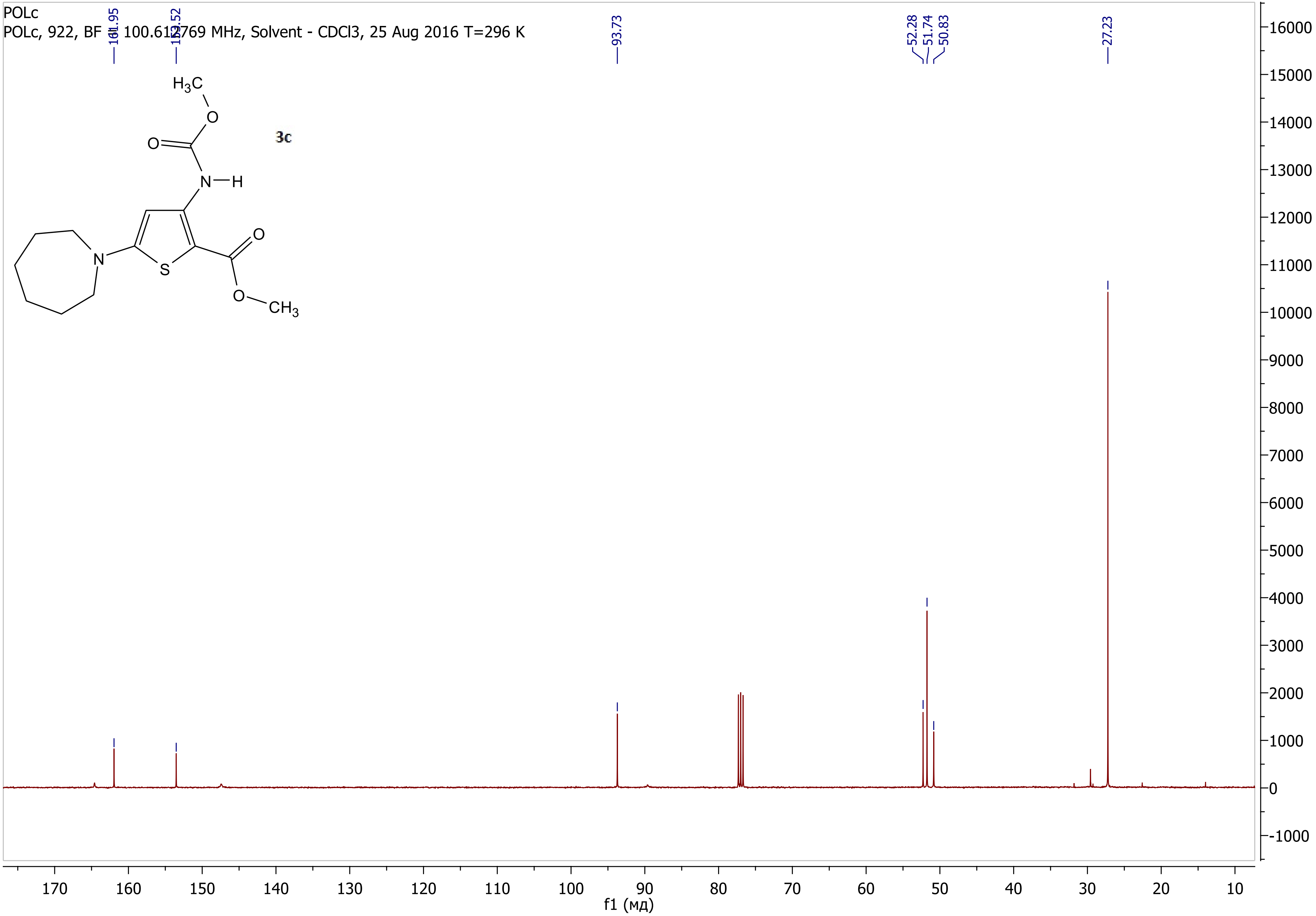

POLc
POLc, 922, BF = 100.612769 MHz, Solvent - CDCl3, 25 Aug 2016 T=296 K
161.95
153.52
93.73
52.28
51.74
50.83
27.23
3c
f1 (мд)

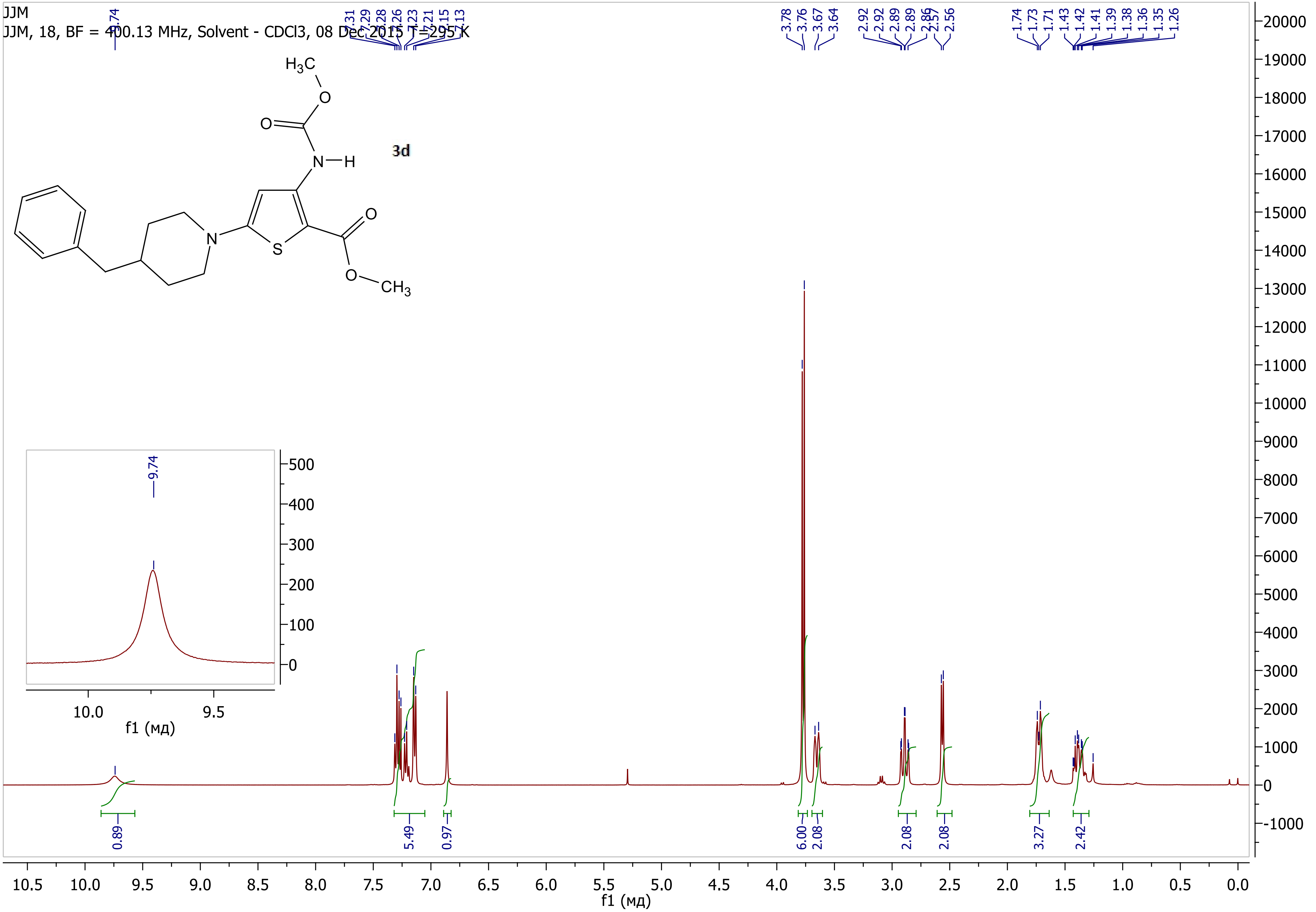
JJM
JJM, 18, BF = 400.13 MHz, Solvent - CDCl3, 08 Dec 2015 T=295 K
3d
9.74
7.31 7.29 7.28 7.26 7.23 7.21 7.15 7.13
3.78 3.76 3.67 3.64
2.92 2.92 2.89 2.89 2.86 2.57 2.56
1.74 1.73 1.71 1.43 1.42 1.41 1.39 1.38 1.36 1.35 1.26
0.89 5.49 0.97 6.00 2.08 2.08 2.08 3.27 2.42
f1 (мд)

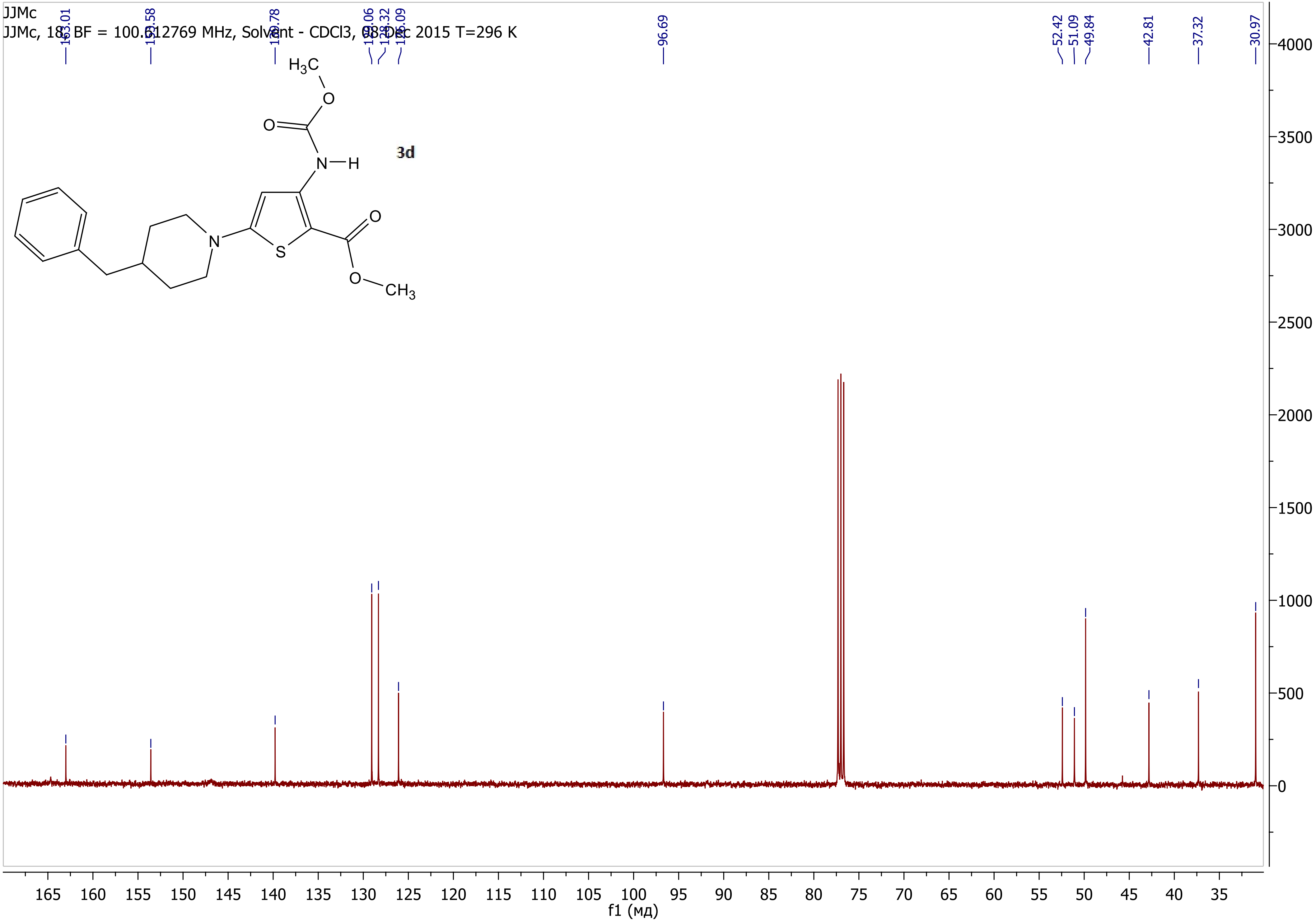

JJMc
JJMc, 18, BF = 100.612769 MHz, Solvent - CDCl3, 08 Dec 2015 T=296 K
163.01
153.58
139.78
129.06
128.32
126.09
96.69
52.42
51.09
49.84
42.81
37.32
30.97
3d
H3C
O
O
N—H
N
S
O
O
CH3
4000
3500
3000
2500
2000
1500
1000
500
0
165 160 155 150 145 140 135 130 125 120 115 110 105 100 95 90 85 80 75 70 65 60 55 50 45 40 35
f1 (мд)

POL
POL, 960, BF = 400.13 MHz, Solvent - CDCl3, 21 Oct 2016 T=298 K

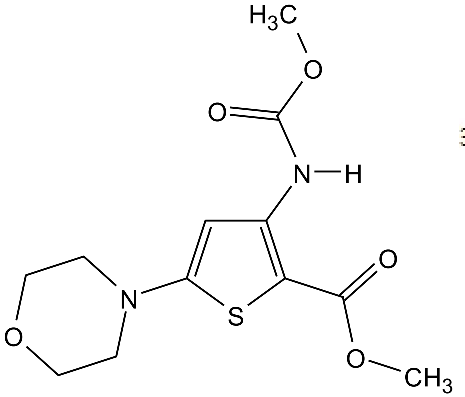

3e

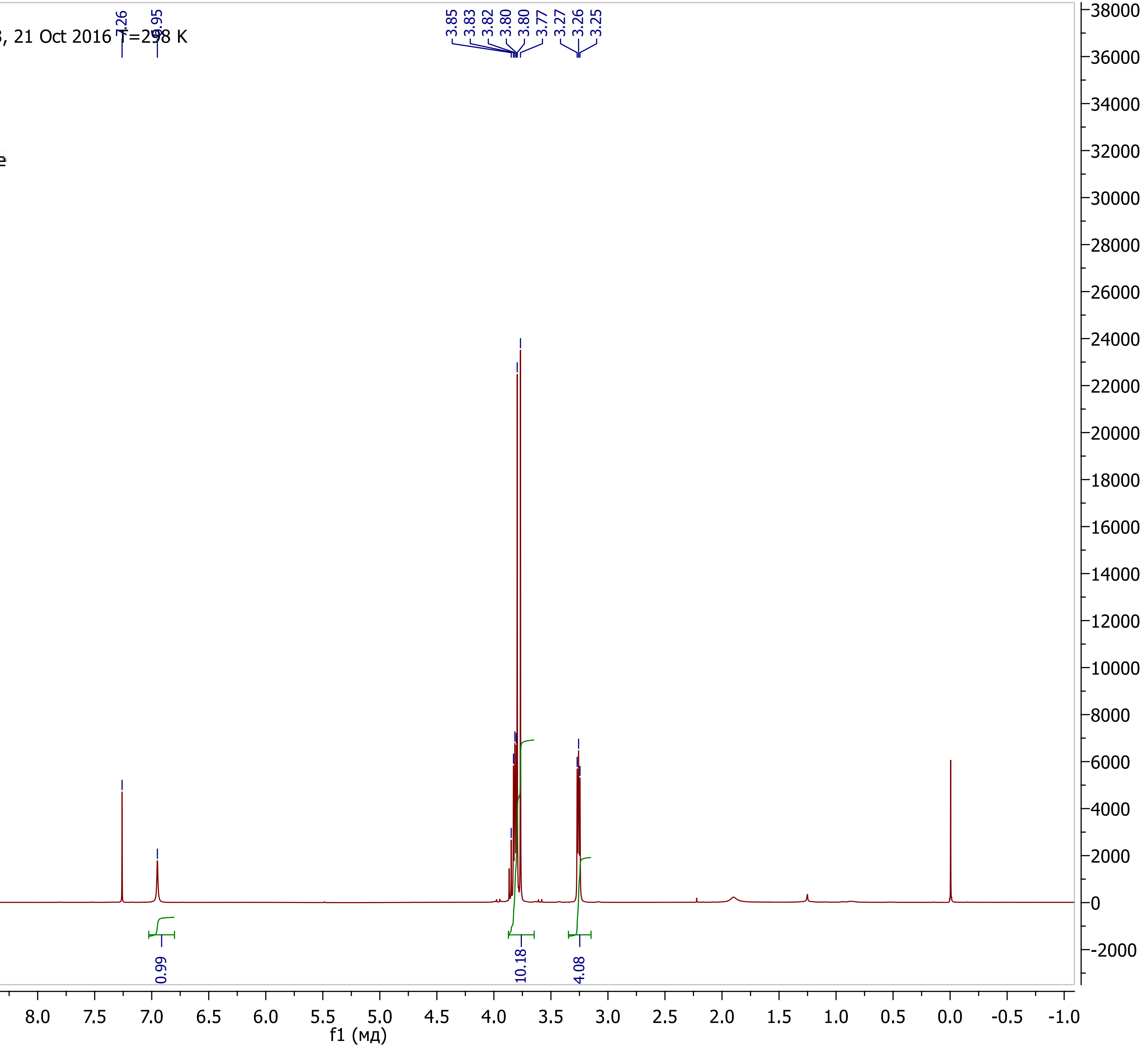

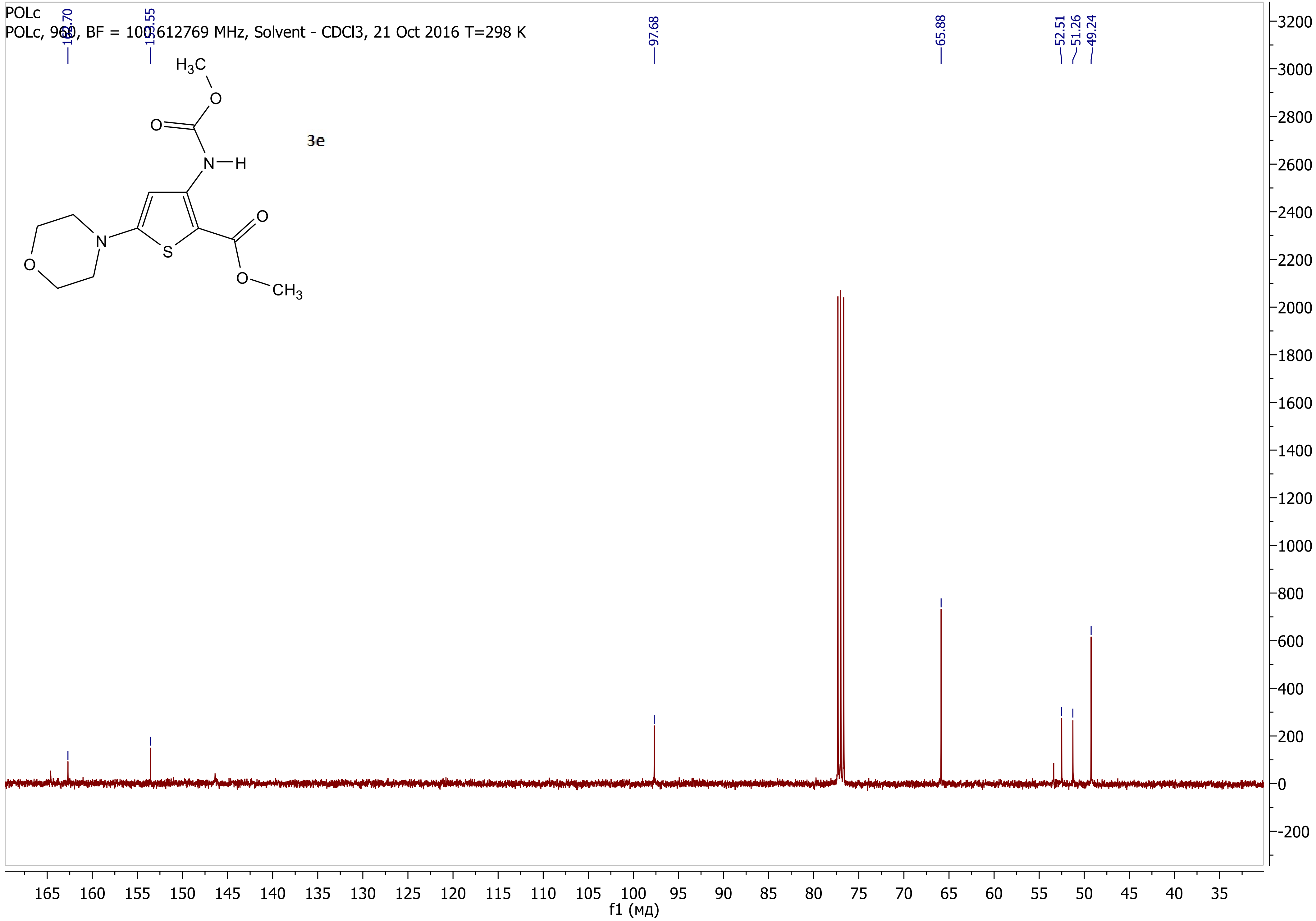

POLc
POLc, 960, BF = 100.612769 MHz, Solvent - CDCl3, 21 Oct 2016 T=298 K
97.68
65.88
52.51
51.26
49.24
3e
f1 (мд)

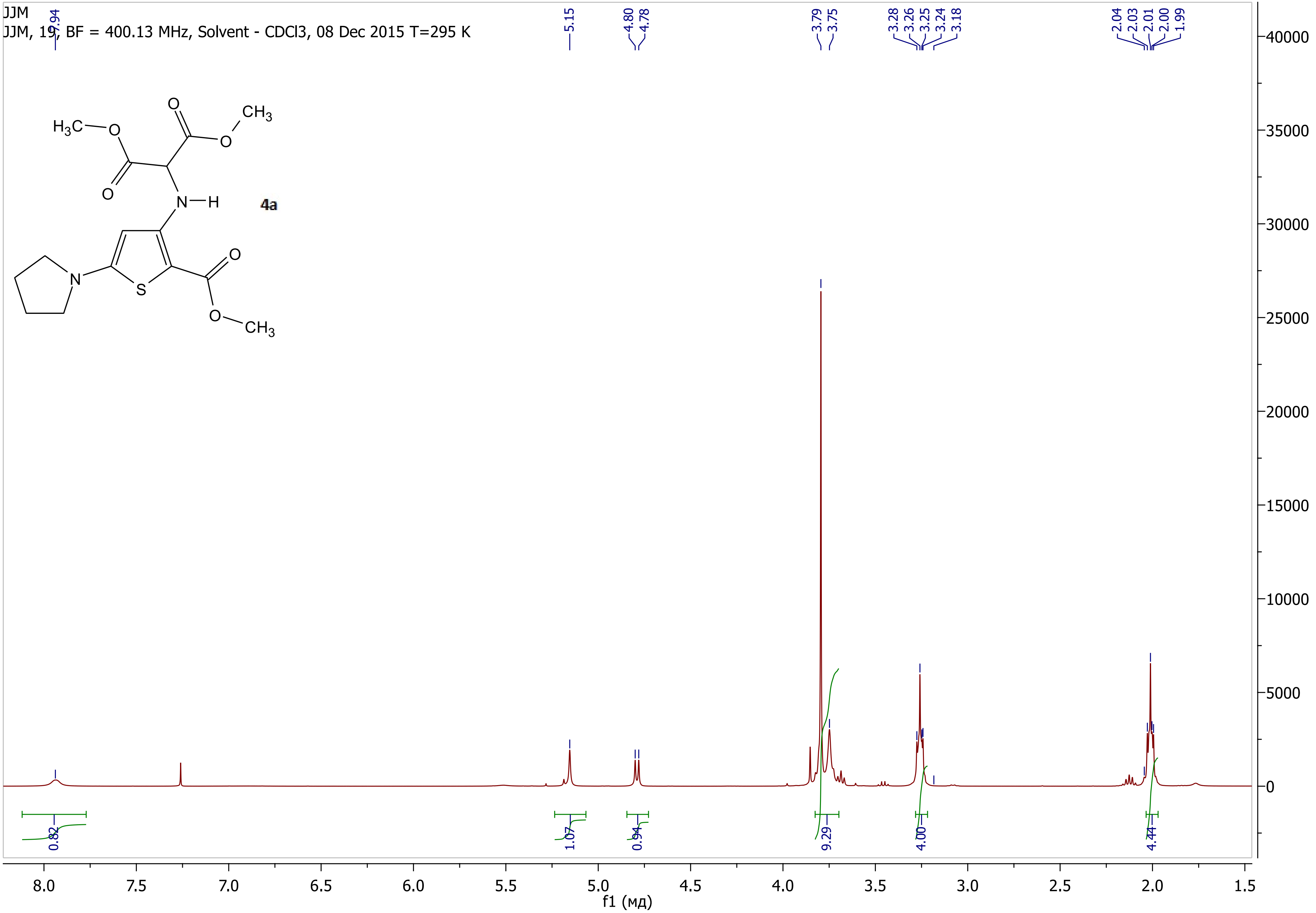
JJM
JJM, 19, BF = 400.13 MHz, Solvent - CDCl3, 08 Dec 2015 T=295 K
7.94
5.15
4.80
4.78
3.79
3.75
3.28
3.26
3.25
3.24
3.18
2.04
2.03
2.01
2.00
1.99
4a
0.82
1.07
0.94
9.29
4.00
4.44
f1 (мд)

JJMc
JJMc 19, BF = 100.612769 MHz, Solvent - CDCl3, 08 Dec 2015 T=296 K

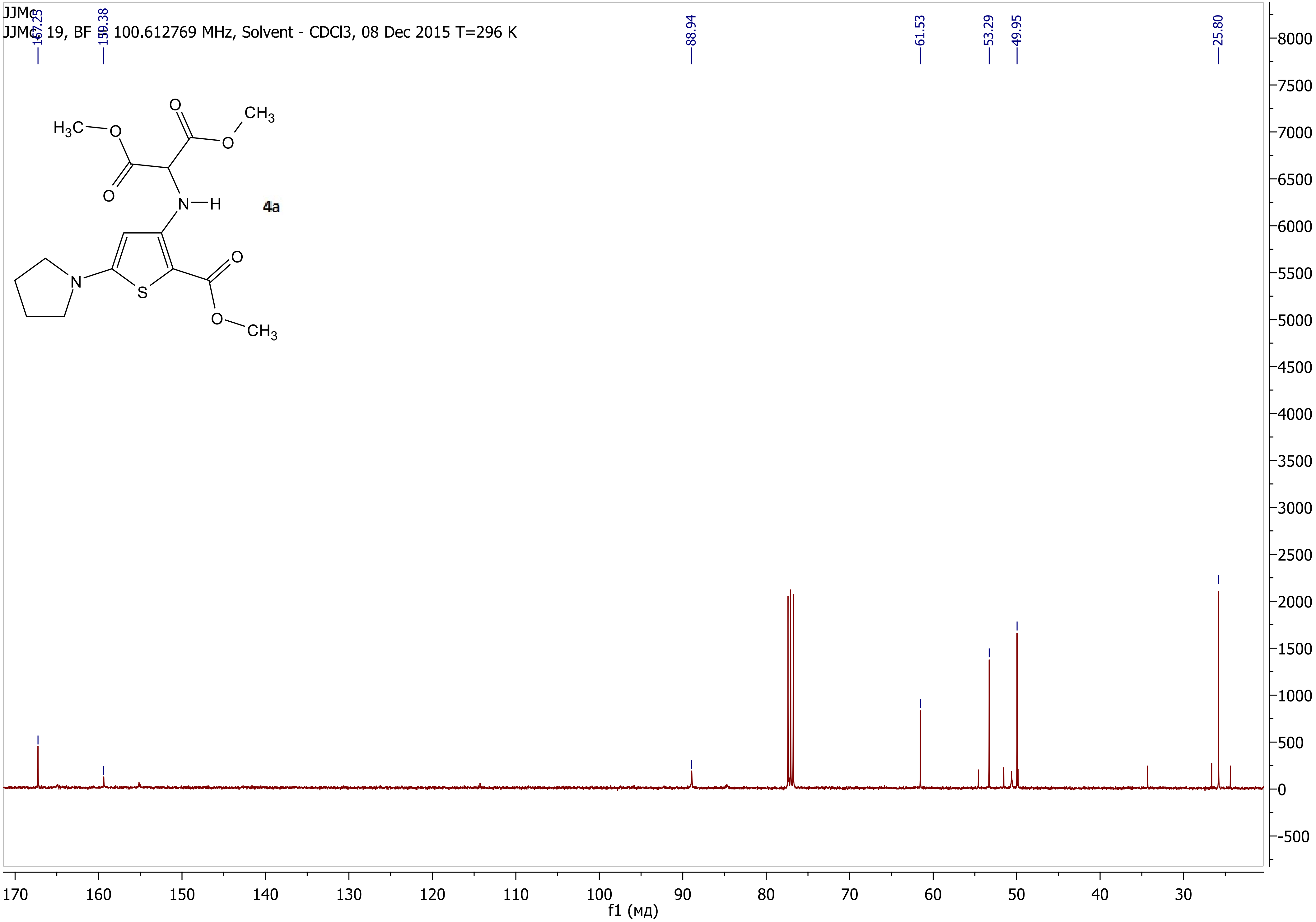

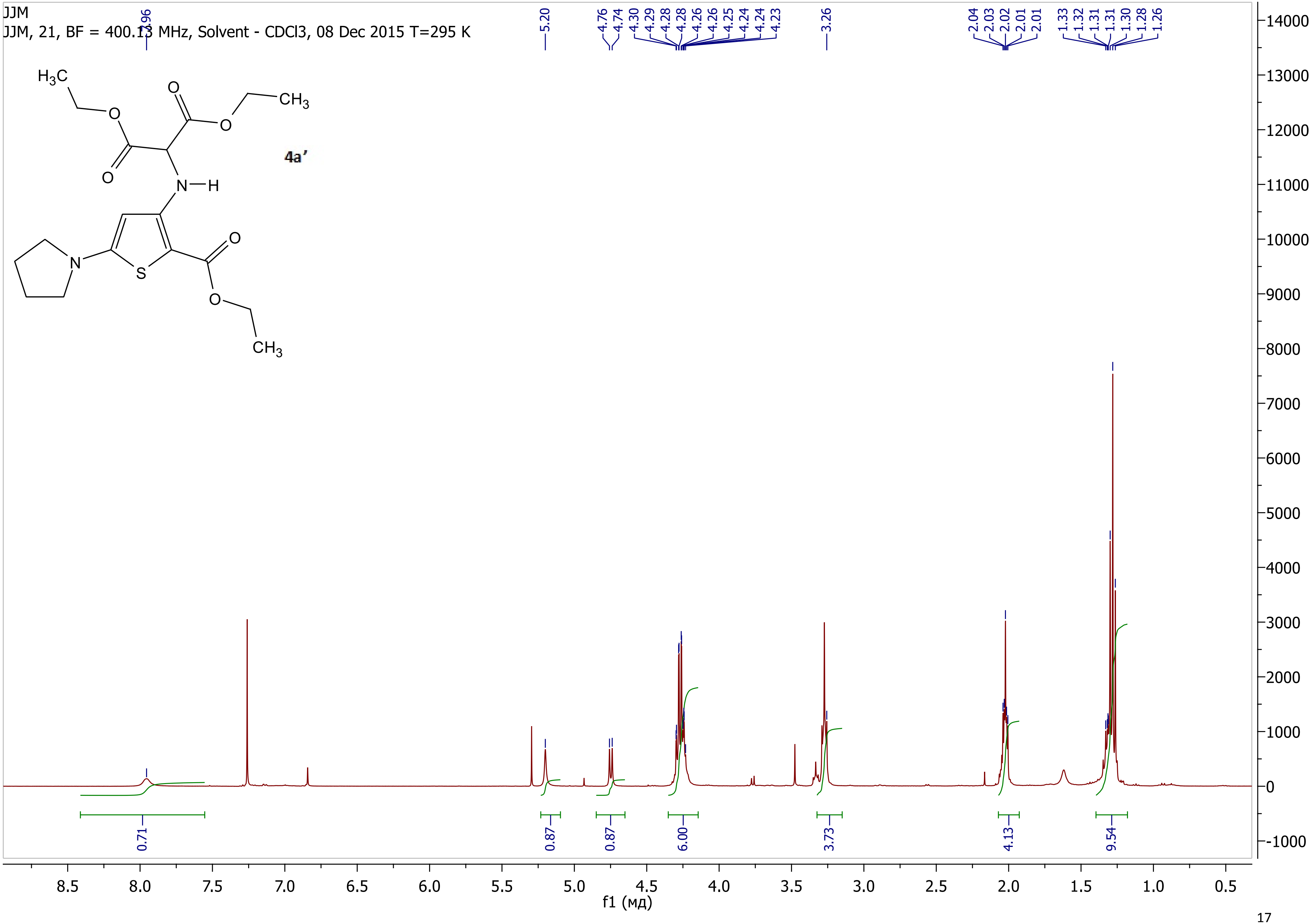
JJM
JJM, 21, BF = 400.13 MHz, Solvent - CDCl3, 08 Dec 2015 T=295 K
7.96
5.20
4.76
4.74
4.30
4.29
4.28
4.28
4.26
4.26
4.25
4.24
4.24
4.23
3.26
2.04
2.03
2.02
2.01
2.01
1.33
1.32
1.31
1.31
1.30
1.28
1.26
4a’
0.71
0.87
0.87
6.00
3.73
4.13
9.54
f1 (мд)

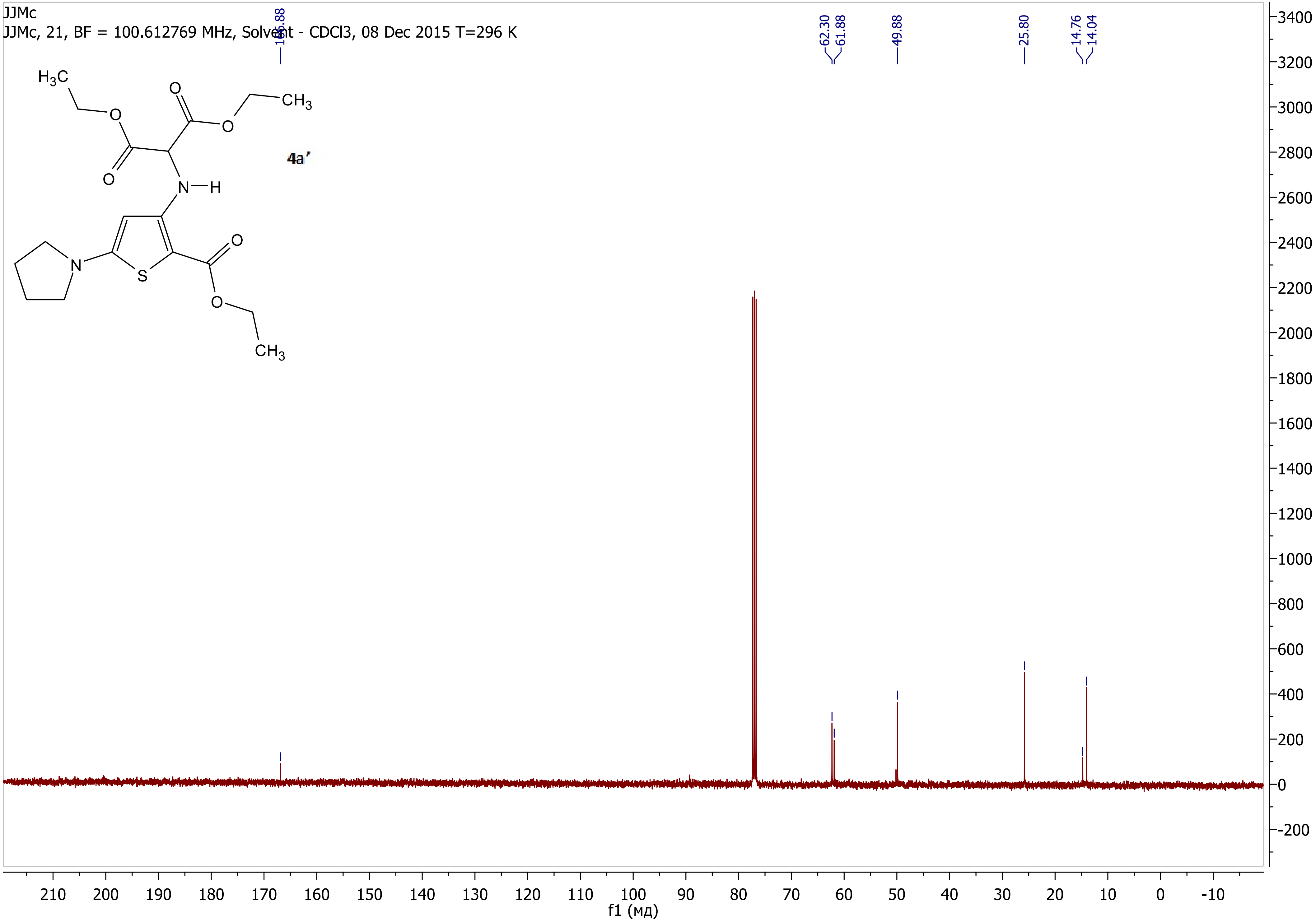

JJMc
JJMc, 21, BF = 100.612769 MHz, Solvent - CDCl3, 08 Dec 2015 T=296 K
166.88
62.30
61.88
49.88
25.80
14.76
14.04
H3C
O
CH3
N—H
N
S
4a’
f1 (мд)

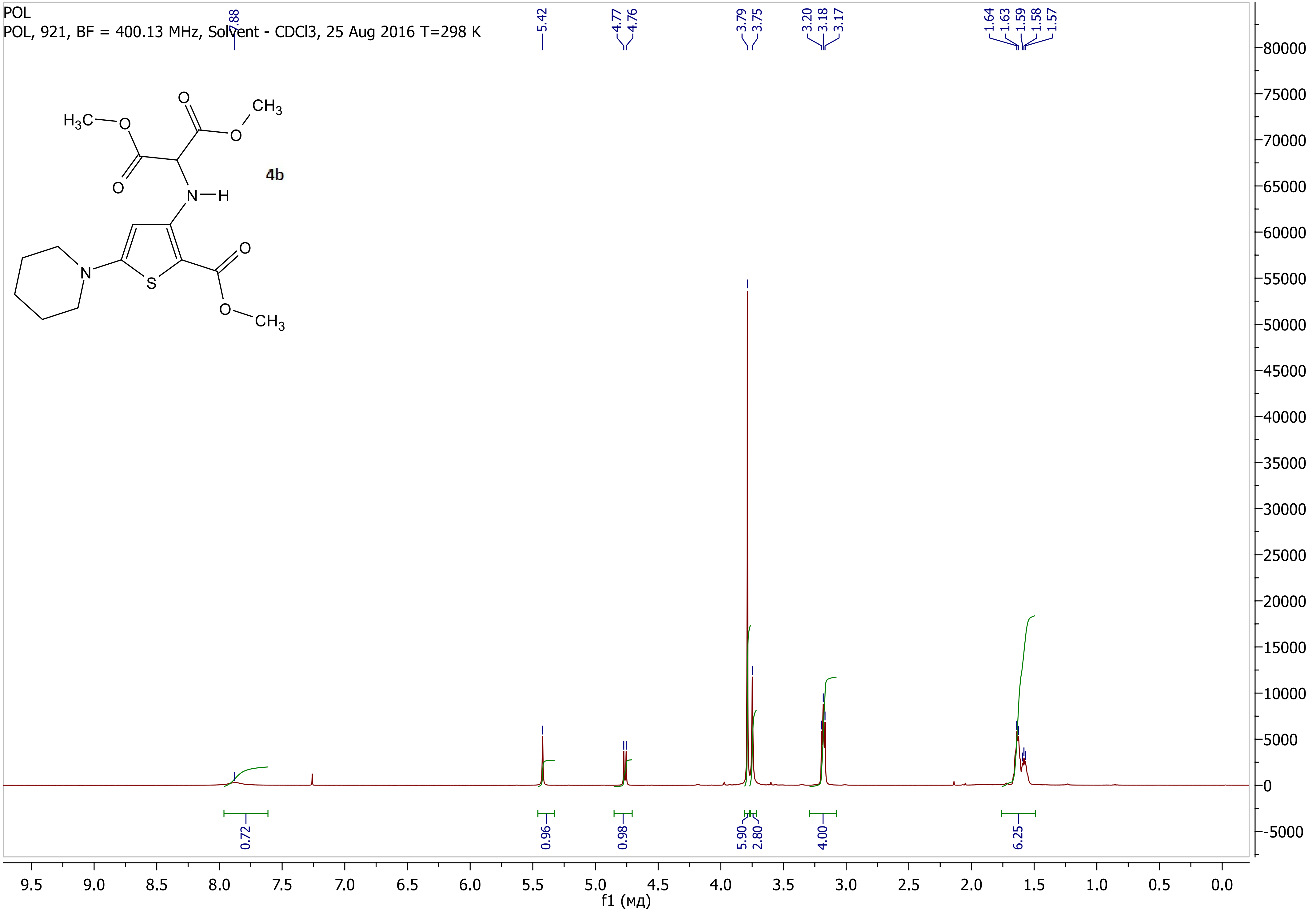
POL
POL, 921, BF = 400.13 MHz, Solvent - CDCl3, 25 Aug 2016 T=298 K
4b
7.88
5.42
4.77
4.76
3.79
3.75
3.20
3.18
3.17
1.64
1.63
1.59
1.58
1.57
0.72
0.96
0.98
5.90
2.80
4.00
6.25
f1 (мд)

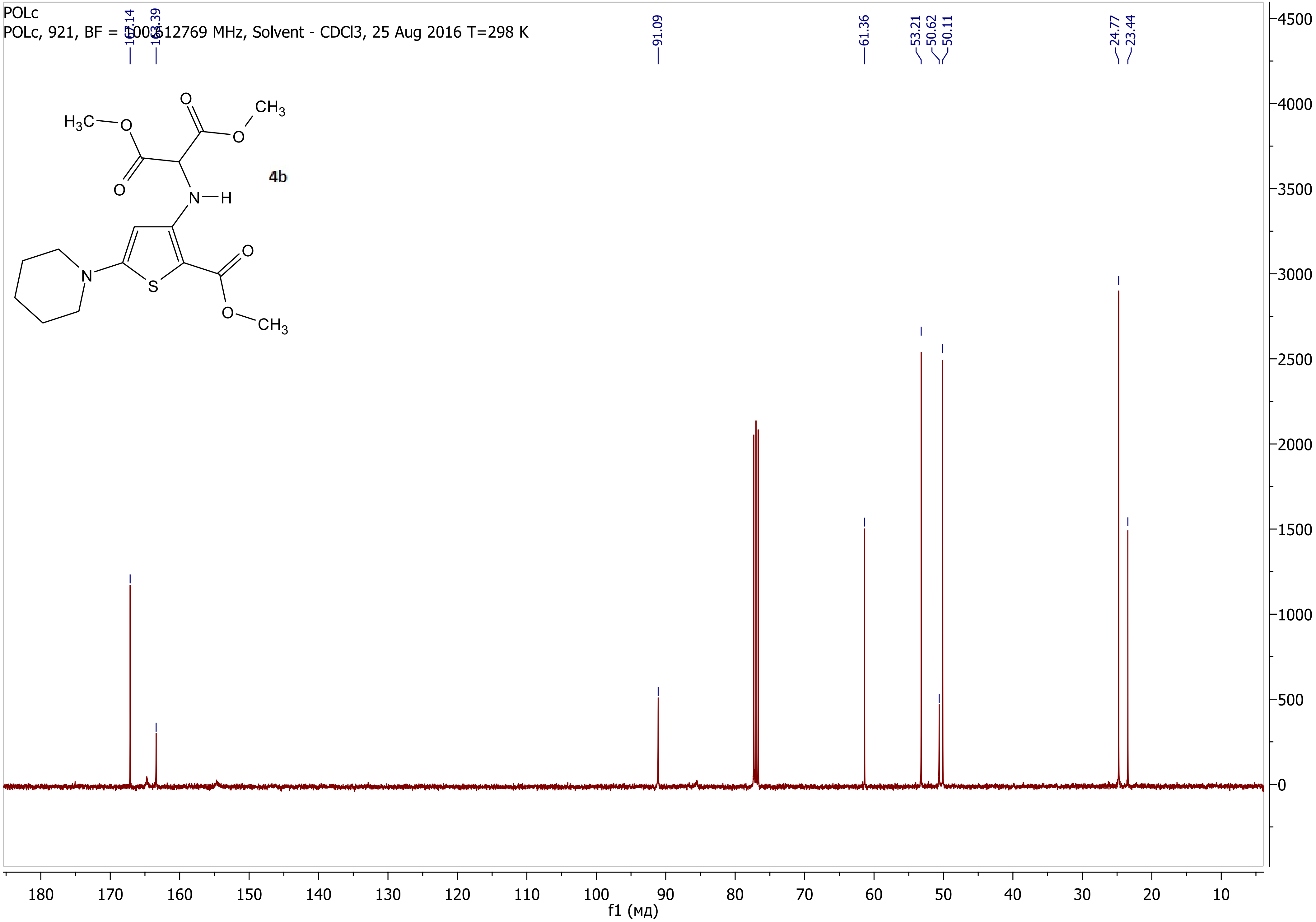
POLc
POLc, 921, BF = 100.612769 MHz, Solvent - CDCl3, 25 Aug 2016 T=298 K
167.14
163.39
91.09
61.36
53.21
50.62
50.11
24.77
23.44
4b
f1 (мд)

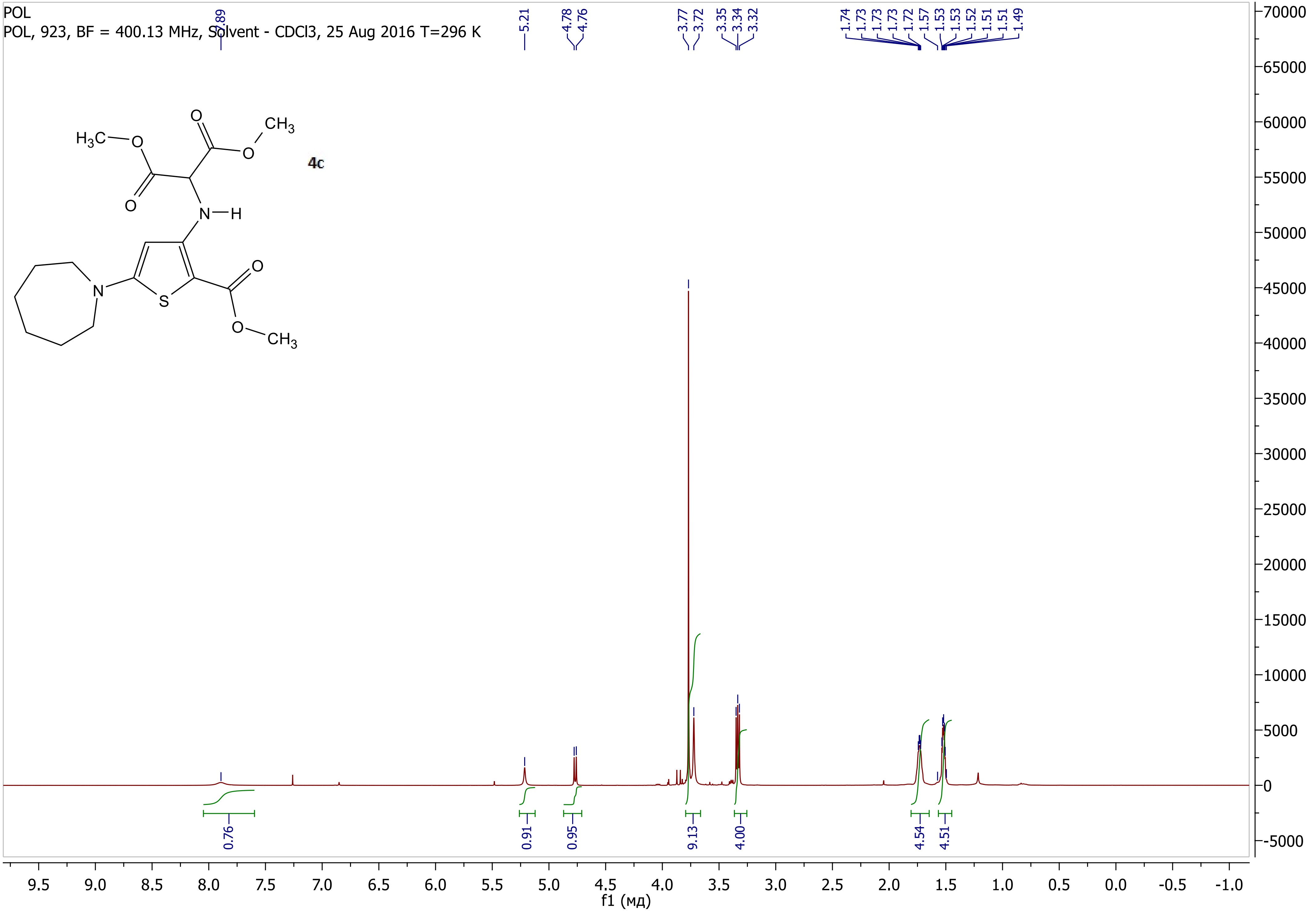
POL
POL, 923, BF = 400.13 MHz, Solvent - CDCl3, 25 Aug 2016 T=296 K
4c
7.89
5.21
4.78
4.76
3.77
3.72
3.35
3.34
3.32
1.74
1.73
1.73
1.73
1.72
1.57
1.53
1.53
1.52
1.51
1.51
1.49
0.76
0.91
0.95
9.13
4.00
4.54
4.51
f1 (мд)

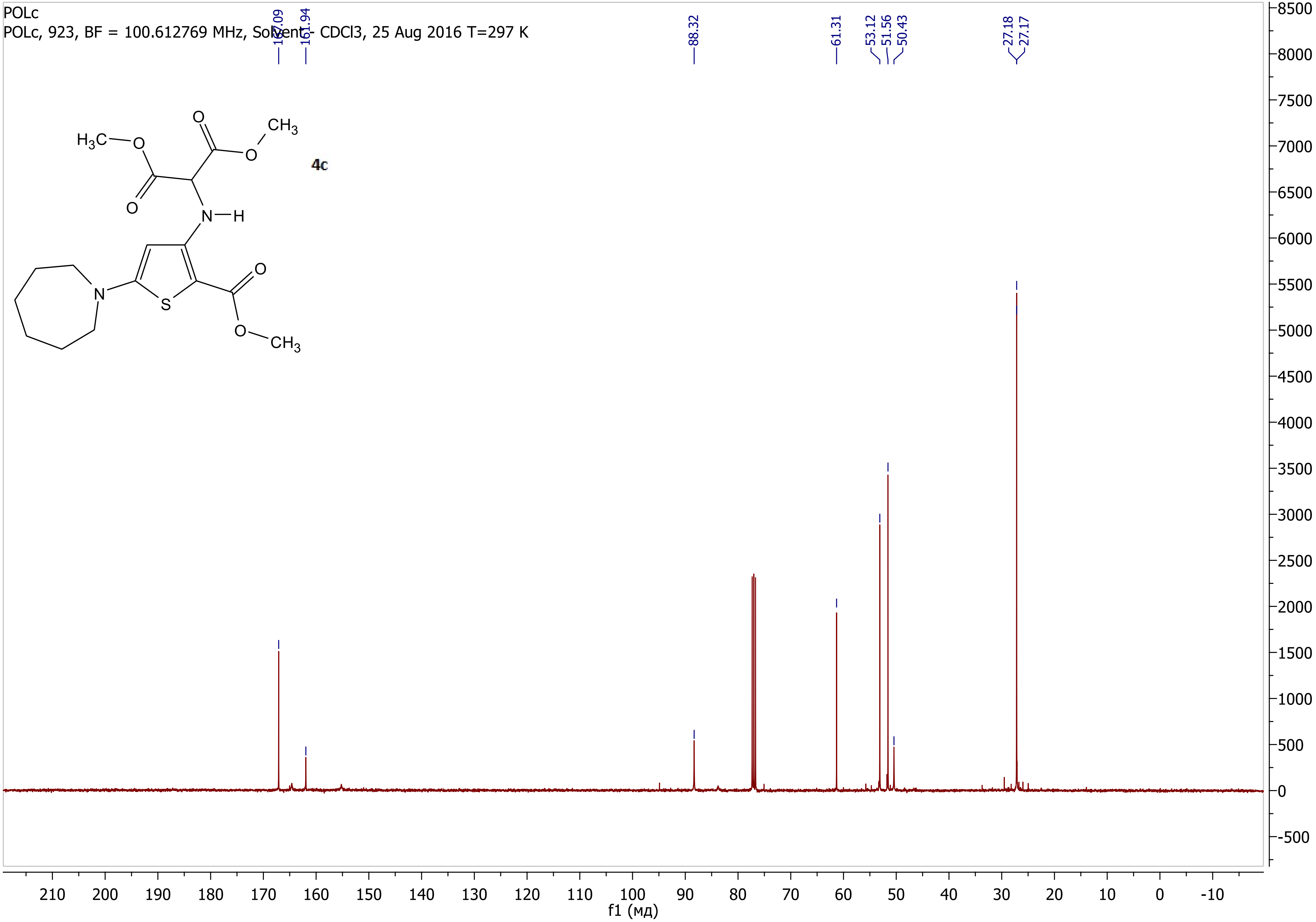
POLc
POLc, 923, BF = 100.612769 MHz, Solvent - CDCl3, 25 Aug 2016 T=297 K
167.09
161.94
88.32
61.31
53.12
51.56
50.43
27.18
27.17
4c
f1 (мд)

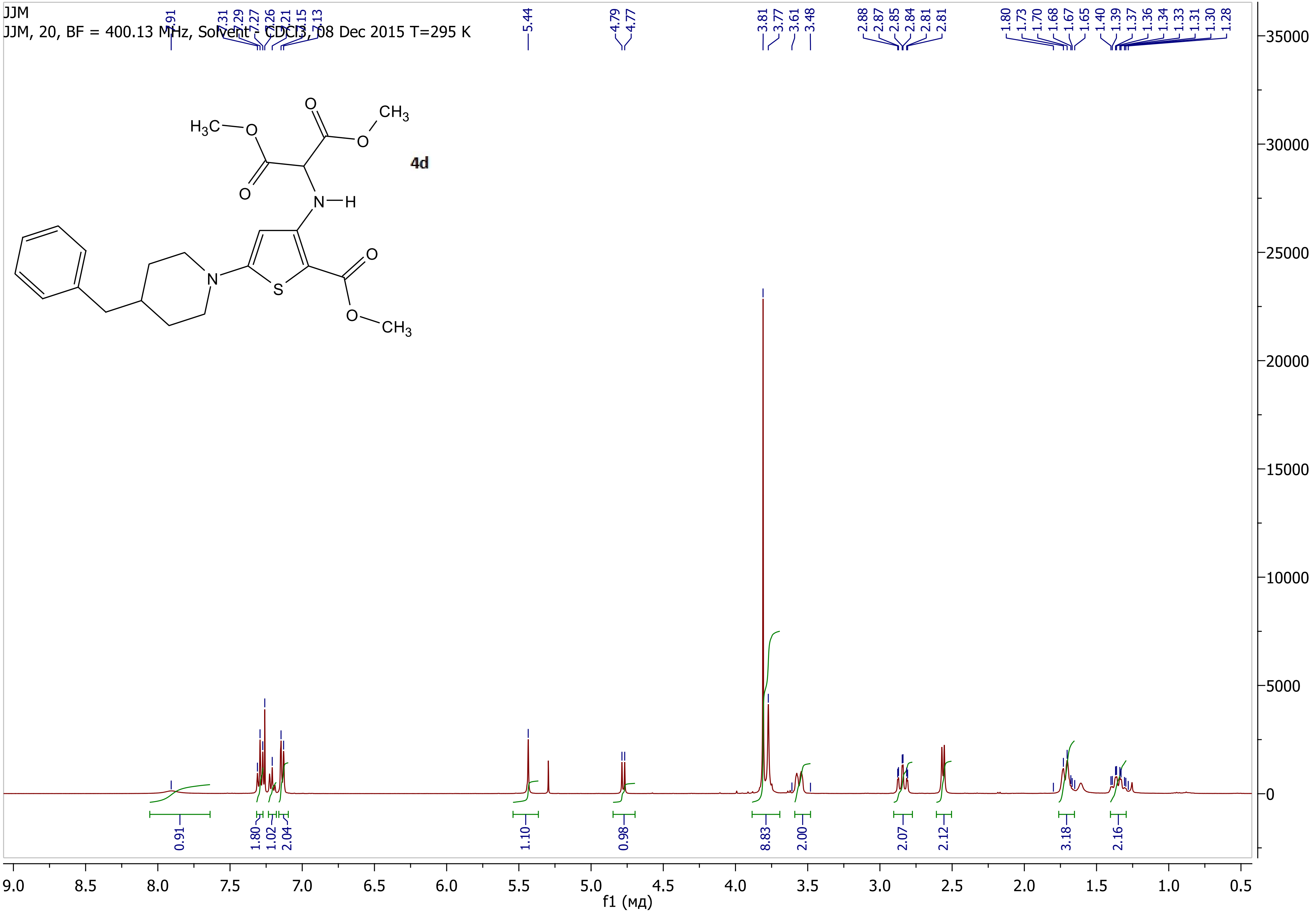
JJM
JJM, 20, BF = 400.13 MHz, Solvent - CDCl3, 08 Dec 2015 T=295 K
7.91
7.31 7.29 7.27 7.26 7.21 7.15 7.13
5.44
4.79 4.77
3.81 3.77 3.61 3.48
2.88 2.87 2.85 2.84 2.81 2.81
1.80 1.73 1.70 1.68 1.67 1.65 1.40 1.39 1.37 1.36 1.34 1.33 1.31 1.30 1.28
H3C O O CH3 O O N H S O O CH3 N
4d
0.91 1.80 1.02 2.04 1.10 0.98 8.83 2.00 2.07 2.12 3.18 2.16
35000 30000 25000 20000 15000 10000 5000 0
9.0 8.5 8.0 7.5 7.0 6.5 6.0 5.5 5.0 4.5 4.0 3.5 3.0 2.5 2.0 1.5 1.0 0.5
f1 (мд)

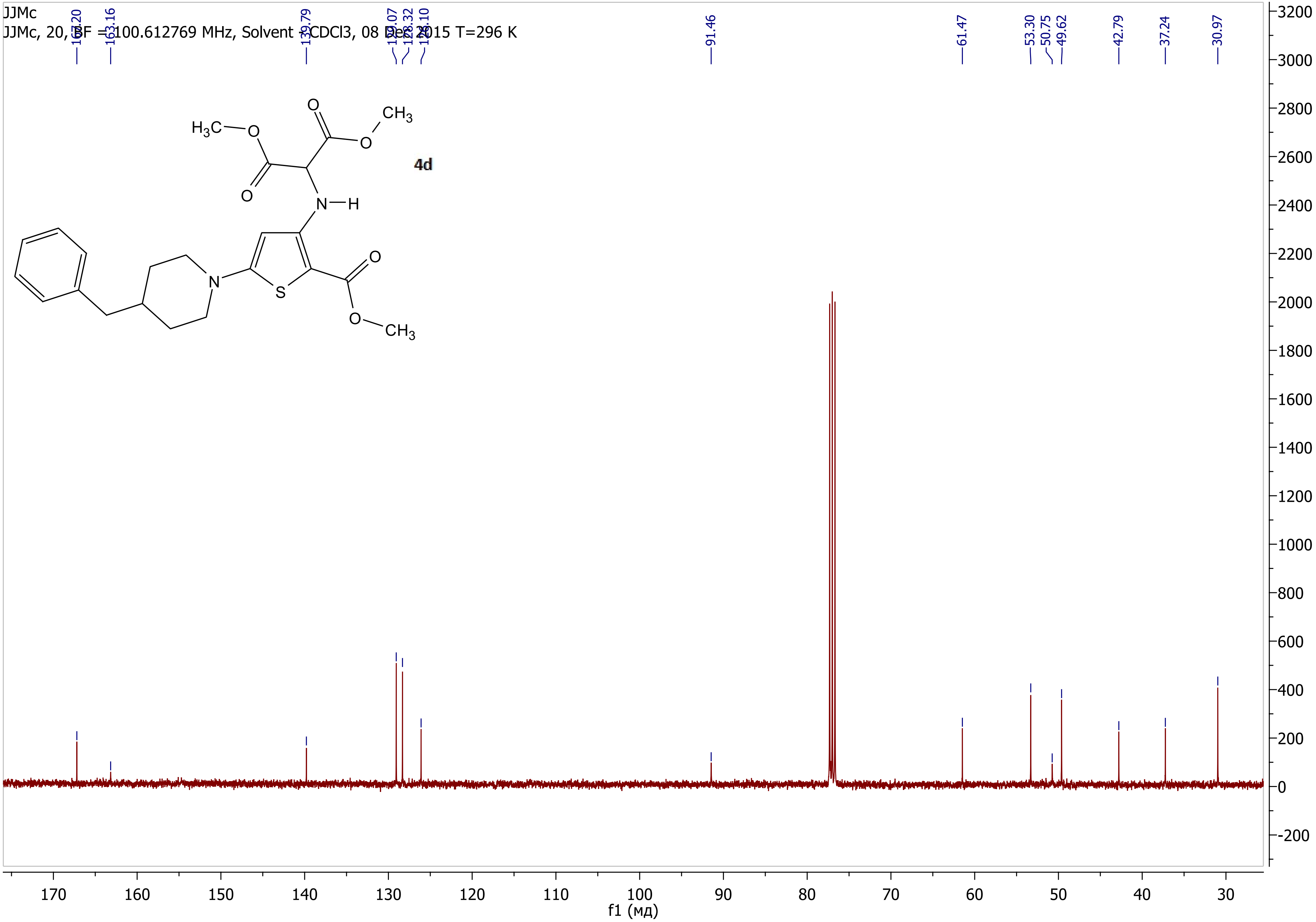
JJMc
JJMc, 20, BF = 100.612769 MHz, Solvent = CDCl3, 08 Dec 2015 T=296 K
163.16
139.79
129.07
128.32
126.10
91.46
61.47
53.30
50.75
49.62
42.79
37.24
30.97
4d
f1 (мд)

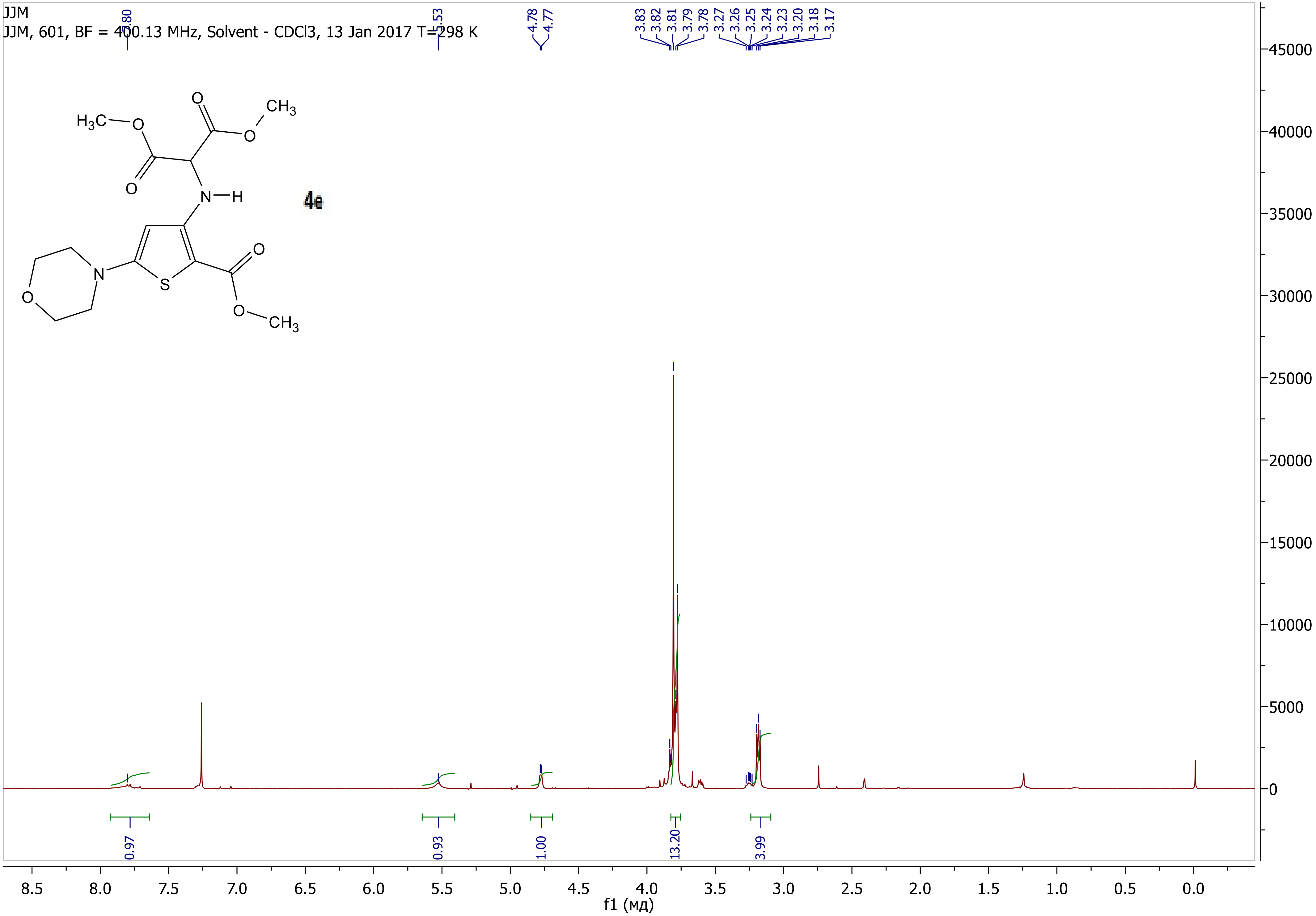
JJM
JJM, 601, BF = 400.13 MHz, Solvent - CDCl3, 13 Jan 2017 T=298 K
7.80
5.53
4.78
4.77
3.83
3.82
3.81
3.79
3.78
3.27
3.26
3.25
3.24
3.23
3.20
3.18
3.17
4e
0.97
0.93
1.00
13.20
3.99
f1 (мд)

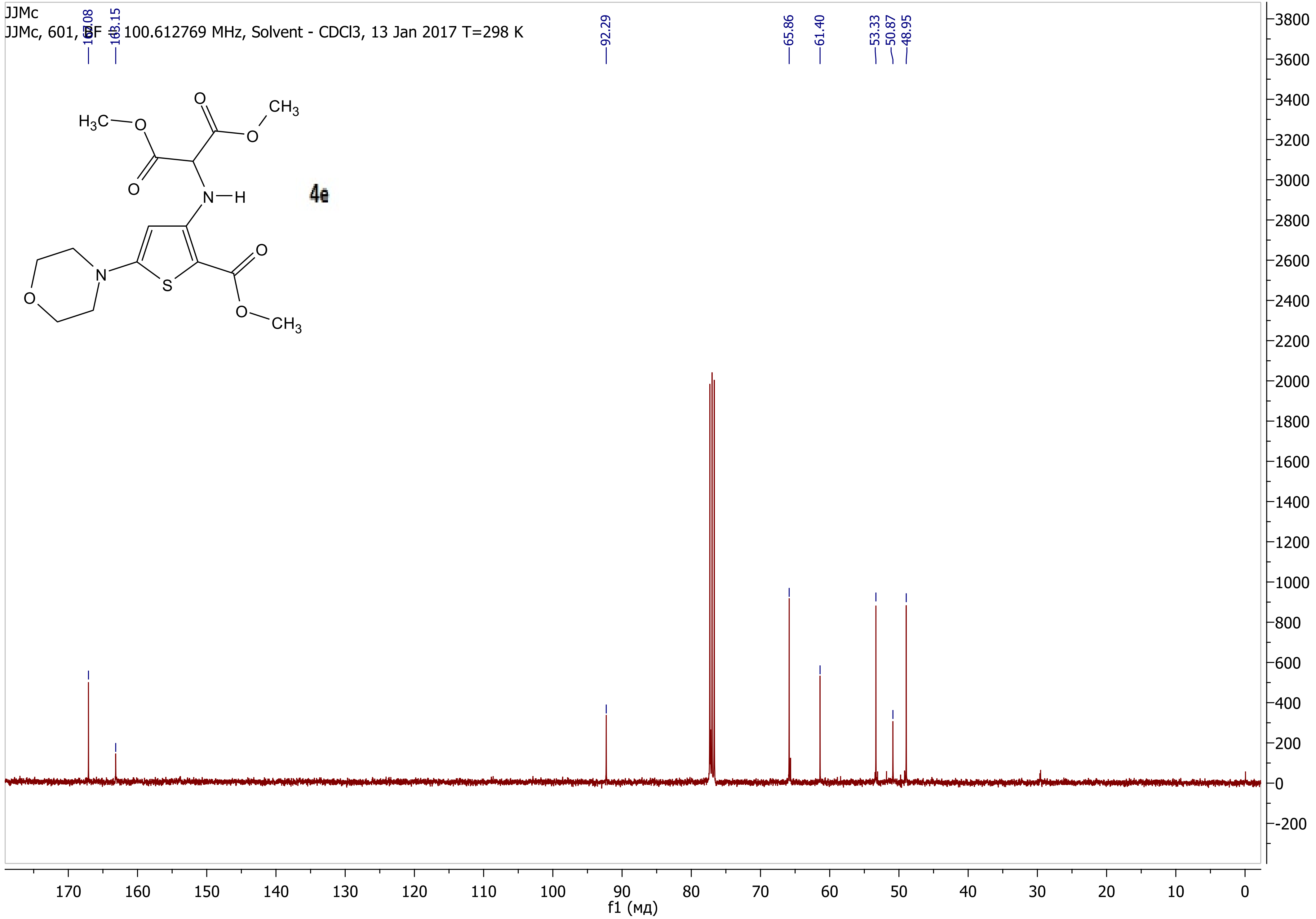
JJMc
JJMc, 601, BF = 100.612769 MHz, Solvent - CDCl3, 13 Jan 2017 T=298 K
167.08
163.15
92.29
65.86
61.40
53.33
50.87
48.95
4e
f1 (мд)

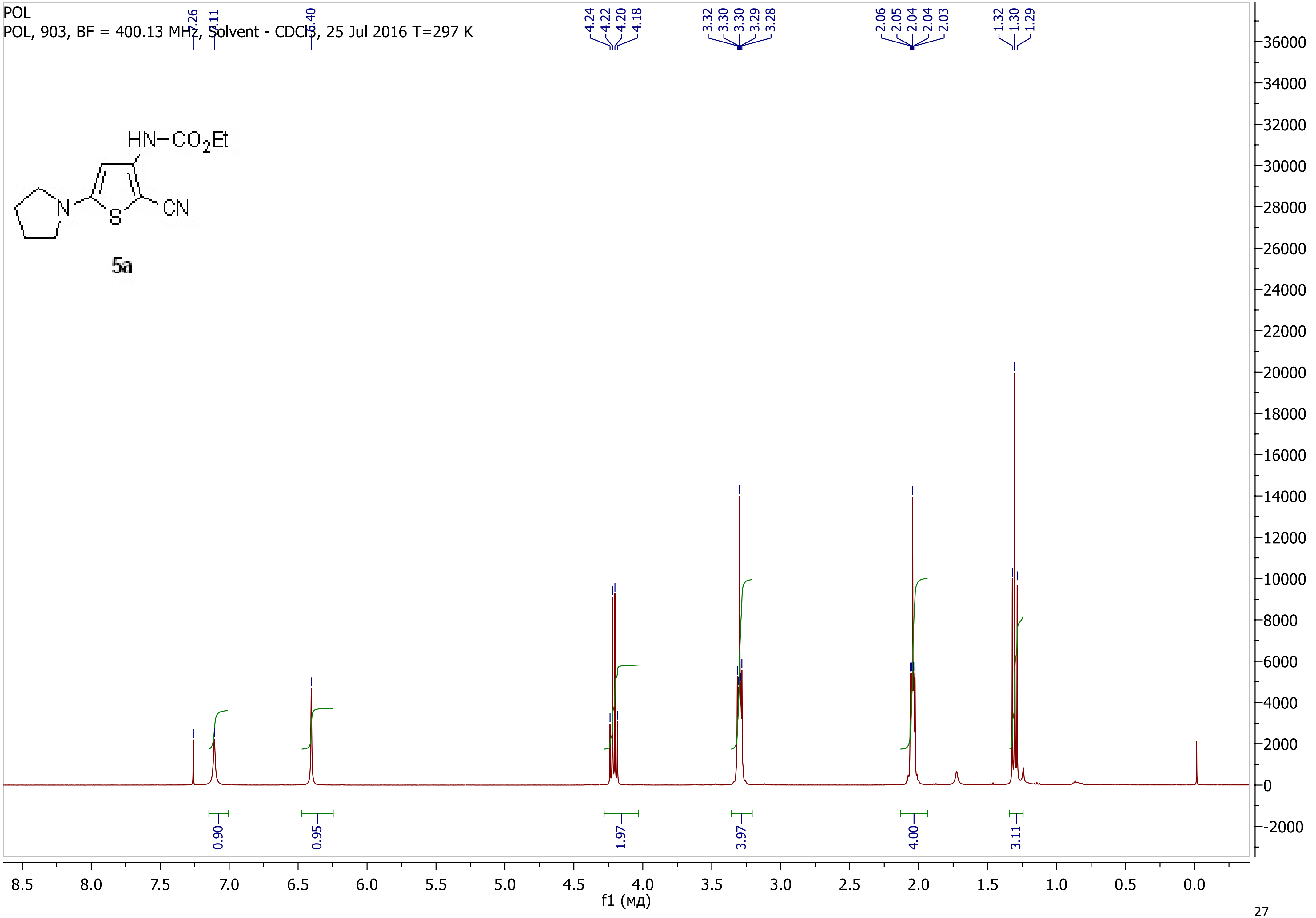
POL
POL, 903, BF = 400.13 MHz, Solvent - CDCl3, 25 Jul 2016 T=297 K
7.26
7.11
6.40
4.24
4.22
4.20
4.18
3.32
3.30
3.30
3.29
3.28
2.06
2.05
2.04
2.04
2.03
1.32
1.30
1.29
HN-CO2Et
CN
N
S
5a
0.90
0.95
1.97
3.97
4.00
3.11
f1 (мд)

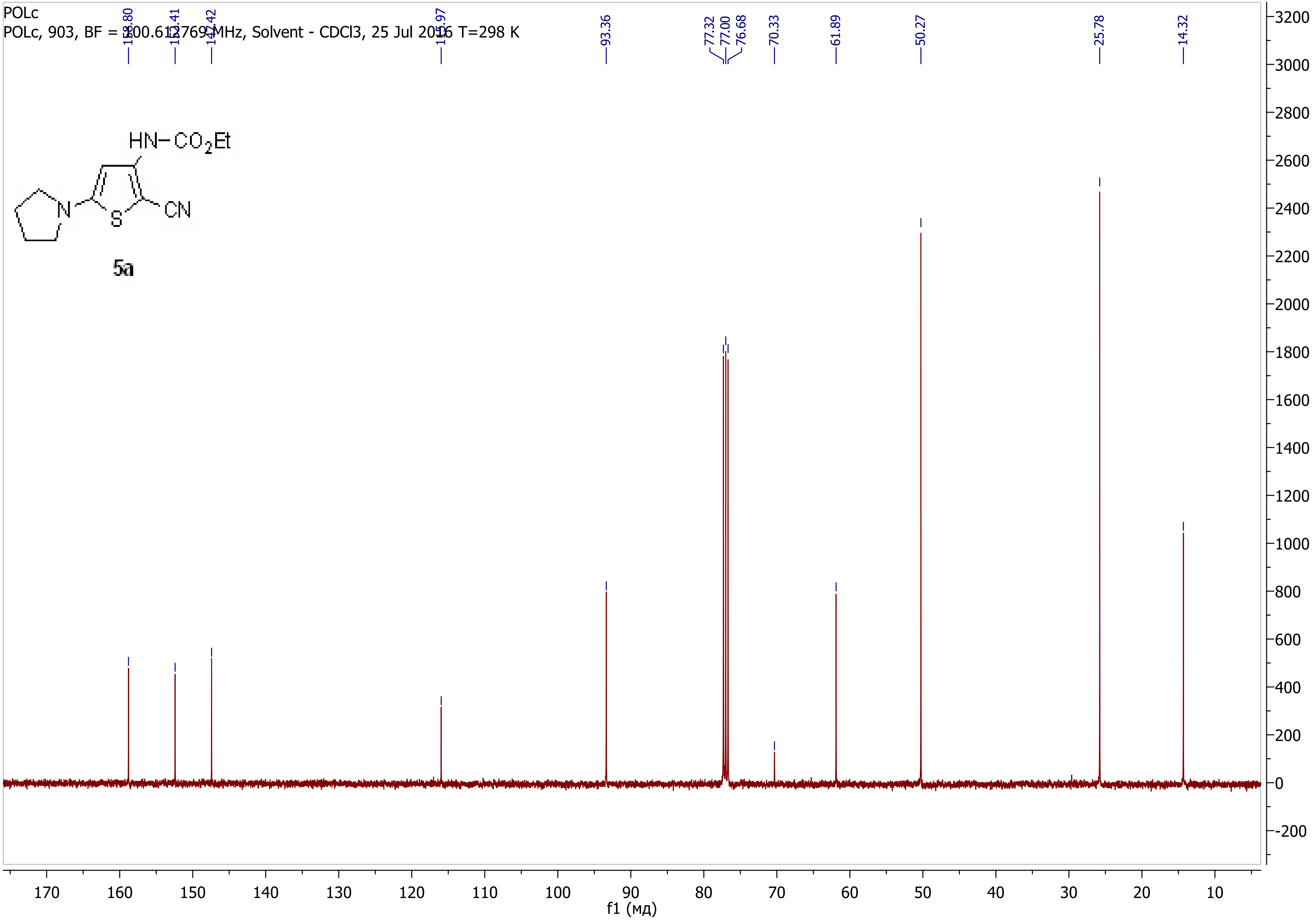

POLc
POLc, 903, BF = 100.612769 MHz, Solvent - CDCl3, 25 Jul 2016 T=298 K
158.80
152.41
147.42
115.97
93.36
77.32
77.00
76.68
70.33
61.89
50.27
25.78
14.32
HN−CO2Et
N
S
CN
5a
f1 (мд)

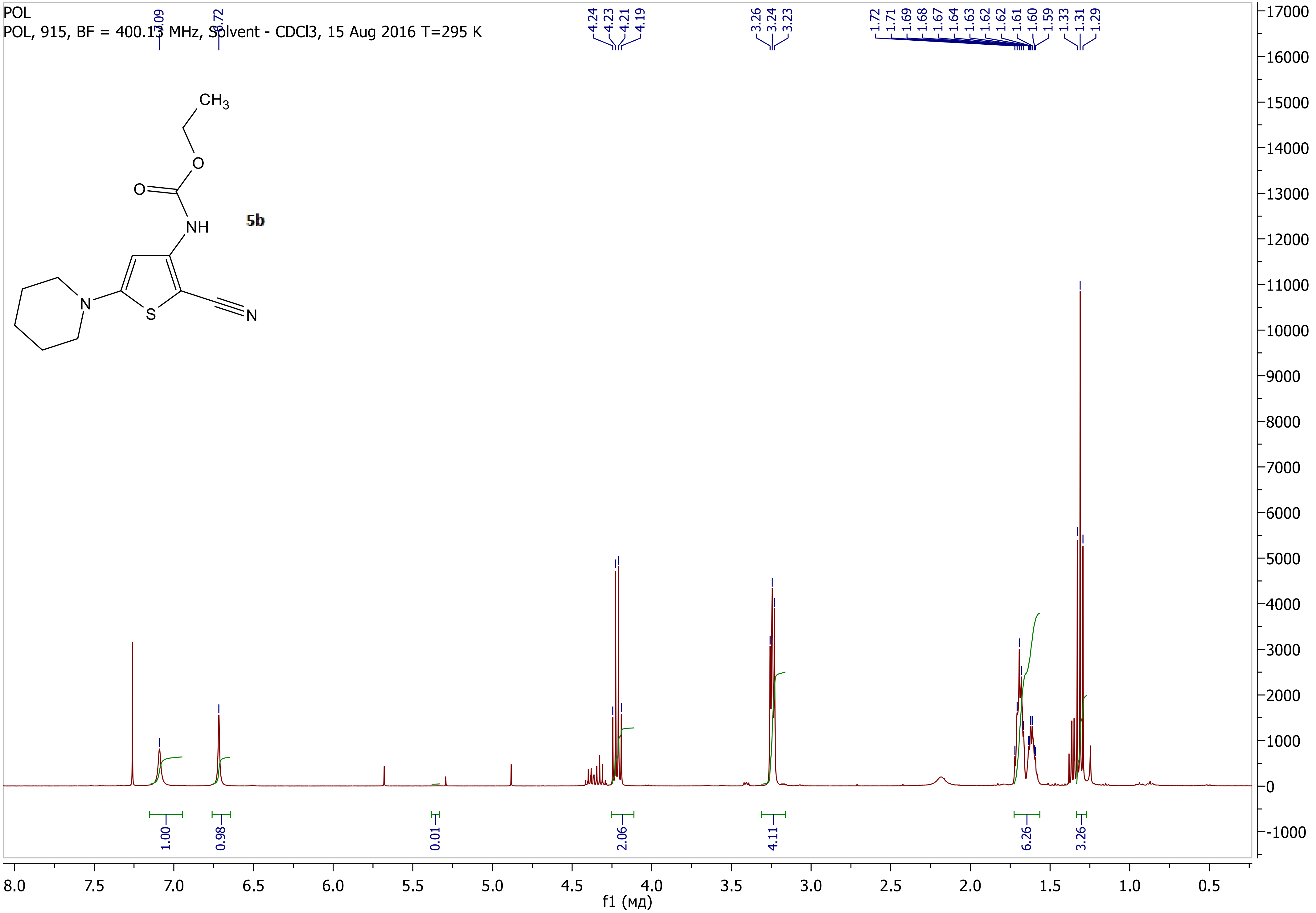
POL
POL, 915, BF = 400.13 MHz, Solvent - CDCl3, 15 Aug 2016 T=295 K
7.09
6.72
4.24
4.23
4.21
4.19
3.26
3.24
3.23
1.72
1.71
1.69
1.68
1.67
1.64
1.63
1.62
1.62
1.61
1.60
1.59
1.33
1.31
1.29
CH3
O
O
NH
N
S
N
5b
1.00
0.98
0.01
2.06
4.11
6.26
3.26
f1 (мд)

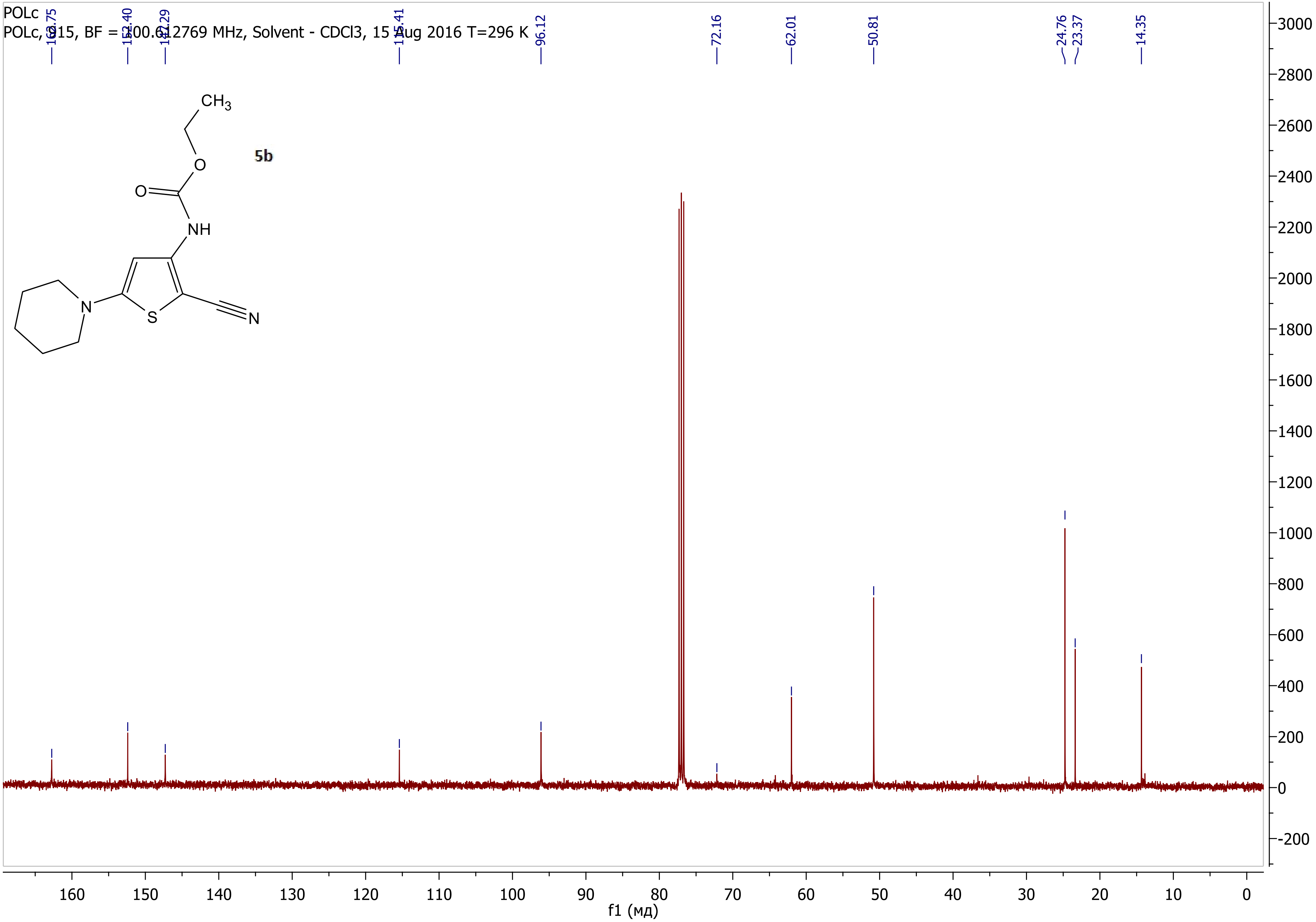
POLc
POLc, 915, BF = 100.612769 MHz, Solvent - CDCl3, 15 Aug 2016 T=296 K
162.75
152.40
147.29
115.41
96.12
72.16
62.01
50.81
24.76
23.37
14.35
CH3
O
O
NH
N
S
N
5b
f1 (мд)

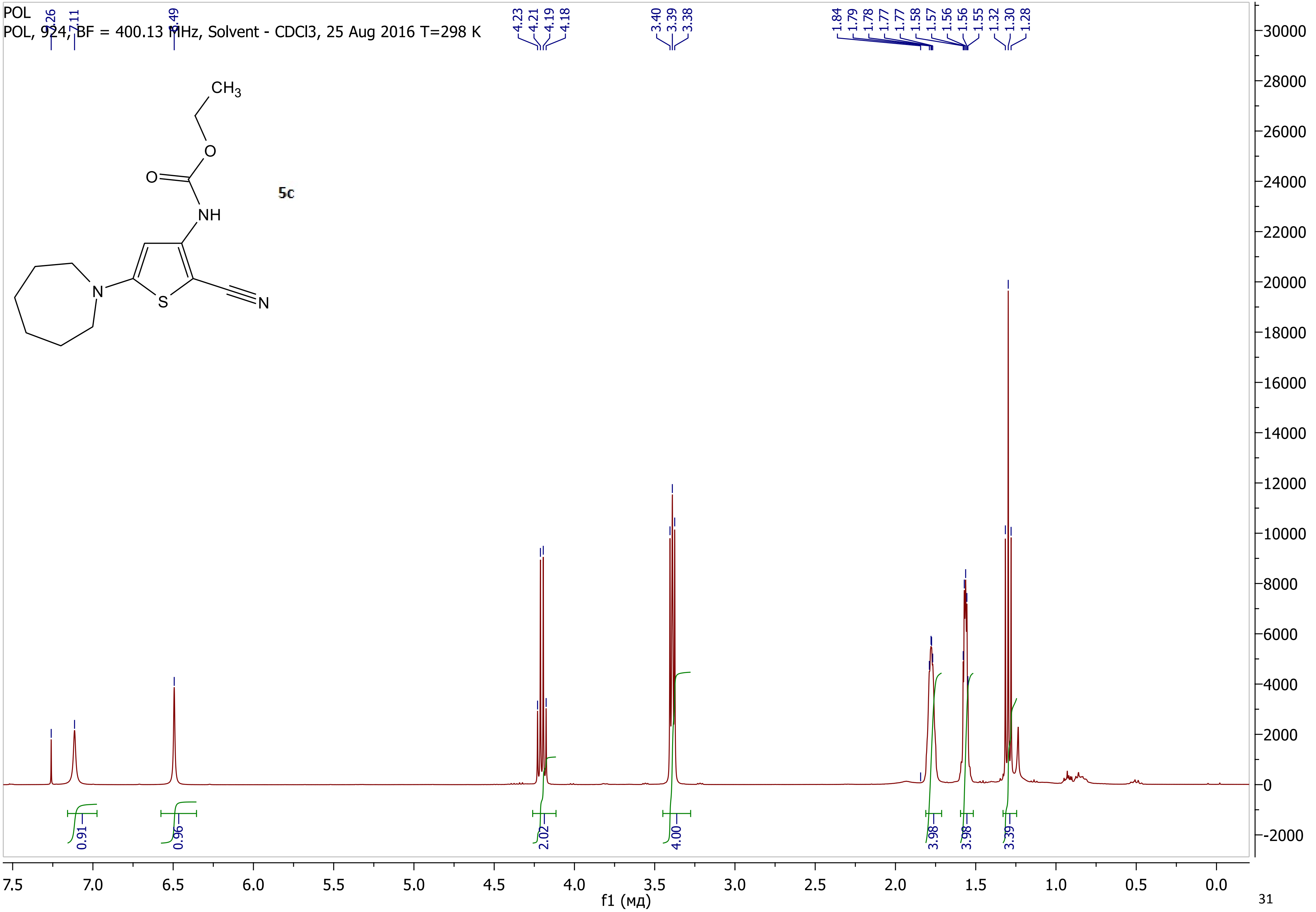
POL
POL, 924, BF = 400.13 MHz, Solvent - CDCl3, 25 Aug 2016 T=298 K
7.26
7.11
6.49
4.23
4.21
4.19
4.18
3.40
3.39
3.38
1.84
1.79
1.78
1.77
1.77
1.58
1.57
1.56
1.56
1.55
1.32
1.30
1.28
CH3
O
O
NH
N
S
N
5c
0.91
0.96
2.02
4.00
3.98
3.98
3.39
f1 (мд)

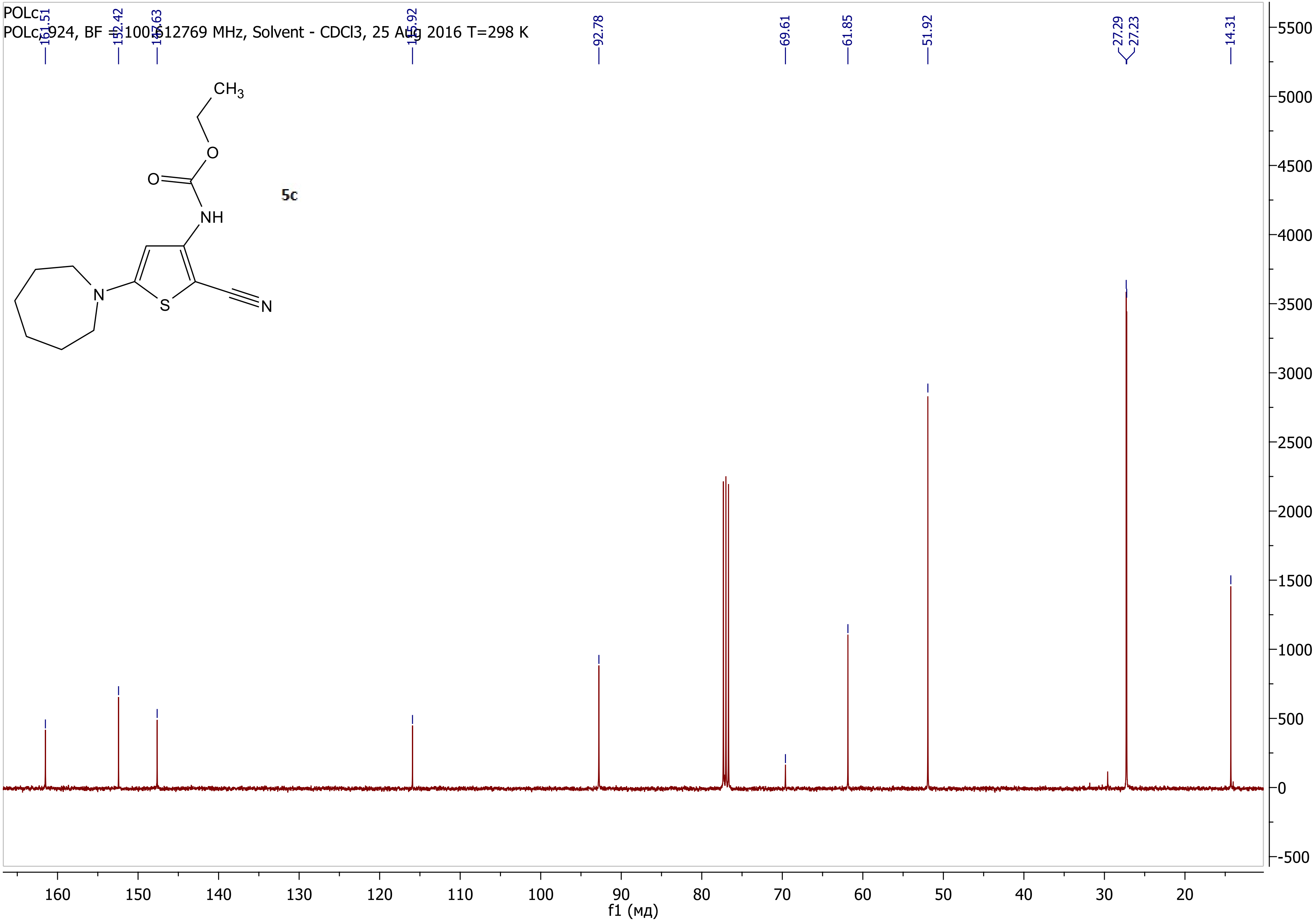
POLc
POLc.924, BF = 100.612769 MHz, Solvent - CDCl3, 25 Aug 2016 T=298 K
92.78
69.61
61.85
51.92
27.29
27.23
14.31
CH3
O
O
NH
N
S
N
5c
f1 (мд)

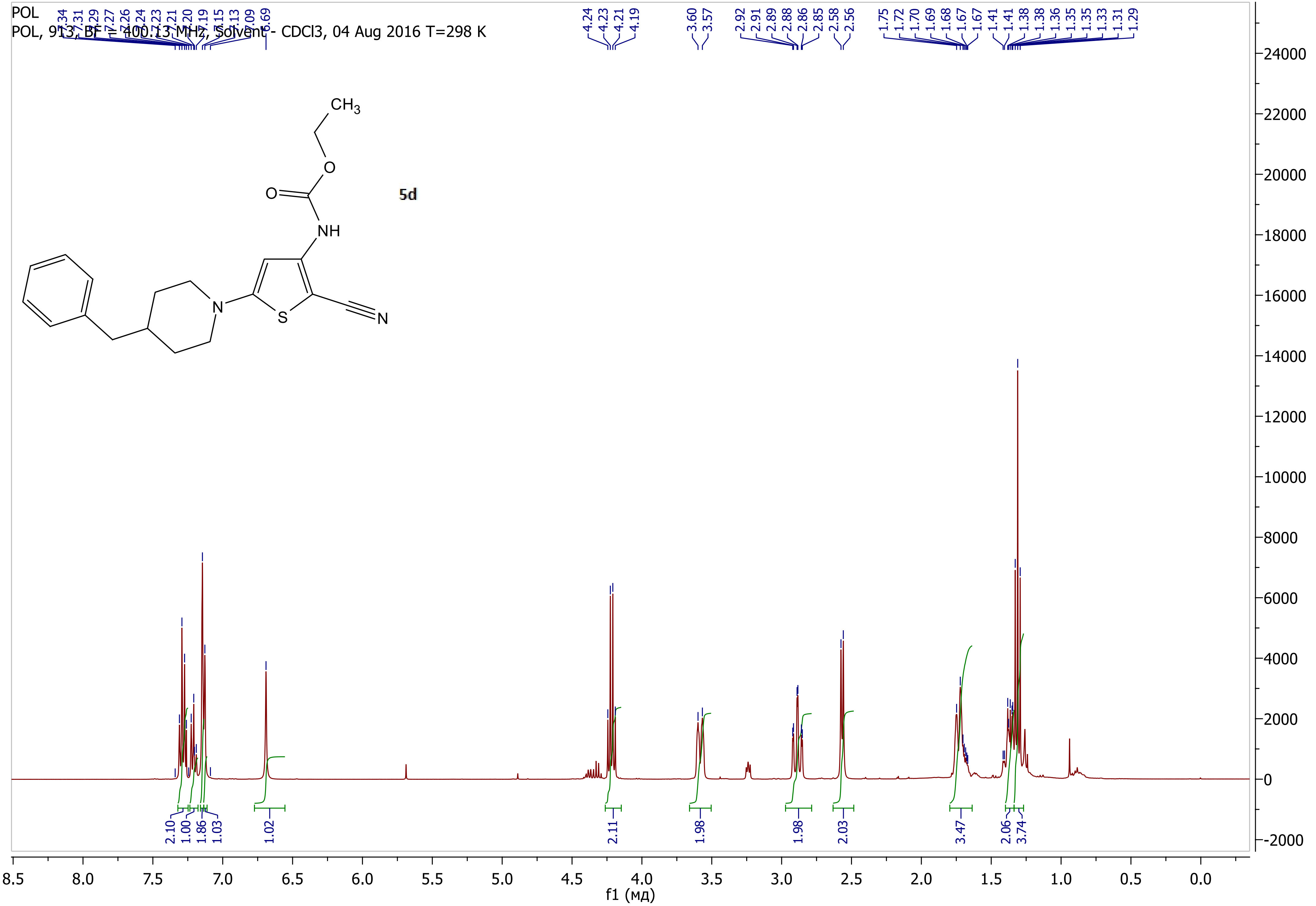
POL
POL, 913, BF = 400.13 MHz, Solvent - CDCl3, 04 Aug 2016 T=298 K
CH3
O
O
NH
N
S
N
5d
f1 (мд)

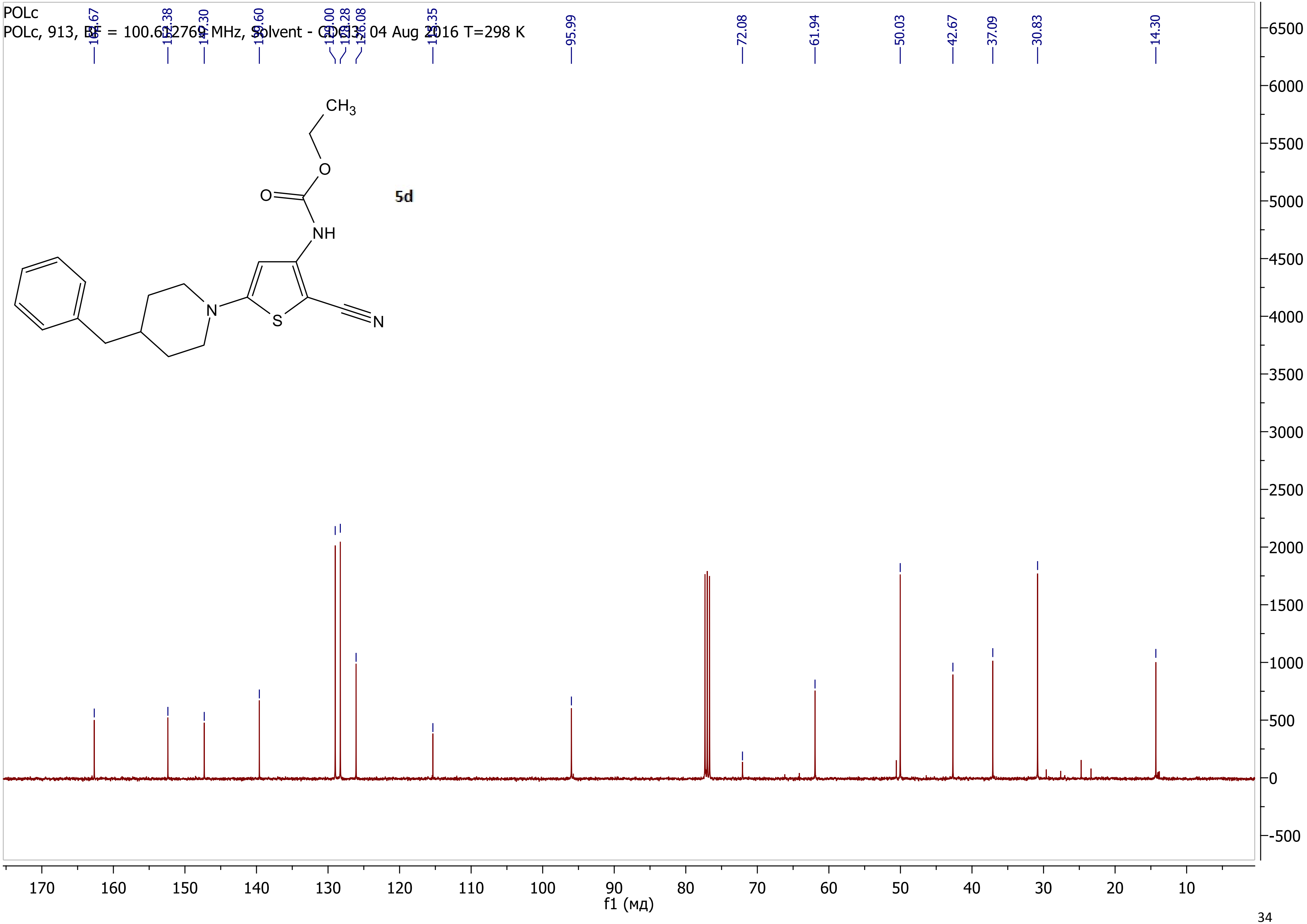
POLc
POLc, 913, BF = 100.612769 MHz, Solvent - CDCl3, 04 Aug 2016 T=298 K
95.99
72.08
61.94
50.03
42.67
37.09
30.83
14.30
5d
f1 (мд)

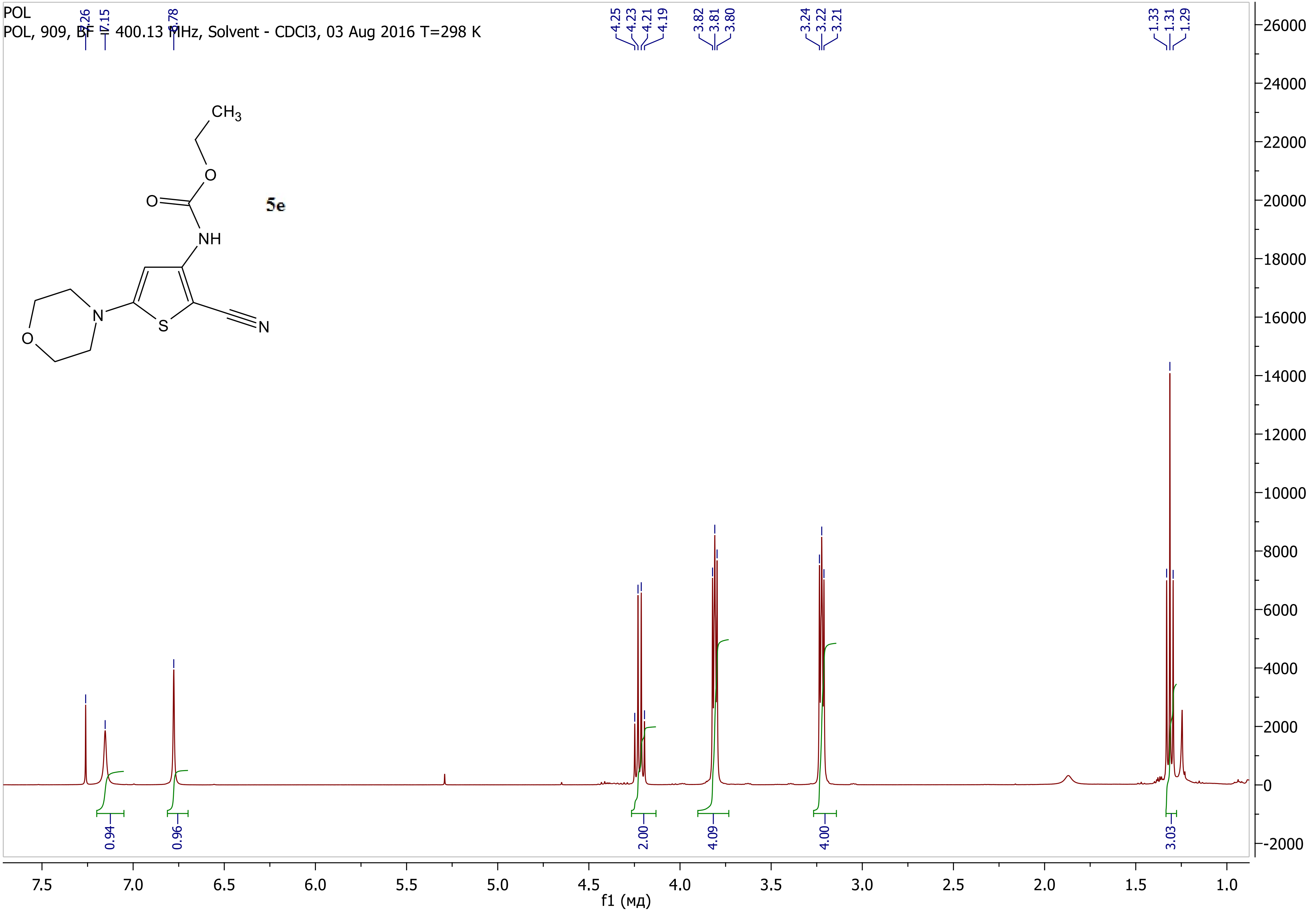
POL
POL, 909, BF = 400.13 MHz, Solvent - CDCl3, 03 Aug 2016 T=298 K
7.26
7.15
6.78
4.25
4.23
4.21
4.19
3.82
3.81
3.80
3.24
3.22
3.21
1.33
1.31
1.29
5e
CH3
O
O
NH
N
S
N
O
0.94
0.96
2.00
4.09
4.00
3.03
f1 (мд)

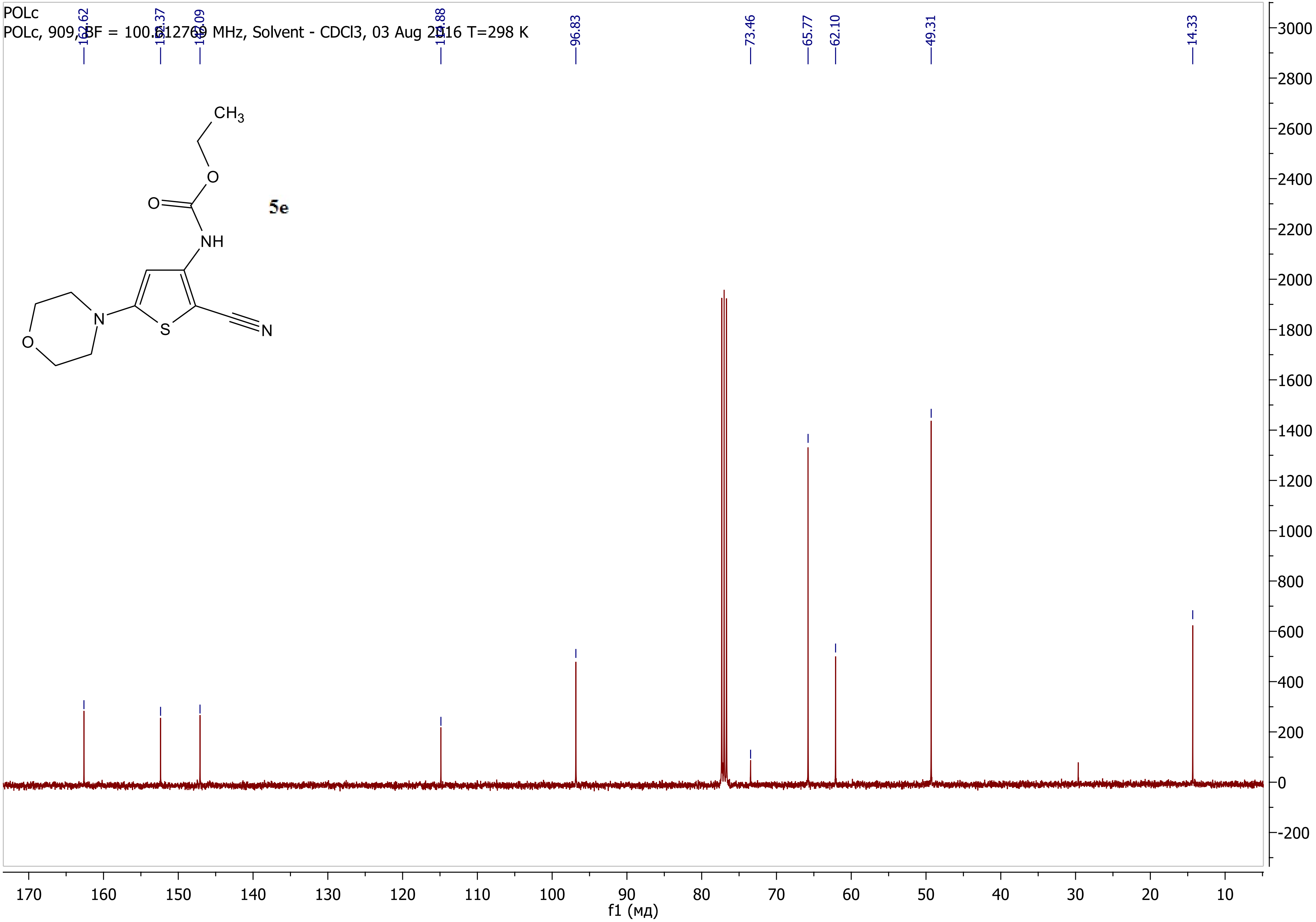
POLc
POLc, 909, BF = 100.612769 MHz, Solvent - CDCl3, 03 Aug 2016 T=298 K
162.62
96.83
73.46
65.77
62.10
49.31
14.33
CH3
O
O
NH
N
S
N
O
5e
f1 (мд)

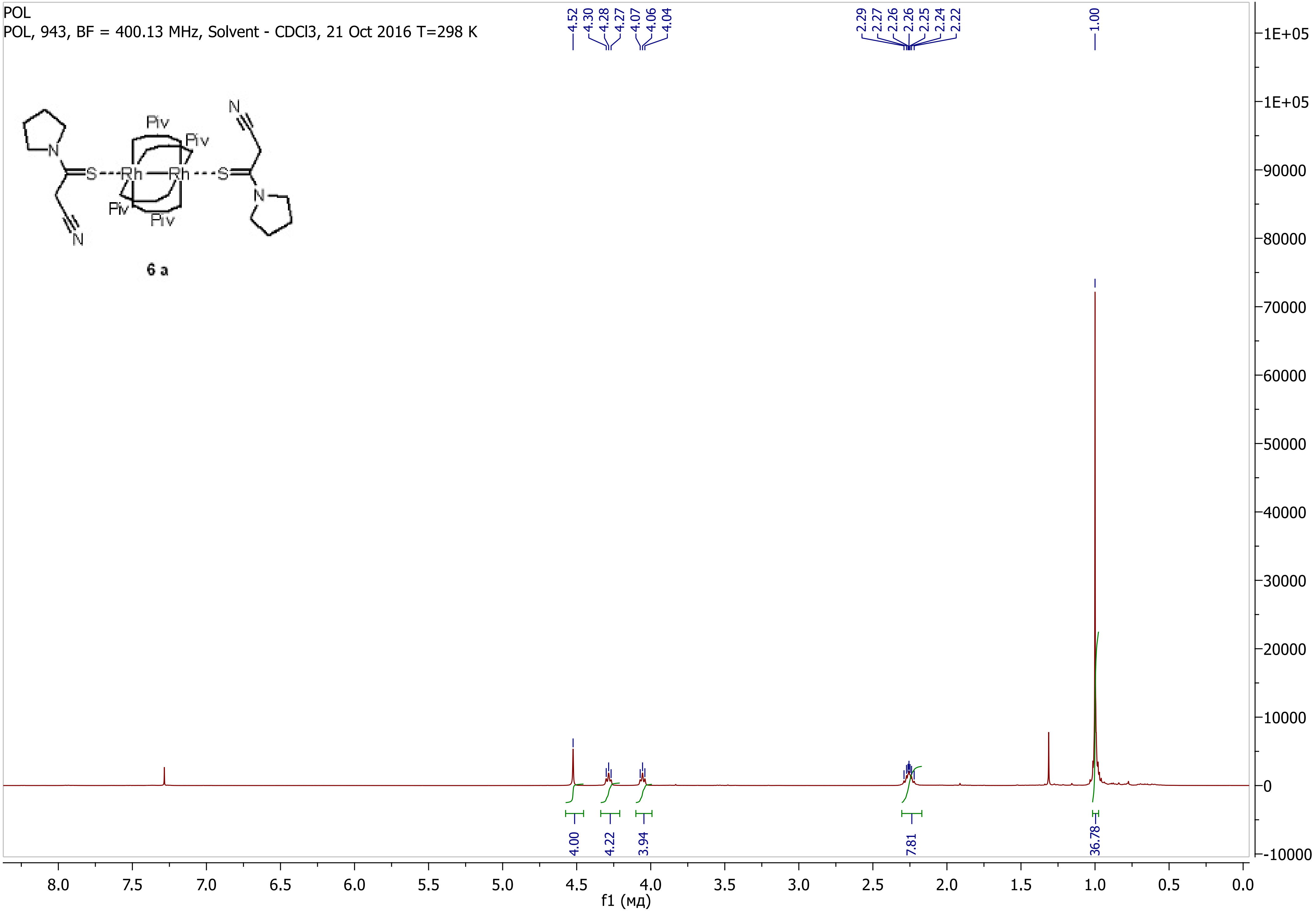
POL
POL, 943, BF = 400.13 MHz, Solvent - CDCl3, 21 Oct 2016 T=298 K
4.52
4.30
4.28
4.27
4.07
4.06
4.04
2.29
2.27
2.26
2.26
2.25
2.24
2.22
1.00
Piv
Piv
Piv
Piv
N
S
Rh
Rh
S
N
N
N
6 a
4.00
4.22
3.94
7.81
36.78
f1 (мд)
8.0 7.5 7.0 6.5 6.0 5.5 5.0 4.5 4.0 3.5 3.0 2.5 2.0 1.5 1.0 0.5 0.0
1E+05
1E+05
90000
80000
70000
60000
50000
40000
30000
20000
10000
0
-10000

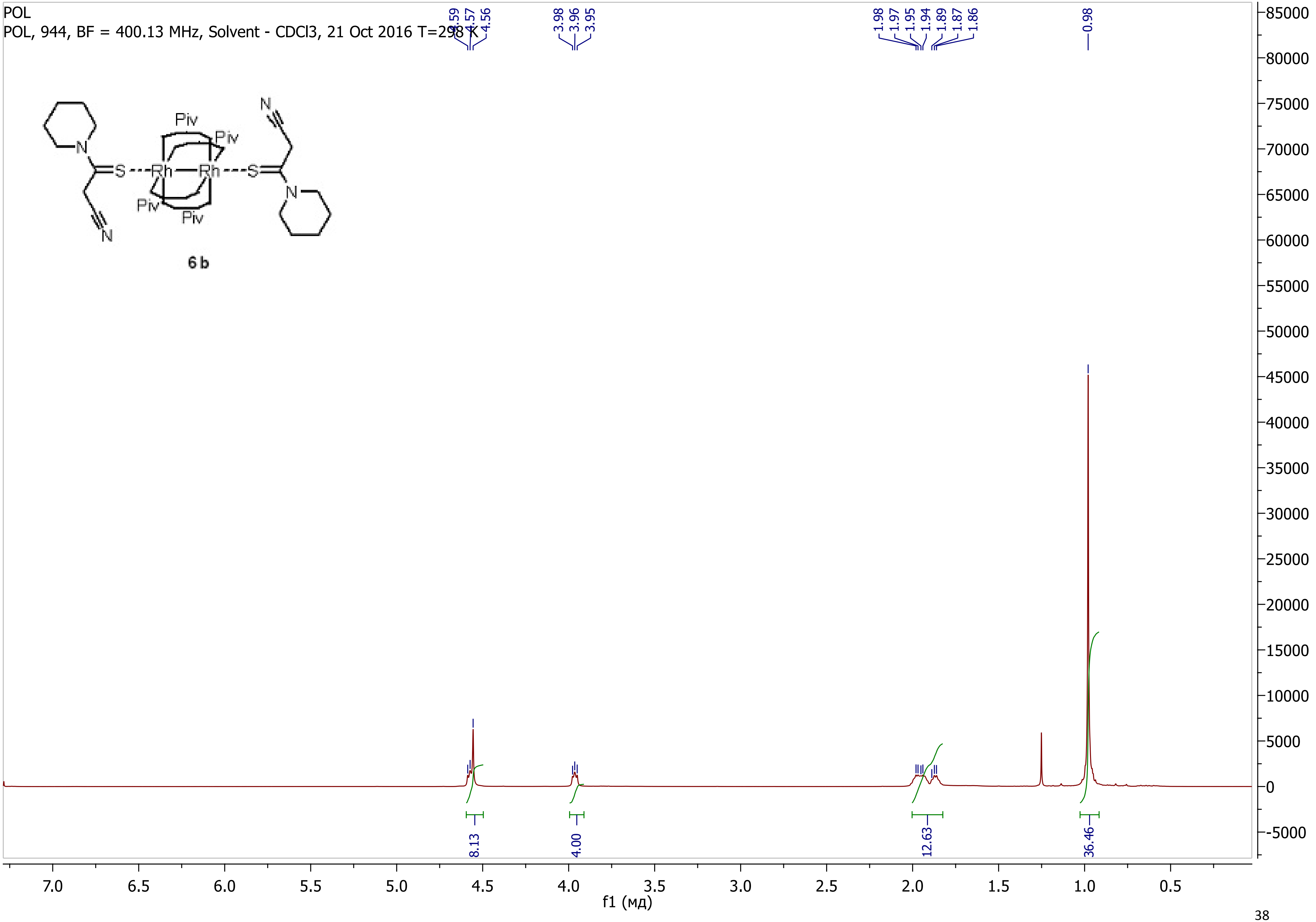
POL
POL, 944, BF = 400.13 MHz, Solvent - CDCl3, 21 Oct 2016 T=298 K
4.59
4.57
4.56
3.98
3.96
3.95
1.98
1.97
1.95
1.94
1.89
1.87
1.86
0.98
Piv
Piv
Piv
Piv
Rh
Rh
S
S
N
N
N
N
6b
8.13
4.00
12.63
36.46
85000
80000
75000
70000
65000
60000
55000
50000
45000
40000
35000
30000
25000
20000
15000
10000
5000
0
-5000
7.0
6.5
6.0
5.5
5.0
4.5
4.0
3.5
3.0
2.5
2.0
1.5
1.0
0.5
f1 (мд)

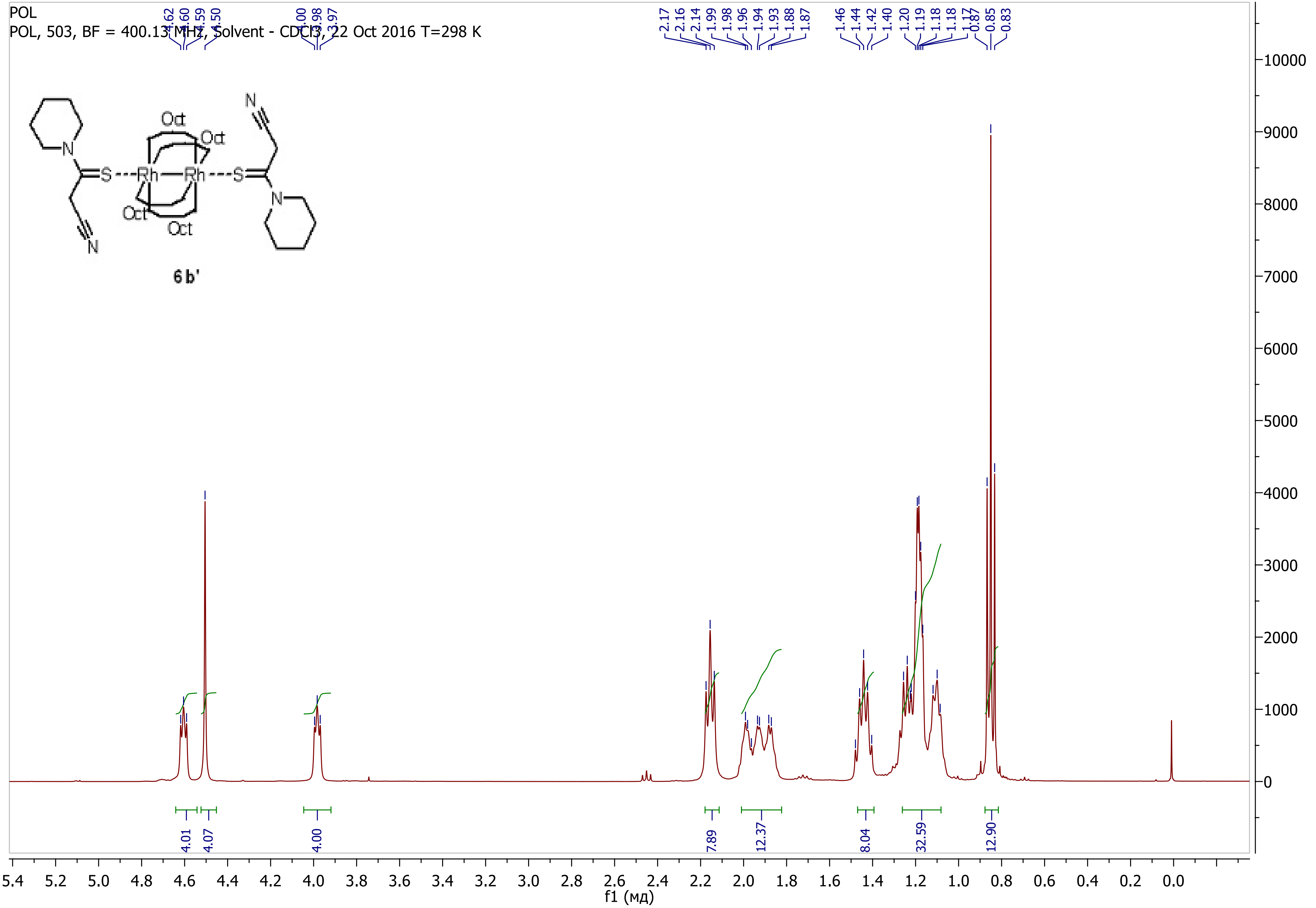
POL
POL, 503, BF = 400.13 MHz, Solvent - CDCl3, 22 Oct 2016 T=298 K
4.62 4.60 4.59 4.50 4.00 3.98 3.97
2.17 2.16 2.14 1.99 1.98 1.96 1.94 1.93 1.88 1.87
1.46 1.44 1.42 1.40 1.20 1.19 1.18 1.18 1.17 0.87 0.85 0.83
Oct
Oct
Oct
Oct
N
N
S
S
Rh
Rh
6 b'
4.01 4.07 4.00 7.89 12.37 8.04 32.59 12.90
f1 (мд)

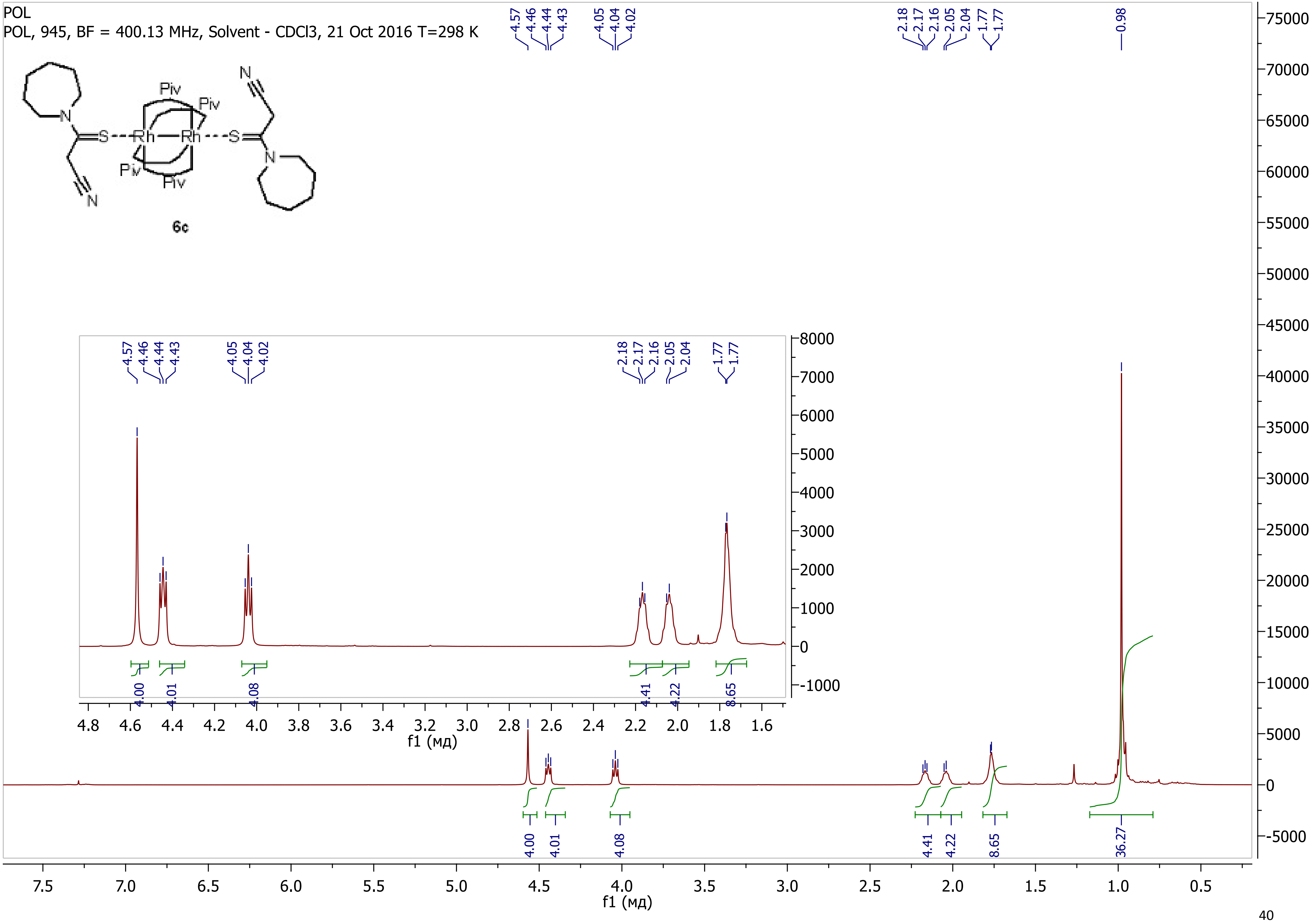
POL
POL, 945, BF = 400.13 MHz, Solvent - CDCl3, 21 Oct 2016 T=298 K
4.57 4.46 4.44 4.43 4.05 4.04 4.02 2.18 2.17 2.16 2.05 2.04 1.77 1.77 0.98
Piv
Piv
Piv
Piv
N
N
N
N
S
S
Rh
Rh
6c
4.00 4.01 4.08 4.41 4.22 8.65 36.27
f1 (мд)
f1 (мд)

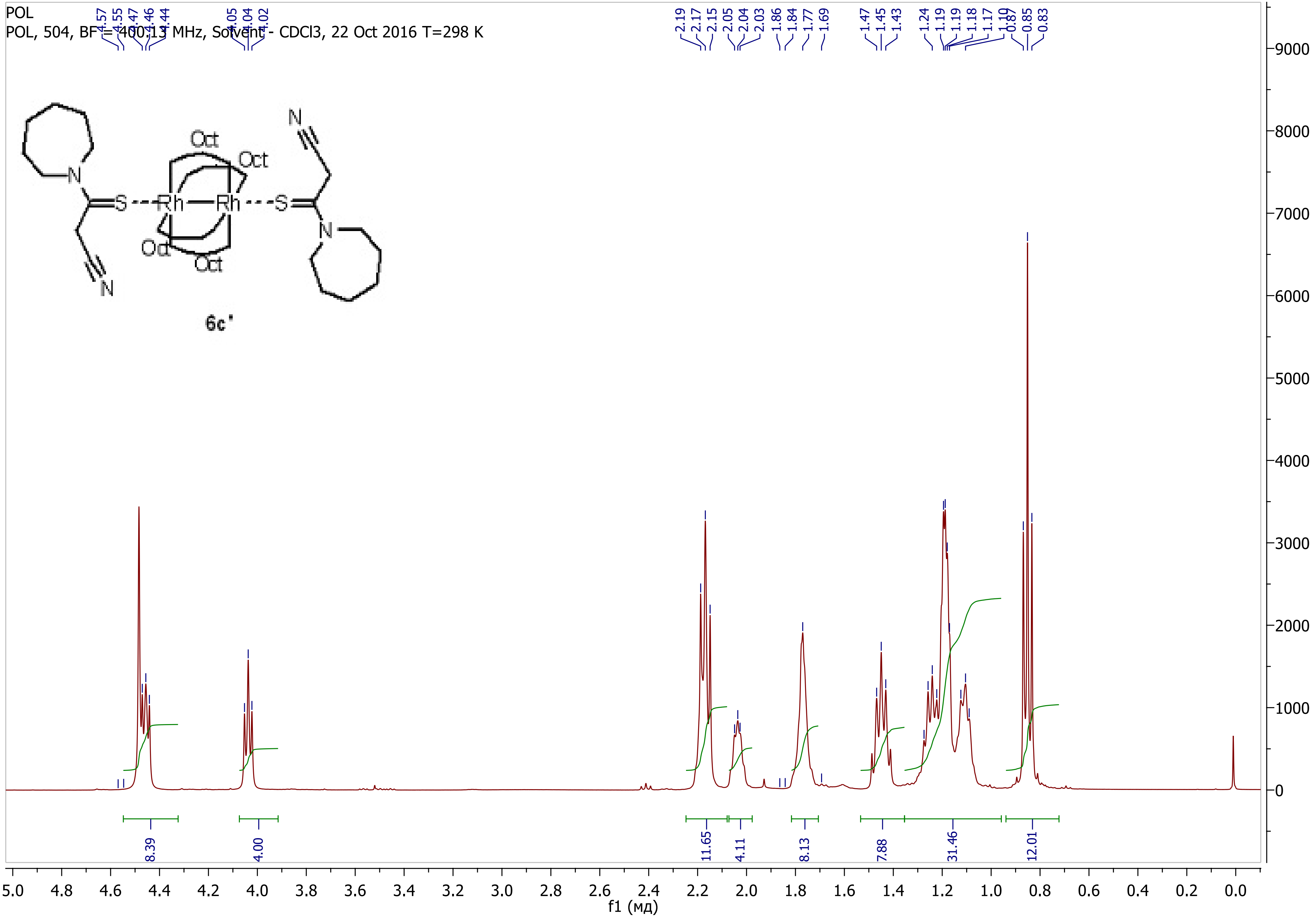
POL
POL, 504, BF = 400.13 MHz, Solvent - CDCl3, 22 Oct 2016 T=298 K
4.57 4.55 4.47 4.46 4.44 4.05 4.04 4.02
2.19 2.17 2.15 2.05 2.04 2.03 1.86 1.84 1.77 1.69
1.47 1.45 1.43 1.24 1.19 1.19 1.18 1.17 1.10 0.87 0.85 0.83
Oct
Oct
Oct
Oct
N
N
N
N
S
S
Rh
Rh
6c'
8.39
4.00
11.65
4.11
8.13
7.88
31.46
12.01
9000
8000
7000
6000
5000
4000
3000
2000
1000
0
5.0 4.8 4.6 4.4 4.2 4.0 3.8 3.6 3.4 3.2 3.0 2.8 2.6 2.4 2.2 2.0 1.8 1.6 1.4 1.2 1.0 0.8 0.6 0.4 0.2 0.0
f1 (мд)

POL
POL, 946, BF = 400.13 MHz, Solvent - CDCl3, 21 Oct 2016 T=298 K

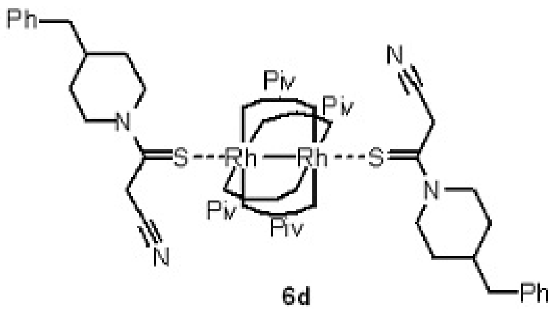

6d

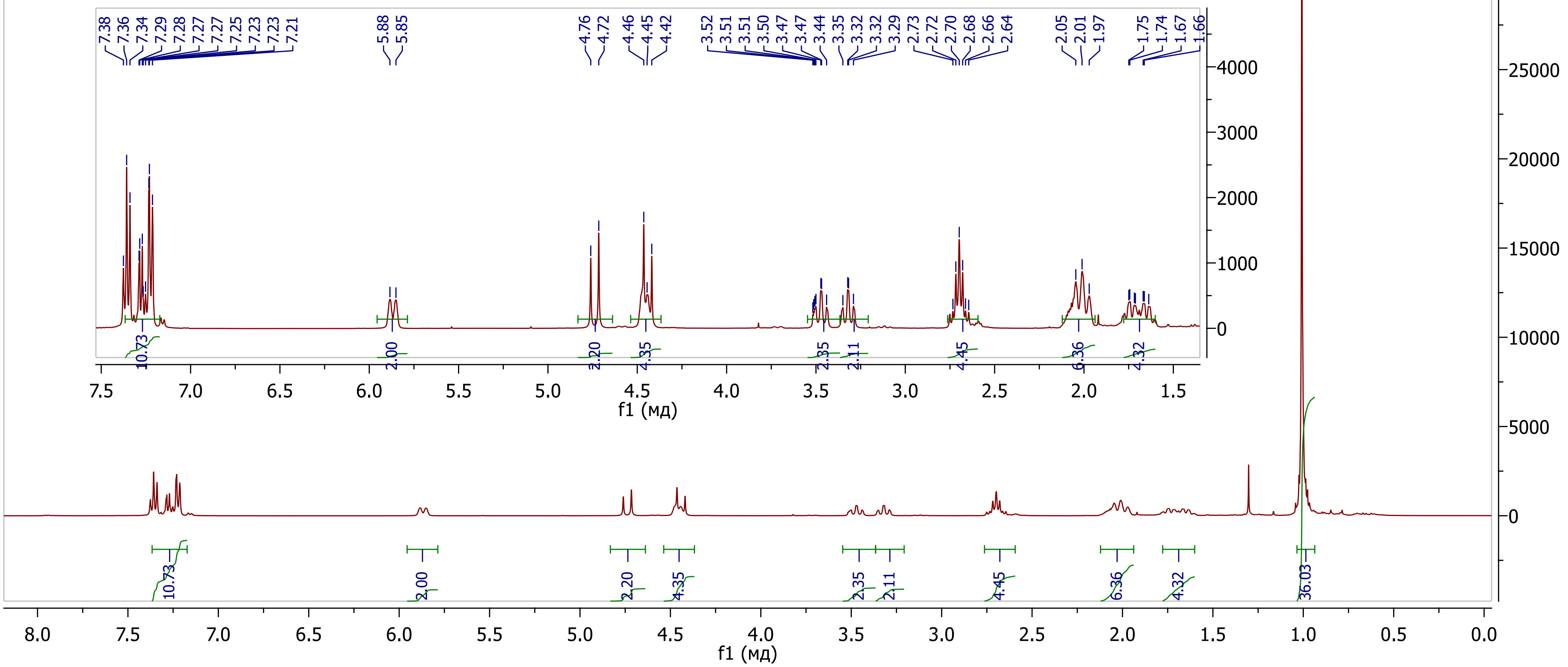

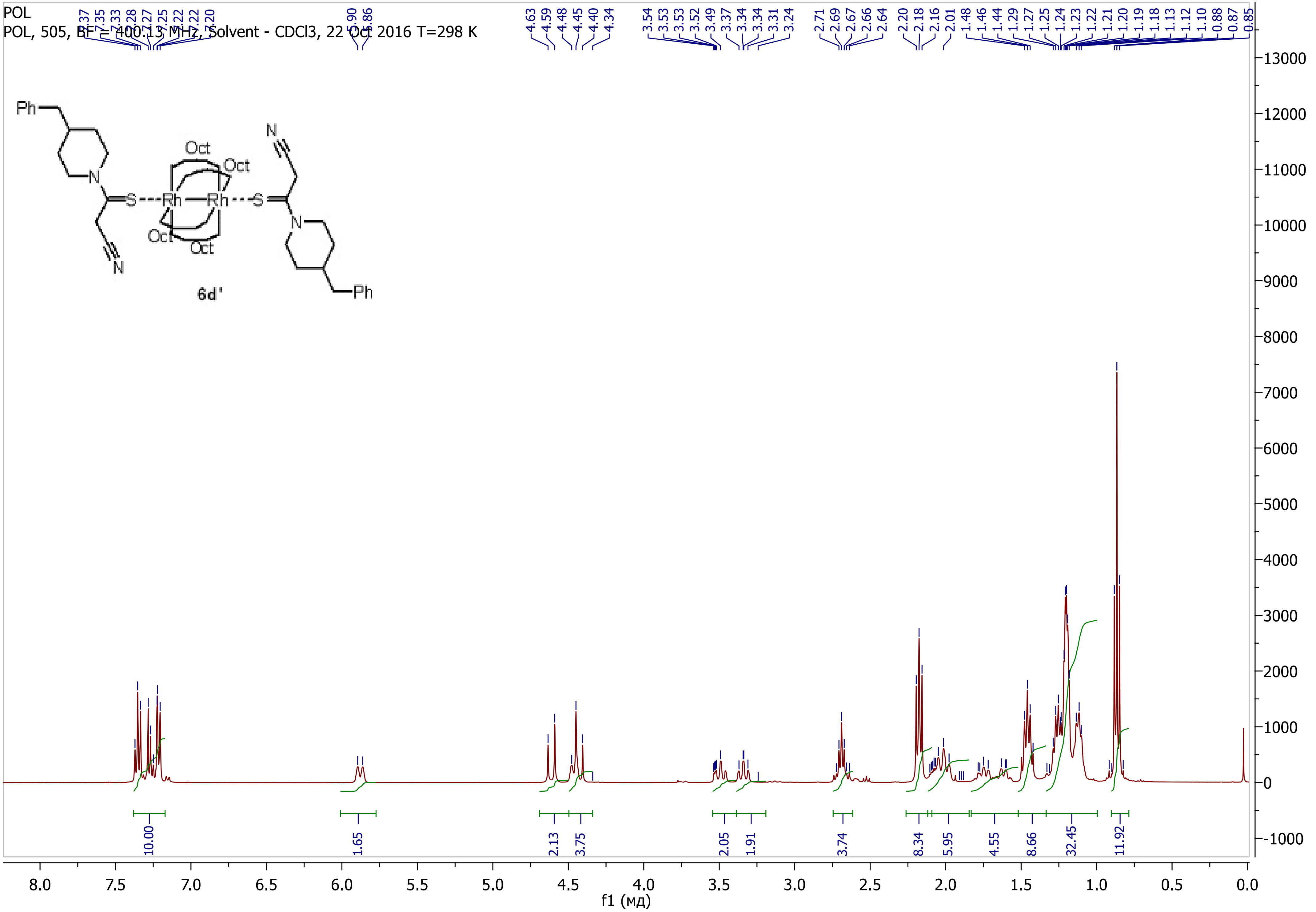
POL
POL, 505, BF = 400.13 MHz, Solvent - CDCl3, 22 Oct 2016 T=298 K
7.37 7.35 7.33 7.28 7.27 7.25 7.22 7.22 7.20
5.90 5.86
4.63 4.59 4.48 4.45 4.40 4.34
3.54 3.53 3.53 3.52 3.49 3.37 3.34 3.34 3.31 3.24
2.71 2.69 2.67 2.66 2.64 2.20 2.18 2.16 2.01
1.48 1.46 1.44 1.29 1.27 1.25 1.24 1.23 1.22 1.21 1.20 1.19 1.18 1.13 1.12 1.10 0.88 0.87 0.85
Ph
N
Oct
Rh
S
6d'
10.00
1.65
2.13
3.75
2.05
1.91
3.74
8.34
5.95
4.55
8.66
32.45
11.92
f1 (мд)

POL
POL, 938, BF = 400.13 MHz, Solvent - CDCl3, 18 Oct 2016 T=298 K

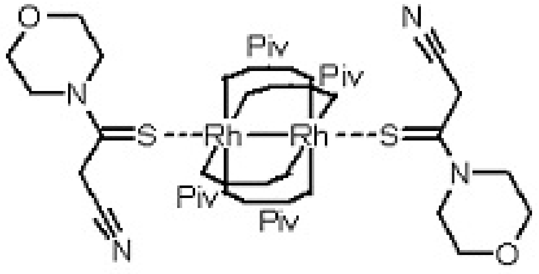

6 e

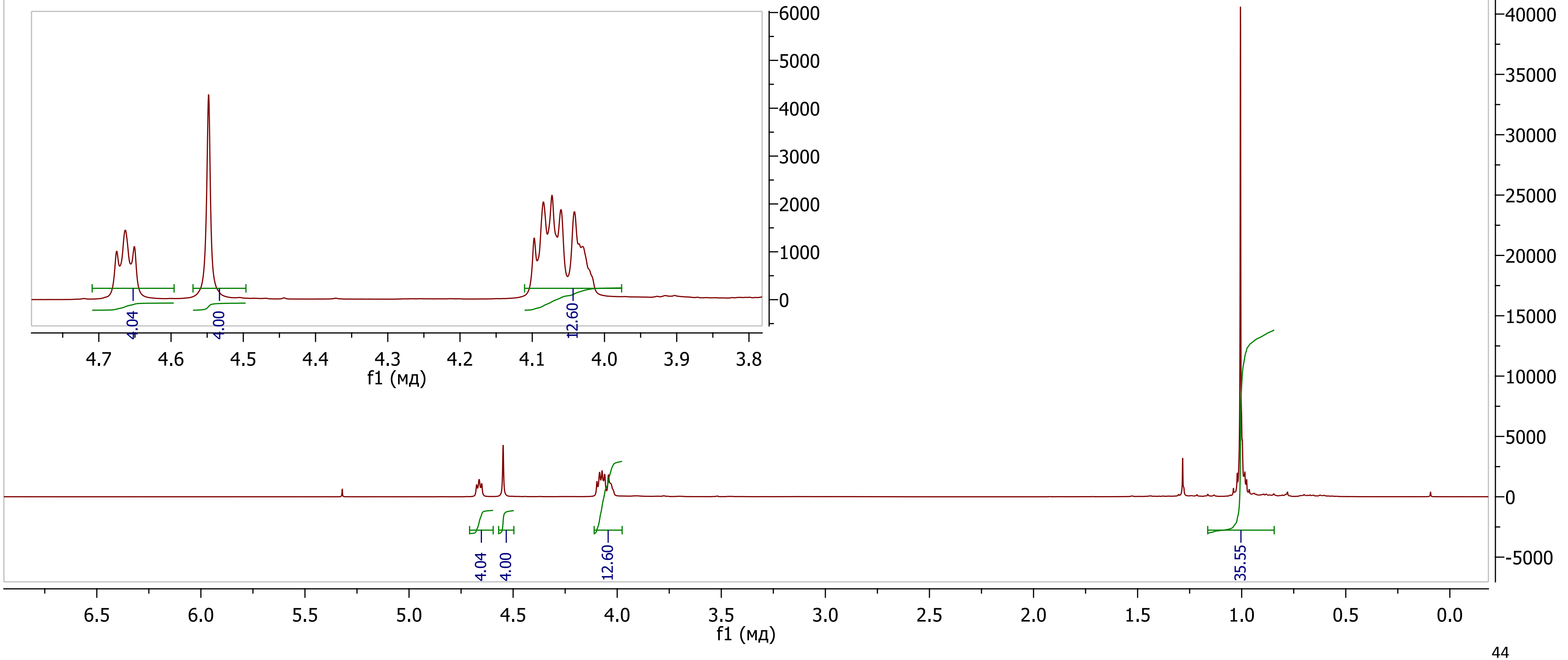

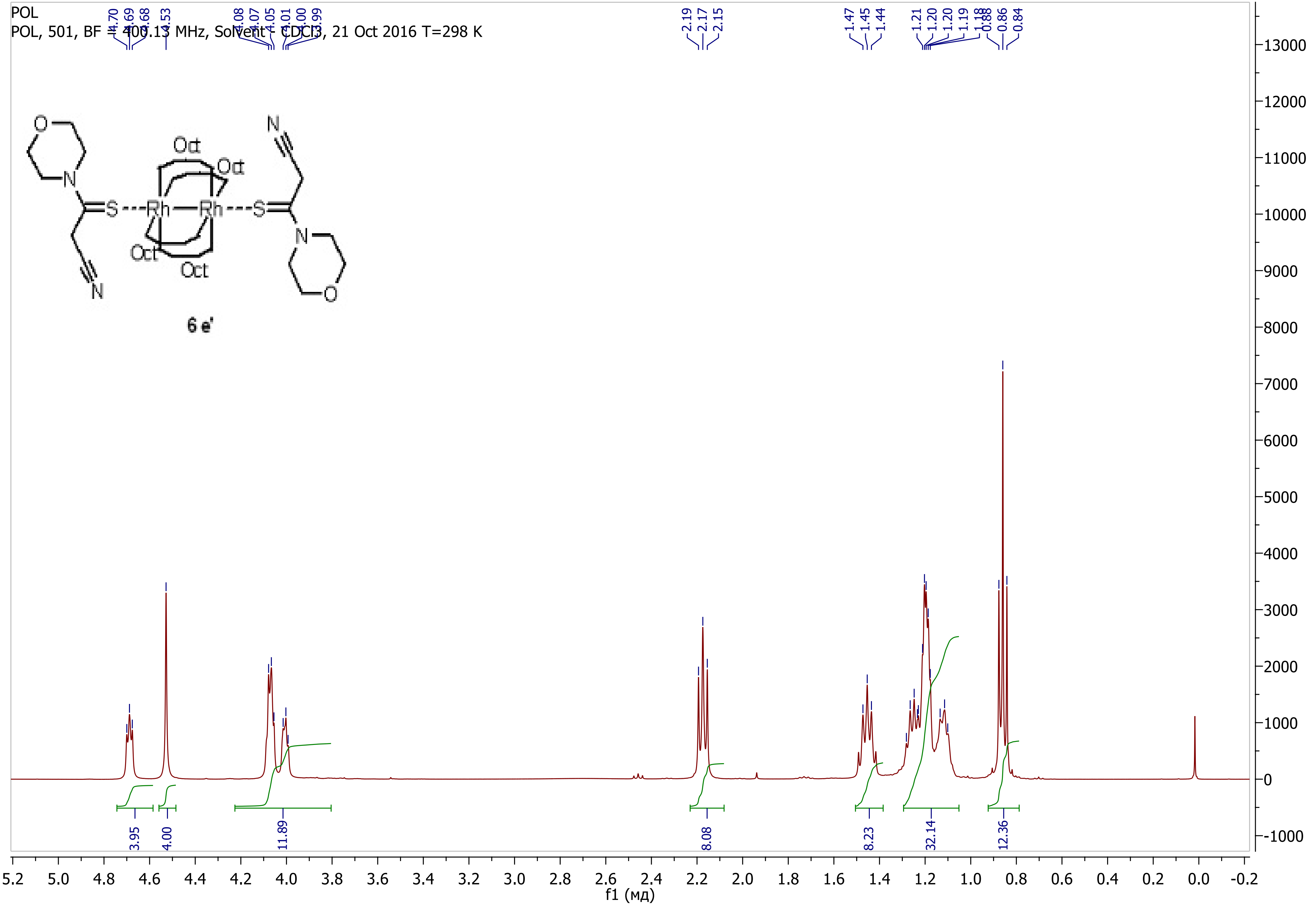
POL
POL, 501, BF = 400.13 MHz, Solvent - CDCl3, 21 Oct 2016 T=298 K
4.70
4.69
4.68
4.53
4.08
4.07
4.05
4.01
4.00
3.99
2.19
2.17
2.15
1.47
1.45
1.44
1.21
1.20
1.20
1.19
1.18
0.88
0.86
0.84
Oct
Rh
S
N
O
6 e'
3.95
4.00
11.89
8.08
8.23
32.14
12.36
f1 (мд)

Crystal structure determination of [3b]

Crystal Data for $C_{13}H_{18}N_2O_4S$ (M =298.35 g/mol): monoclinic, space group P2$_1$/n (no. 14), a = 8.8939(4) Å, b = 11.3076(4) Å, c = 14.5871(6) Å, β = 96.987(4)°, V = 1456.11(10) Å^3, Z = 4, T = 100(2) K, μ(MoKα) = 0.237 mm^{-1}, $Dcalc$ = 1.361 g/cm^3, 18831 reflections measured (5.628° ≤ 2Θ ≤ 54.992°), 3344 unique (R_{int} = 0.0238, R_{sigma} = 0.0161) which were used in all calculations. The final R_1 was 0.0441 (I > 2σ(I)) and wR_2 was 0.1196 (all data). CCDC number 1547408.

Crystal structure determination of [4a]

Crystal Data for $C_{15}H_{20}N_2O_6S$ (M =356.39 g/mol): monoclinic, space group P2$_1$/c (no. 14), a = 10.9175(2) Å, b = 21.4942(4) Å, c = 14.1917(3) Å, β = 98.287(2)°, V = 3295.49(12) Å^3, Z = 8, T = 100(2) K, μ(MoKα) = 0.231 mm^{-1}, $Dcalc$ = 1.437 g/cm^3, 15019 reflections measured (5.802° ≤ 2Θ ≤ 54.998°), 7428 unique (R_{int} = 0.0278, R_{sigma} = 0.0403) which were used in all calculations. The final R_1 was 0.0395 (I > 2σ(I)) and wR_2 was 0.1027 (all data). CCDC number 1547409.

Crystal structure determination of [5c]

Crystal Data for $C_{14}H_{19}N_3O_2S$ (M =293.38 g/mol): monoclinic, space group P2$_1$/c (no. 14), a = 8.0485(3) Å, b = 21.6755(7) Å, c = 8.4863(3) Å, β = 104.132(4)°, V = 1435.66(9) Å^3, Z = 4, T = 100(2) K, μ(MoKα) = 0.231 mm^{-1}, $Dcalc$ = 1.357 g/cm^3, 26370 reflections measured (5.294° ≤ 2Θ ≤ 54.994°), 3293 unique (R_{int} = 0.0353, R_{sigma} = 0.0181) which were used in all calculations. The final R_1 was 0.0313 (I > 2σ(I)) and wR_2 was 0.0767 (all data). CCDC number 1547410.

Crystal structure determination of [7e]

Crystal Data for $C_{34}H_{56}N_4O_{10}Rh_2S_2$ (M =950.76 g/mol): monoclinic, space group C2/c (no. 15), a = 22.6462(10) Å, b = 22.3853(10) Å, c = 9.7618(5) Å, β = 99.962(4)°, V = 4874.0(4) Å^3, Z = 4, T = 100(2) K, μ(MoKα) = 0.810 mm^{-1}, $Dcalc$ = 1.296 g/cm^3, 20526 reflections measured (5.222° ≤ 2Θ ≤ 54.994°), 5602 unique (R_{int} = 0.0286, R_{sigma} = 0.0287) which were used in all calculations. The final R_1 was 0.0270 (I > 2σ(I)) and wR_2 was 0.0661 (all data). CCDC number 1547411.

Identification code	3b	4a
Empirical formula	$C_{13}H_{18}N_2O_4S$	$C_{15}H_{20}N_2O_6S$
Formula weight	298.35	356.39
Temperature/K	100(2)	100(2)
Crystal system	monoclinic	monoclinic
Space group	$P2_1/n$	$P2_1/c$
a/Å	8.8939(4)	10.9175(2)
b/Å	11.3076(4)	21.4942(4)
c/Å	14.5871(6)	14.1917(3)
α/°	90	90
β/°	96.987(4)	98.287(2)
γ/°	90	90
Volume/Å^3	1456.11(10)	3295.49(12)
Z	4	8
ρ_{calc}g/cm^3	1.361	1.437
μ/mm^{-1}	0.237	0.231
F(000)	632.0	1504.0
Crystal size/mm^3	0.2 × 0.2 × 0.2	0.25 × 0.2 × 0.2
Radiation	MoKα (λ = 0.71073)	MoKα (λ = 0.71073)
2Θ range for data collection/°	5.628 to 54.992	5.802 to 54.998
Index ranges	$-11 \leq h \leq 11$, $-14 \leq k \leq 14$, $-18 \leq l \leq 18$	$-14 \leq h \leq 5$, $-27 \leq k \leq 16$, $-18 \leq l \leq 18$
Reflections collected	18831	15019
Independent reflections	3344 [R_{int} = 0.0238, R_{sigma} = 0.0161]	7428 [R_{int} = 0.0278, R_{sigma} = 0.0403]
Data/restraints/parameters	3344/0/183	7428/0/439
Goodness-of-fit on F^2	1.049	1.027
Final R indexes [I>=2σ (I)]	R_1 = 0.0441, wR_2 = 0.1143	R_1 = 0.0395, wR_2 = 0.0939
Final R indexes [all data]	R_1 = 0.0504, wR_2 = 0.1196	R_1 = 0.0526, wR_2 = 0.1027
Largest diff. peak/hole / e Å^{-3}	1.29/-0.24	0.56/-0.35
CCDC number	1547408	1547409

Identification code	5c	7e
Empirical formula	$C_{14}H_{19}N_3O_2S$	$C_{34}H_{56}N_4O_{10}Rh_2S_2$
Formula weight	293.38	950.76
Temperature/K	100(2)	100(2)
Crystal system	monoclinic	monoclinic
Space group	$P2_1/c$	C2/c
a/Å	8.0485(3)	22.6462(10)
b/Å	21.6755(7)	22.3853(10)
c/Å	8.4863(3)	9.7618(5)
α/°	90	90
β/°	104.132(4)	99.962(4)
γ/°	90	90
Volume/Å^3	1435.66(9)	4874.0(4)
Z	4	4
ρ_{calc}g/cm^3	1.357	1.296
μ/mm^{-1}	0.231	0.810
F(000)	624.0	1960.0
Crystal size/mm^3	0.25 × 0.25 × 0.2	0.15 × 0.1 × 0.1
Radiation	MoKα (λ = 0.71073)	MoKα (λ = 0.71073)
2Θ range for data collection/°	5.294 to 54.994	5.222 to 54.994
Index ranges	-10 ≤ h ≤ 10, -28 ≤ k ≤ 28, -11 ≤ l ≤ 11	-29 ≤ h ≤ 29, -28 ≤ k ≤ 29, -12 ≤ l ≤ 12
Reflections collected	26370	20526
Independent reflections	3293 [R_{int} = 0.0353, R_{sigma} = 0.0181]	5602 [R_{int} = 0.0286, R_{sigma} = 0.0287]
Data/restraints/parameters	3293/0/182	5602/0/241
Goodness-of-fit on F^2	1.058	1.034
Final R indexes [I>=2σ (I)]	R_1 = 0.0313, wR_2 = 0.0746	R_1 = 0.0270, wR_2 = 0.0631
Final R indexes [all data]	R_1 = 0.0348, wR_2 = 0.0767	R_1 = 0.0330, wR_2 = 0.0661
Largest diff. peak/hole / e Å^{-3}	0.36/-0.23	0.54/-0.47
CCDC number	1547410	1547411

checkCIF/PLATON report

Structure factors have been supplied for datablock(s) 3b

THIS REPORT IS FOR GUIDANCE ONLY. IF USED AS PART OF A REVIEW PROCEDURE FOR PUBLICATION, IT SHOULD NOT REPLACE THE EXPERTISE OF AN EXPERIENCED CRYSTALLOGRAPHIC REFEREE.

No syntax errors found. CIF dictionary Interpreting this report

Datablock: 3b

```
Bond precision:  C-C = 0.0026 A          Wavelength=0.71073

Cell:            a=8.8939(4)     b=11.3076(4)       c=14.5871(6)
                 alpha=90        beta=96.987(4)     gamma=90
Temperature:     100 K

                 Calculated                  Reported
Volume           1456.11(10)                 1456.11(10)
Space group      P 21/n                      P 1 21/n 1
Hall group       -P 2yn                      -P 2yn
Moiety formula   C13 H18 N2 O4 S             C13 H18 N2 O4 S
Sum formula      C13 H18 N2 O4 S             C13 H18 N2 O4 S
Mr               298.35                      298.35
Dx,g cm-3        1.361                       1.361
Z                4                           4
Mu (mm-1)        0.237                       0.237
F000             632.0                       632.0
F000'            632.79
h,k,lmax         11,14,18                    11,14,18
Nref             3346                        3344
Tmin,Tmax        0.954,0.954                 0.927,1.000
Tmin'            0.954

Correction method= # Reported T Limits: Tmin=0.927 Tmax=1.000
AbsCorr = MULTI-SCAN

Data completeness= 0.999          Theta(max)= 27.496

R(reflections)= 0.0441( 2962)     wR2(reflections)= 0.1196( 3344)

S = 1.049                   Npar= 183
```

The following ALERTS were generated. Each ALERT has the format
test-name_ALERT_alert-type_alert-level.
Click on the hyperlinks for more details of the test.

Alert level B

```
PLAT094_ALERT_2_B Ratio of Maximum / Minimum Residual Density ....       5.33 Report
```

Alert level C

```
DIFMX02_ALERT_1_C  The maximum difference density is > 0.1*ZMAX*0.75
            The relevant atom site should be identified.
PLAT097_ALERT_2_C Large Reported Max.  (Positive) Residual Density       1.29 eA-3
PLAT906_ALERT_3_C Large K value in the Analysis of Variance ......      2.136 Check
PLAT975_ALERT_2_C Check Calcd Residual Density  0.87A From     O17       0.60 eA-3
```

Alert level G

```
PLAT007_ALERT_5_G Number of Unrefined Donor-H Atoms .............          1 Report
PLAT910_ALERT_3_G Missing # of FCF Reflection(s) Below Theta(Min)          2 Note
PLAT978_ALERT_2_G Number C-C Bonds with Positive Residual Density.         7 Note
```

```
   0 ALERT level A = Most likely a serious problem - resolve or explain
   1 ALERT level B = A potentially serious problem, consider carefully
   4 ALERT level C = Check. Ensure it is not caused by an omission or oversight
   3 ALERT level G = General information/check it is not something unexpected

   1 ALERT type 1 CIF construction/syntax error, inconsistent or missing data
   4 ALERT type 2 Indicator that the structure model may be wrong or deficient
   2 ALERT type 3 Indicator that the structure quality may be low
   0 ALERT type 4 Improvement, methodology, query or suggestion
   1 ALERT type 5 Informative message, check
```

It is advisable to attempt to resolve as many as possible of the alerts in all categories. Often the minor alerts point to easily fixed oversights, errors and omissions in your CIF or refinement strategy, so attention to these fine details can be worthwhile. In order to resolve some of the more serious problems it may be necessary to carry out additional measurements or structure refinements. However, the purpose of your study may justify the reported deviations and the more serious of these should normally be commented upon in the discussion or experimental section of a paper or in the "special_details" fields of the CIF. checkCIF was carefully designed to identify outliers and unusual parameters, but every test has its limitations and alerts that are not important in a particular case may appear. Conversely, the absence of alerts does not guarantee there are no aspects of the results needing attention. It is up to the individual to critically assess their own results and, if necessary, seek expert advice.

Publication of your CIF in IUCr journals

A basic structural check has been run on your CIF. These basic checks will be run on all CIFs submitted for publication in IUCr journals (*Acta Crystallographica*, *Journal of Applied Crystallography*, *Journal of Synchrotron Radiation*); however, if you intend to submit to *Acta Crystallographica Section C* or *E* or *IUCrData*, you should make sure that full publication checks are run on the final version of your CIF prior to submission.

Publication of your CIF in other journals

Please refer to the *Notes for Authors* of the relevant journal for any special instructions relating to CIF submission.

PLATON version of 27/03/2017; check.def file version of 24/03/2017

Datablock 3b - ellipsoid plot

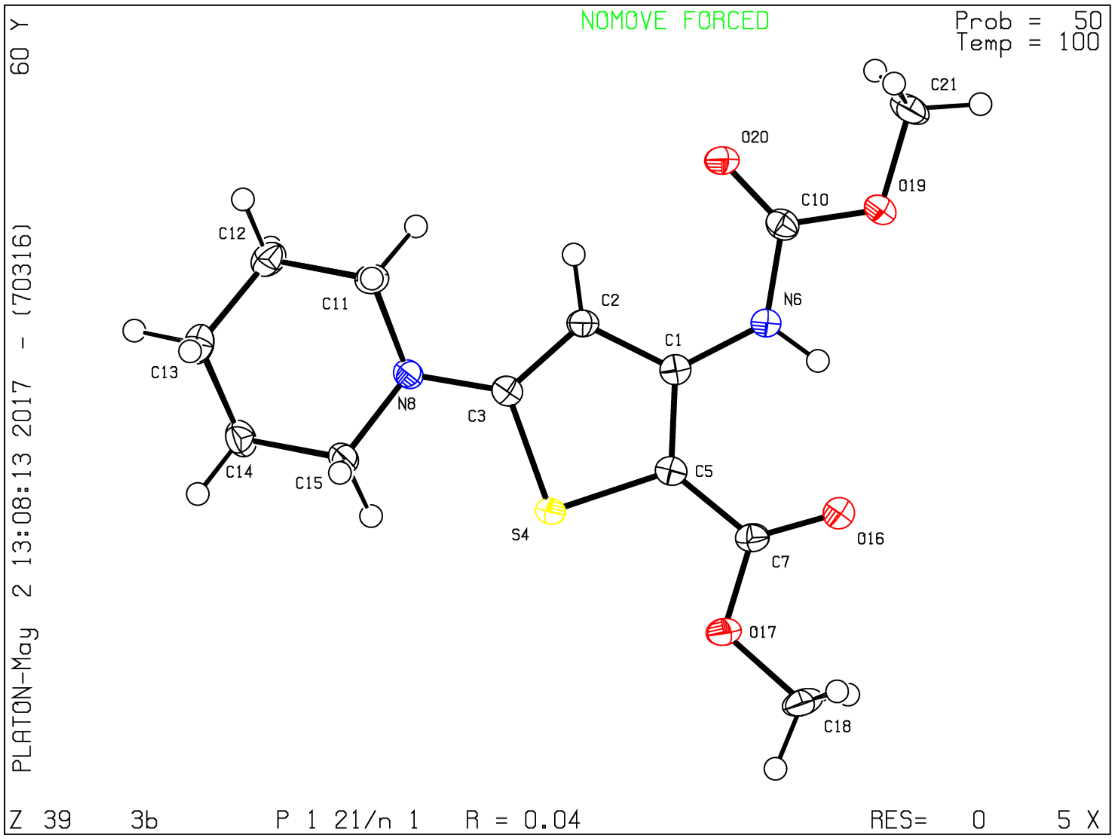

checkCIF/PLATON report

Structure factors have been supplied for datablock(s) 4a

THIS REPORT IS FOR GUIDANCE ONLY. IF USED AS PART OF A REVIEW PROCEDURE FOR PUBLICATION, IT SHOULD NOT REPLACE THE EXPERTISE OF AN EXPERIENCED CRYSTALLOGRAPHIC REFEREE.

No syntax errors found. CIF dictionary Interpreting this report

Datablock: 4a

```
Bond precision:  C-C = 0.0022 A          Wavelength=0.71073

Cell:            a=10.9175(2)     b=21.4942(4)      c=14.1917(3)
                 alpha=90         beta=98.287(2)    gamma=90
Temperature:     100 K

                Calculated                Reported
Volume          3295.49(11)               3295.49(12)
Space group     P 21/c                    P 1 21/c 1
Hall group      -P 2ybc                   -P 2ybc
Moiety formula  C15 H20 N2 O6 S           C15 H20 N2 O6 S
Sum formula     C15 H20 N2 O6 S           C15 H20 N2 O6 S
Mr              356.39                    356.39
Dx,g cm-3       1.437                     1.437
Z               8                         8
Mu (mm-1)       0.231                     0.231
F000            1504.0                    1504.0
F000'           1505.78
h,k,lmax        14,27,18                  14,27,18
Nref            7578                      7428
Tmin,Tmax       0.946,0.955               0.951,1.000
Tmin'           0.944

Correction method= # Reported T Limits: Tmin=0.951 Tmax=1.000
AbsCorr = MULTI-SCAN

Data completeness= 0.980          Theta(max)= 27.499

R(reflections)= 0.0395( 6059)     wR2(reflections)= 0.1027( 7428)

S = 1.027                   Npar= 439
```

The following ALERTS were generated. Each ALERT has the format
test-name_ALERT_alert-type_alert-level.
Click on the hyperlinks for more details of the test.

Alert level C

```
PLAT910_ALERT_3_C Missing # of FCF Reflection(s) Below Theta(Min)          8 Note
PLAT911_ALERT_3_C Missing # FCF Refl Between THmin & STh/L=  0.600        95 Report
```

Alert level G

```
PLAT007_ALERT_5_G Number of Unrefined Donor-H Atoms .............          2 Report
PLAT912_ALERT_4_G Missing # of FCF Reflections Above STh/L=  0.600        47 Note
PLAT960_ALERT_3_G Number of Intensities with I  <   - 2*sig(I) ...         3 Check
PLAT978_ALERT_2_G Number C-C Bonds with Positive Residual Density.        12 Note
```

```
  0 ALERT level A = Most likely a serious problem - resolve or explain
  0 ALERT level B = A potentially serious problem, consider carefully
  2 ALERT level C = Check. Ensure it is not caused by an omission or oversight
  4 ALERT level G = General information/check it is not something unexpected

  0 ALERT type 1 CIF construction/syntax error, inconsistent or missing data
  1 ALERT type 2 Indicator that the structure model may be wrong or deficient
  3 ALERT type 3 Indicator that the structure quality may be low
  1 ALERT type 4 Improvement, methodology, query or suggestion
  1 ALERT type 5 Informative message, check
```

It is advisable to attempt to resolve as many as possible of the alerts in all categories. Often the minor alerts point to easily fixed oversights, errors and omissions in your CIF or refinement strategy, so attention to these fine details can be worthwhile. In order to resolve some of the more serious problems it may be necessary to carry out additional measurements or structure refinements. However, the purpose of your study may justify the reported deviations and the more serious of these should normally be commented upon in the discussion or experimental section of a paper or in the "special_details" fields of the CIF. checkCIF was carefully designed to identify outliers and unusual parameters, but every test has its limitations and alerts that are not important in a particular case may appear. Conversely, the absence of alerts does not guarantee there are no aspects of the results needing attention. It is up to the individual to critically assess their own results and, if necessary, seek expert advice.

Publication of your CIF in IUCr journals

A basic structural check has been run on your CIF. These basic checks will be run on all CIFs submitted for publication in IUCr journals (*Acta Crystallographica*, *Journal of Applied Crystallography*, *Journal of Synchrotron Radiation*); however, if you intend to submit to *Acta Crystallographica Section C* or *E* or *IUCrData*, you should make sure that full publication checks are run on the final version of your CIF prior to submission.

Publication of your CIF in other journals

Please refer to the *Notes for Authors* of the relevant journal for any special instructions relating to CIF submission.

PLATON version of 27/03/2017; check.def file version of 24/03/2017

Datablock 4a - ellipsoid plot

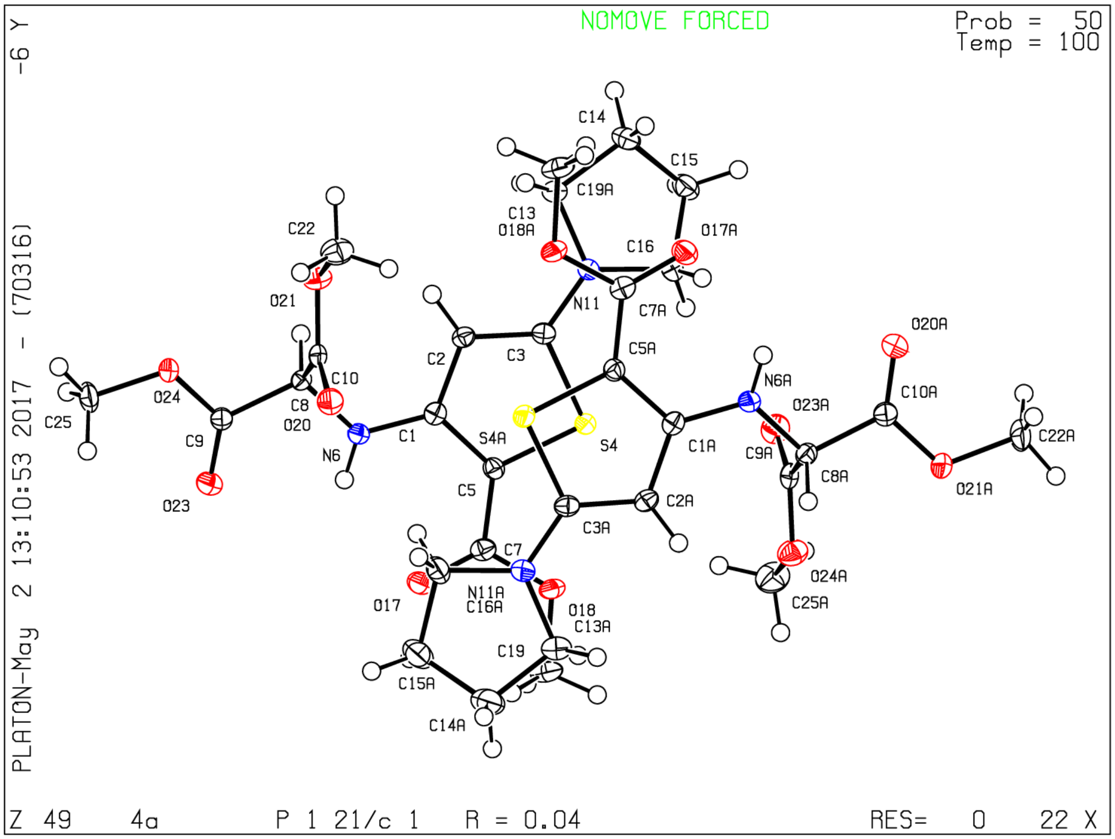

checkCIF/PLATON report

Structure factors have been supplied for datablock(s) 5c

THIS REPORT IS FOR GUIDANCE ONLY. IF USED AS PART OF A REVIEW PROCEDURE FOR PUBLICATION, IT SHOULD NOT REPLACE THE EXPERTISE OF AN EXPERIENCED CRYSTALLOGRAPHIC REFEREE.

No syntax errors found. CIF dictionary Interpreting this report

Datablock: 5c

```
Bond precision:  C-C = 0.0018 A         Wavelength=0.71073

Cell:            a=8.0485(3)     b=21.6755(7)        c=8.4863(3)
                 alpha=90        beta=104.132(4)     gamma=90
Temperature:     100 K

                Calculated                 Reported
Volume          1435.67(9)                 1435.66(9)
Space group     P 21/c                     P 1 21/c 1
Hall group      -P 2ybc                    -P 2ybc
Moiety formula  C14 H19 N3 O2 S            C14 H19 N3 O2 S
Sum formula     C14 H19 N3 O2 S            C14 H19 N3 O2 S
Mr              293.38                     293.38
Dx,g cm-3       1.357                      1.357
Z               4                          4
Mu (mm-1)       0.231                      0.231
F000            624.0                      624.0
F000'           624.72
h,k,lmax        10,28,11                   10,28,11
Nref            3294                       3293
Tmin,Tmax       0.944,0.955                0.821,1.000
Tmin'           0.944

Correction method= # Reported T Limits: Tmin=0.821 Tmax=1.000
AbsCorr = MULTI-SCAN

Data completeness= 1.000          Theta(max)= 27.497

R(reflections)= 0.0313( 3010)     wR2(reflections)= 0.0767( 3293)

S = 1.058                  Npar= 182
```

The following ALERTS were generated. Each ALERT has the format
test-name_ALERT_alert-type_alert-level.
Click on the hyperlinks for more details of the test.

checkCIF/PLATON report

Structure factors have been supplied for datablock(s) 7e

THIS REPORT IS FOR GUIDANCE ONLY. IF USED AS PART OF A REVIEW PROCEDURE FOR PUBLICATION, IT SHOULD NOT REPLACE THE EXPERTISE OF AN EXPERIENCED CRYSTALLOGRAPHIC REFEREE.

No syntax errors found. CIF dictionary Interpreting this report

Datablock: 7e

Bond precision:	C-C = 0.0034 A		Wavelength=0.71073
Cell:	a=22.6462(10)	b=22.3853(10)	c=9.7618(5)
	alpha=90	beta=99.962(4)	gamma=90
Temperature:	100 K		

	Calculated	Reported
Volume	4874.1(4)	4874.0(4)
Space group	C 2/c	C 1 2/c 1
Hall group	-C 2yc	-C 2yc
Moiety formula	C34 H56 N4 O10 Rh2 S2 [+ solvent]	C34 H56 N4 O10 Rh2 S2
Sum formula	C34 H56 N4 O10 Rh2 S2 [+ solvent]	C34 H56 N4 O10 Rh2 S2
Mr	950.77	950.76
Dx,g cm-3	1.296	1.296
Z	4	4
Mu (mm-1)	0.810	0.810
F000	1960.0	1960.0
F000’	1952.79	
h,k,lmax	29,29,12	29,29,12
Nref	5607	5602
Tmin,Tmax	0.907,0.922	0.953,1.000
Tmin’	0.886	

Correction method= # Reported T Limits: Tmin=0.953 Tmax=1.000
AbsCorr = MULTI-SCAN

Data completeness= 0.999 Theta(max)= 27.497

R(reflections)= 0.0270(4927) wR2(reflections)= 0.0661(5602)

S = 1.034 Npar= 241

The following ALERTS were generated. Each ALERT has the format
test-name_ALERT_alert-type_alert-level.
Click on the hyperlinks for more details of the test.

Alert level C

PLAT220_ALERT_2_C	Non-Solvent Resd 1 C Ueq(max)/Ueq(min) Range	4.8 Ratio
PLAT222_ALERT_3_C	Non-Solvent Resd 1 H Uiso(max)/Uiso(min) Range	5.0 Ratio
PLAT242_ALERT_2_C	Low 'MainMol' Ueq as Compared to Neighbors of	C22 Check
PLAT910_ALERT_3_C	Missing # of FCF Reflection(s) Below Theta(Min)	5 Note

Alert level G

PLAT083_ALERT_2_G	SHELXL Second Parameter in WGHT Unusually Large	7.84 Why ?
PLAT232_ALERT_2_G	Hirshfeld Test Diff (M-X) Rh1 -- S2 ..	10.0 s.u.
PLAT398_ALERT_2_G	Deviating C-O-C Angle from 120 Deg for O6	109.3 Degree
PLAT606_ALERT_4_G	VERY LARGE Solvent Accessible VOID(S) in Structure	! Info
PLAT869_ALERT_4_G	ALERTS Related to the use of SQUEEZE Suppressed	! Info
PLAT913_ALERT_3_G	Missing # of Very Strong Reflections in FCF	1 Note
PLAT978_ALERT_2_G	Number C-C Bonds with Positive Residual Density.	4 Note

0 **ALERT level A** = Most likely a serious problem - resolve or explain
0 **ALERT level B** = A potentially serious problem, consider carefully
4 **ALERT level C** = Check. Ensure it is not caused by an omission or oversight
7 **ALERT level G** = General information/check it is not something unexpected

0 ALERT type 1 CIF construction/syntax error, inconsistent or missing data
6 ALERT type 2 Indicator that the structure model may be wrong or deficient
3 ALERT type 3 Indicator that the structure quality may be low
2 ALERT type 4 Improvement, methodology, query or suggestion
0 ALERT type 5 Informative message, check

It is advisable to attempt to resolve as many as possible of the alerts in all categories. Often the minor alerts point to easily fixed oversights, errors and omissions in your CIF or refinement strategy, so attention to these fine details can be worthwhile. In order to resolve some of the more serious problems it may be necessary to carry out additional measurements or structure refinements. However, the purpose of your study may justify the reported deviations and the more serious of these should normally be commented upon in the discussion or experimental section of a paper or in the "special_details" fields of the CIF. checkCIF was carefully designed to identify outliers and unusual parameters, but every test has its limitations and alerts that are not important in a particular case may appear. Conversely, the absence of alerts does not guarantee there are no aspects of the results needing attention. It is up to the individual to critically assess their own results and, if necessary, seek expert advice.

Publication of your CIF in IUCr journals

A basic structural check has been run on your CIF. These basic checks will be run on all CIFs submitted for publication in IUCr journals (*Acta Crystallographica*, *Journal of Applied Crystallography*, *Journal of Synchrotron Radiation*); however, if you intend to submit to *Acta Crystallographica Section C* or *E* or *IUCrData*, you should make sure that full publication checks are run on the final version of your CIF prior to submission.

Publication of your CIF in other journals

Please refer to the *Notes for Authors* of the relevant journal for any special instructions relating to CIF submission.

PLATON version of 27/03/2017; check.def file version of 24/03/2017

Datablock 7e - ellipsoid plot

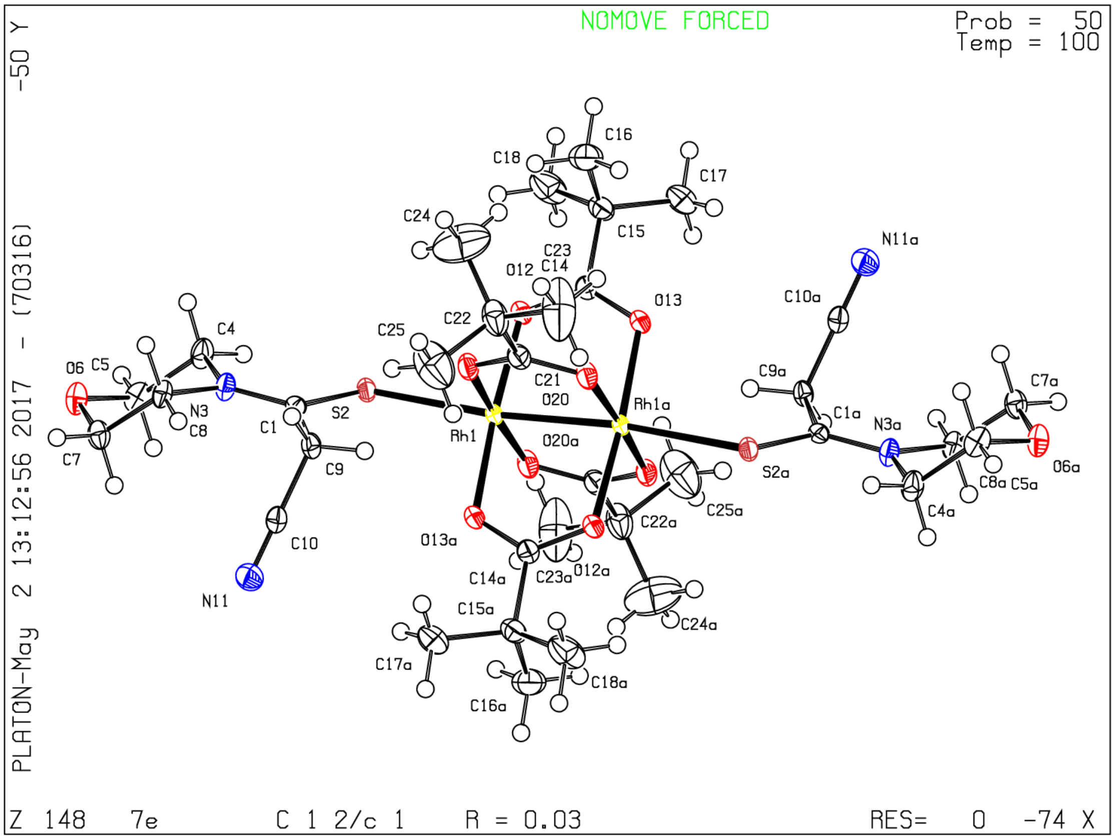

Alert level G

```
PLAT007_ALERT_5_G Number of Unrefined Donor-H Atoms .............          1 Report
PLAT230_ALERT_2_G Hirshfeld Test Diff for    C5     --  C7      ..        5.4 s.u.
PLAT910_ALERT_3_G Missing # of FCF Reflection(s) Below Theta(Min)          2 Note
PLAT978_ALERT_2_G Number C-C Bonds with Positive Residual Density.         8 Note
```

```
  0 ALERT level A = Most likely a serious problem - resolve or explain
  0 ALERT level B = A potentially serious problem, consider carefully
  0 ALERT level C = Check. Ensure it is not caused by an omission or oversight
  4 ALERT level G = General information/check it is not something unexpected

  0 ALERT type 1 CIF construction/syntax error, inconsistent or missing data
  2 ALERT type 2 Indicator that the structure model may be wrong or deficient
  1 ALERT type 3 Indicator that the structure quality may be low
  0 ALERT type 4 Improvement, methodology, query or suggestion
  1 ALERT type 5 Informative message, check
```

It is advisable to attempt to resolve as many as possible of the alerts in all categories. Often the minor alerts point to easily fixed oversights, errors and omissions in your CIF or refinement strategy, so attention to these fine details can be worthwhile. In order to resolve some of the more serious problems it may be necessary to carry out additional measurements or structure refinements. However, the purpose of your study may justify the reported deviations and the more serious of these should normally be commented upon in the discussion or experimental section of a paper or in the "special_details" fields of the CIF. checkCIF was carefully designed to identify outliers and unusual parameters, but every test has its limitations and alerts that are not important in a particular case may appear. Conversely, the absence of alerts does not guarantee there are no aspects of the results needing attention. It is up to the individual to critically assess their own results and, if necessary, seek expert advice.

Publication of your CIF in IUCr journals

A basic structural check has been run on your CIF. These basic checks will be run on all CIFs submitted for publication in IUCr journals (*Acta Crystallographica*, *Journal of Applied Crystallography*, *Journal of Synchrotron Radiation*); however, if you intend to submit to *Acta Crystallographica Section C* or *E* or *IUCrData*, you should make sure that full publication checks are run on the final version of your CIF prior to submission.

Publication of your CIF in other journals

Please refer to the *Notes for Authors* of the relevant journal for any special instructions relating to CIF submission.

PLATON version of 27/03/2017; check.def file version of 24/03/2017

Datablock 5c - ellipsoid plot

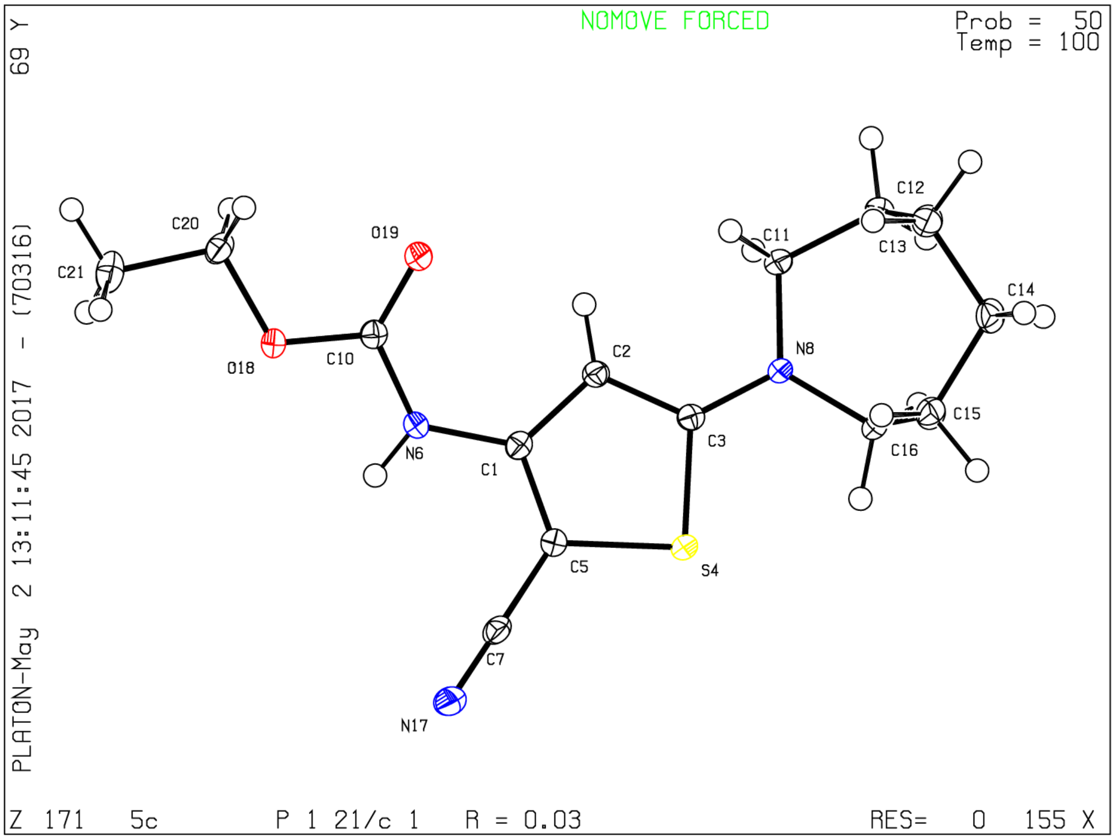

Supplement: File 1 — NMR spectra of all new compounds and data of X-ray analysis. [file Beilstein_J_Org_Chem-13-2569-s001.pdf]
