# Supplementary material for: Rh(II)-mediated domino [4 + 1]-annulation of α-cyanothioacetamides using diazoesters: A new entry for the synthesis of multisubstituted thiophenes
Source: Beilstein J Org Chem. 2017 Nov 30;13:2569–76. doi: 10.3762/bjoc.13.253 (PMC5727864; doi:10.3762/bjoc.13.253)
Supplement: File 2 — Experimental procedures and characterization data of all new compounds. [file Beilstein_J_Org_Chem-13-2569-s002.pdf]

Supporting Information

for

Rh(II)-mediated domino [4 + 1]-annulation of α-cyanothioacetamides using diazoesters: A new entry for the synthesis of multisubstituted thiophenes

Jury J. Medvedev,[1] Ilya V. Efimov,[2] Yuri M. Shafran,[2] Vitaliy V. Suslonov,[3] Vasiliy A. Bakulev*[2] and Valerij A. Nikolaev *[1]

Address: [1]Department of Organic Chemistry, St-Petersburg State University, 26 University pr., 198504, Saint-Petersburg, Russia, [2]Institute of Chemistry and Technology, Ural Federal University, 19 Mira Str., 620002, Ekaterinburg, Russia and [3]Center for X-ray Diffraction Studies, St-Petersburg State University, 26 University pr., 198504, Saint-Petersburg, Russia.

Email: V. A. Nikolaev* - valerij.nikolaev@gmail.com; V. A. Bakulev* - v.a.bakulev@urfu.ru

*Corresponding author

Experimental procedures and characterization data of all new compounds

All reactions were carried out under an argon atmosphere in solvents dried and purified before use by common methods. Monitoring of the reaction course was accomplished by thin layer chromatography (TLC) on silica gel SIL G/UV254 plates (Marchery, Nagel & Co.). Flash chromatography was performed using Merck silica gel 60 or 230–400 mesh (eluent: hexane/DCM). IR spectra of compounds were measured in tetrachloromethane solution by

means of compact size FTIR spectrometer TENSOR 37 (Bruker). ^{1}H and ^{13}C NMR spectra were recorded by using of Bruker-400 Avance NMR spectrometer. Chemical shifts are reported in ppm, and coupling constants are given in Hz. All signals in the NMR spectra were normalized relative to signals of $CHCl_3$ (δ = 7.26 ppm in ^{1}H NMR) and $CDCl_3$ (δ = 77.0 in ^{13}C NMR spectra). HRMS spectra were recorded on a «MaXis» (Bruker Daltonik GmbH), the HPLC system (UHPLC) with the combined high-resolution quadrupole-time-of-flight mass spectrometer with electrospray ionization (ESI-QTOF).

For single crystal X-ray diffraction experiments of **4a**, **3b**, **5c**, **6e** an Agilent Technologies «Xcalibur» diffractometer with monochromated MoKα radiation was used. All samples were measured at 100 K. The unit cell parameters were refined by least square techniques included in the CrysAlisPro (Agilent Technologies, 2012) program suite. Empirical absorption correction was applied using spherical harmonics, implemented in SCALE3 ABSPACK scaling algorithm. The structures were solved by the Superflip [1-3] and ShelXS [4] structure solution programs using Charge Flipping and Direct Methods, respectively, then refined by means of the ShelXL [5] program, incorporated in the Olex2 program package [6]. X-ray data for compounds **4a**, **3b**, **5c**, **6e** and their CCDC are given in electronic Suppurting Information File 1. Starting diazo compounds **2a–c** [7-9], **2d** [10] and thioamides **1a–e** [11] were prepared using previously described protocols.

General procedure for Rh(II)-catalyzed decomposition of diazomalonates 2a,b in the presence of thioamides 1a–e. To a solution of thioamide **1a–e** (1.0 mmol, 1 equiv) in 5 ml of benzene (or toluene) with 11 mg of dirhodium(II) tetraacetate (2 mol %) a solution of diazomalonate **2a**,**b** (1.2 mmol, 1.2 equiv) in 1 ml of benzene (or toluene) was added dropwise. Reaction mixture was refluxed until complete decomposition of the diazo compound (controlled by TLC). Then the solvent was removed in vacuo, the obtained residue was separated by flash

chromatography (25 g of SiO_2; eluent – hexane → hexane/acetone 2:1) to afford thiophenes **3** (27–51%) and **4** (24–35%).

Methyl 3-((methoxycarbonyl)amino)-5-(pyrrolidin-1-yl)thiophene-2-carboxylate (**3a**) was prepared according to the general procedure from 154 mg of thioamide **1a** and 190 mg of diazomalonate **2a**, yield of **3a** 145 mg (51%). Colorless solid, m.p. 130-132 °C. ^{1}H NMR (400 MHz, $CDCl_3$) δ 9.84 (broad, 1H), 6.60 (s, 1H), 3.78 (s, 3H), 3.76 (s, 3H), 3.41 – 3.24 (m, 4H), 2.08 – 2.01 (m, 4H). ^{13}C NMR (101 MHz, $CDCl_3$) δ 159.3, 153.6, 94.5, 52.4, 51.0, 50.1, 25.8. HRMS (ESI) calculated for $C_{12}H_{16}N_2O_4S$ $[M+Na]^+$ 307.0728, found 307.0724.

Ethyl 3-((ethoxycarbonyl)amino)-5-(pyrrolidin-1-yl)thiophene-2-carboxylate (**3a'**) was prepared following general procedure from 154 mg of thioamide **1a** and 223 mg of diazomalonate **2b**, 14 mg of $Rh_2(Piv)_4$ was used instead of $Rh_2(OAc)_4$, yield of **3a'** 137 mg (44%). Colorless solid, m.p. 153-155 °C. ^{1}H NMR (400 MHz, $CDCl_3$) δ 9.84 (broad, 1H), 6.58 (s, 1H), 4.53 – 3.89 (m, 4H), 3.75 – 2.99 (m, 4H), 2.45 – 1.73 (m, 4H), 1.59 – 0.94 (m, 6H). ^{13}C NMR (101 MHz, $CDCl_3$) δ 159.2, 153.2, 94.5, 61.3, 59.7, 50.1, 25.8, 14.6, 14.4. HRMS (ESI) calculated for $C_{14}H_{20}N_2O_4S$ $[M+Na]^+$ 335.1041, found 335.1036.

Methyl 3-((methoxycarbonyl)amino)-5-(piperidin-1-yl)thiophene-2-carboxylate (**3b**) was prepared following general procedure from 168 mg of thioamide **1b** and 190 mg of diazomalonate **2a**, yield of **3b** 80 mg (27%). Colorless solid, m.p. 79-81 °C. ^{1}H NMR (400 MHz, $CDCl_3$) δ 9.71 (broad, 1H), 6.84 (s, 1H), 3.76 (s, 3H), 3.74 (s, 3H), 3.41 – 2.93 (m, 4H), 1.87 – 1.47 (m, 6H). ^{13}C NMR (101 MHz, $CDCl_3$) δ 163.2, 153.5, 96.4, 52.3, 51.0, 50.4, 24.8, 23.5. HRMS (ESI) calculated for $C_{13}H_{18}N_2O_4S$ $[M+H]^+$ 299.1060, found 299.1061.

Methyl 5-(azepan-1-yl)-3-((methoxycarbonyl)amino)thiophene-2-carboxylate (**3c**) was prepared according to the general procedure from 182 mg of thioamide **1c** and 190 mg of diazomalonate **2a**, yield of **3c** 108 mg (35%). Colorless solid, m.p. 102-104 °C, ^{1}H NMR (400 MHz, $CDCl_3$) δ 9.78 (broad, 1H), 6.65 (s, 1H), 3.73 (s, 3H), 3.72 (s, 3H), 3.47 – 3.31 (m, 4H),

1.85 – 1.63 (m, 4H), 1.61 – 1.42 (m, 4H). ^{13}C NMR (101 MHz, $CDCl_3$) δ 162.0, 153.5, 93.7, 52.3, 51.7, 50.8, 27.2. HRMS (ESI) calculated for $C_{14}H_{20}N_2O_4S$ $[M+H]^+$ 313.1217, found 313.1209.

Methyl 5-(4-benzylpiperidin-1-yl)-3-((methoxycarbonyl)amino)thiophene-2-carboxylate (**3d**) was prepared following general procedure from 258 mg of thioamide **1d** and 190 mg of diazomalonate **2a**, yield of **3e** 116 mg (30%). Colorless solid, m.p. 139-141 °C. ^{1}H NMR (400 MHz, $CDCl_3$) δ 9.74 (broad, 1H), 7.29 (t, J = 7.4 Hz, 2H), 7.21 (t, J = 7.3 Hz, 1H), 7.14 (d, J = 7.1 Hz, 2H), 6.86 (s, 1H), 3.78 (s, 3H), 3.76 (s, 3H), 3.72 – 3.55 (m, 2H), 3.00 – 2.80 (m, 2H), 2.64 – 2.46 (m, 2H), 1.86 – 1.63 (m, 3H), 1.52 – 1.19 (m, 2H). ^{13}C NMR (101 MHz, $CDCl_3$) δ 163.0, 153.6, 139.8, 129.1, 128.3, 126.09, 96.7, 52.4, 51.1, 49.8, 42.8, 37.3, 31.0. HRMS (ESI) calculated for $C_{20}H_{24}N_2O_4S$ $[M+Na]^+$ 411.1354, found 411.1346.

Methyl 3-((methoxycarbonyl)amino)-5-morpholinothiophene-2-carboxylate (**3e**) was prepared according to the general procedure from 170 mg of thioamide **1e** and 190 mg of diazomalonate **2a**, yield of **3e** 98 mg (33%). Colorless solid, m.p. 152-154 °C. ^{1}H NMR (400 MHz, CDCl3) δ 9.68 (broad, 1H), 6.95 (s, 1H), 3.95 – 3.56 (m, 10H), 3.36 – 3.15 (m, 4H). ^{13}C NMR (101 MHz, $CDCl_3$) δ 162.7, 153.6, 97.7, 65.9, 52.5, 51.3, 49.2. HRMS (ESI) calculated for $C_{12}H_{16}N_2O_5S$ [M+Ag]+ 406.9825, found 406.9818.

Dimethyl 2-((2-(methoxycarbonyl)-5-(pyrrolidin-1-yl)thiophen-3-yl)amino)malonate (**4a**) was prepared following general procedure from 154 mg of thioamide **1a** and 190 mg of diazomalonate **2a**, yield of **4a** 125 mg (35%). Bright-yellow solid, m.p. 125-127 °C. ^{1}H NMR (400 MHz, $CDCl_3$) δ 7.94 (broad, 1H), 5.15 (s, 1H), 4.79 (d, J = 7.7 Hz, 1H), 3.79 (s, 6H), 3.75 (s, 3H), 3.39 – 3.16 (m, 4H), 2.07 – 1.92 (m, 4H). ^{13}C NMR (101 MHz, $CDCl_3$) δ 167.2, 159.3, 88.9, 61.5, 53.2, 49.9, 25.7. HRMS (ESI) calculated for $C_{15}H_{20}N_2O_6S$ $[M+Na]^+$ 379.0940, found 379.0935.

Diethyl 2-((2-(ethoxycarbonyl)-5-(pyrrolidin-1-yl)thiophen-3-yl)amino)malonate (**4a'**) was prepared according to the general procedure from 154 mg of thioamide **1a** and 223 mg of diazomalonate **2b**, 14 mg of $Rh_2(Piv)_4$ was used instead of $Rh_2(OAc)_4$, yield of **4a'** 80 mg (20%). Colorless oil. ^1H NMR (400 MHz, $CDCl_3$) δ 7.96 (broad, 1H), 5.20 (s, 1H), 4.75 (d, J = 7.5 Hz, 1H), 4.42 – 4.07 (m, 9H), 3.45 – 3.11 (m, 6H), 2.22 – 1.87 (m, 7H), 1.46 – 1.10 (m, 15H). ^{13}C NMR (101 MHz, $CDCl_3$) δ 166.9, 89.3, 62.3, 61.9, 49.9, 25.8, 14.8, 14.0. HRMS (ESI) calculated for $C_{18}H_{26}N_2O_6S$ $[M+Na]^+$ 421.1409, found 421.1407.

Dimethyl 2-((2-(methoxycarbonyl)-5-(piperidin-1-yl)thiophen-3-yl)amino)malonate (**4b**) was prepared according to the general procedure from 168 mg of thioamide **1b** and 190 mg of diazomalonate **2a**, yield of **4b** 94 mg (25%). Colorless solid, m.p. 130-132 °C. ^1H NMR (400 MHz, CDCl3) δ 7.88 (broad, 1H), 5.42 (s, 1H), 4.76 (d, J = 7.6 Hz, 1H), 3.79 (s, 6H), 3.75 (s, 3H), 3.27 – 3.06 (m, 4H), 1.77 – 1.45 (m, 6H). ^{13}C NMR (101 MHz, CDCl3) δ 167.14 (s), 163.4, 91.1, 61.4, 53.2, 50.6, 50.1, 24.8, 23.4. HRMS (ESI) calculated for $C_{16}H_{22}N_2O_6S$ $[M+Na]^+$ 393.1091, found 393.1087.

Dimethyl 2-((5-(azepan-1-yl)-2-(methoxycarbonyl)thiophen-3-yl)amino)malonate (**4c**) was prepared following general procedure from 182 mg of thioamide **1c** and 190 mg of diazomalonate **2a**, yield of **4c** 99 mg (26%), bright-yellow oil. ^1H NMR (400 MHz, CDCl3) δ 7.89 (broad, 1H), 5.21 (s, 1H), 4.77 (d, J = 7.6 Hz, 1H), 3.77 (s, 6H), 3.72 (s, 3H), 3.40 – 3.24 (m, 4H), 1.85 – 1.65 (m, 4H), 1.62 – 1.39 (m, 4H). ^{13}C NMR (101 MHz, $CDCl_3$) δ 167.1, 161.9, 88.3, 61.3, 53.1, 51.6, 50.4, 27.2, 27.2. HRMS (ESI) calculated for $C_{17}H_{24}N_2O_6S$ $[M+H]^+$ 385.1433, found 385.1424.

Dimethyl 2-((5-(4-benzylpiperidin-1-yl)-2-(methoxycarbonyl)-thiophen-3-yl)amino)malonate (**4d**), was prepared according to the general procedure from 258 mg of thioamide **1d** and 190 mg of diazomalonate **2a**, yield of **3e** 110 mg (24%). Bright yellow oil. ^1H NMR (400 MHz, $CDCl_3$) δ 7.91 (broad, 1H), 7.29 (t, J = 7.4 Hz, 2H), 7.21 (t, J = 7.3 Hz, 1H),

7.14 (d, J = 7.1 Hz, 2H), 5.44 (s, 1H), 4.78 (d, J = 7.6 Hz, 1H), 3.81 (s, 6H), 3.75 (s, 3H), 3.56 (d, J = 12.5 Hz, 2H), 2.84 (td, J = 12.6, 2.4 Hz, 2H), 2.56 (d, J = 6.6 Hz, 2H), 1.84 – 1.65 (m, 3H), 1.49 – 1.28 (m, 2H). ^{13}C NMR (101 MHz, $CDCl_3$) δ 167.2, 163.2, 139.8, 129.1, 128.3, 126.1, 91.5, 61.5, 53.3, 49.6, 42.8, 37.2, 31.0. HRMS (ESI) calculated for $C_{23}H_{28}N_2O_6S$ $[M+Na]^+$ 483.1566, found 483.1563.

Dimethyl 2-((2-(methoxycarbonyl)-5-morpholinothiophen-3-yl)amino)malonate (**4e**) was prepared following general procedure from 170 mg of thioamide **1e** and 190 mg of diazomalonate **2a**, yield of **4e** 100 mg (27%), bright-yellow oil. ^{1}H NMR (400 MHz, $CDCl_3$) δ 7.80 (broad, 1H), 5.53 (s, 1H), 4.78 (d, J = 3.6 Hz, 1H), 3.87 – 3.70 (m, 13H), 3.34 – 3.02 (m, 4H). ^{13}C NMR (101 MHz, $CDCl_3$) δ 167.1, 163.2, 92.3, 65.9, 61.4, 53.3, 50.9, 49.0. HRMS (ESI) calculated for $C_{15}H_{20}N_2O_7S$ $[M+Ag]^+$ 479.0037, found 479.0035.

General procedure for Rh(II)-catalyzed decomposition of α-cyanodiazoacetate 2d in the presence of thioamides 1a–e. To a solution of thioamide **1a-e** (0.5 mmol, 1 equiv) in 5 ml of DCM with 4 mg of dirhodium(II) pivalate (0.5 mol %) a solution of α-cyanodiazoacetate **2d** (1.25 mmol, 2.5 equiv) in 1 ml of DCM was added dropwise. The reaction mixture was refluxed until complete decomposition of the diazo compound (controlled by TLC). Then the solvent was removed in vacuo, the obtained residue was separated by flash chromatography (15 g of SiO_2; eluent – hexane → hexane/acetone 10:1) to afford thiophene **5** (28–67%).

Ethyl (2-cyano-5-(pyrrolidin-1-yl)thiophen-3-yl)carbamate (**5a**) was prepared according to the general procedure from 77 mg of thioamide **1a** and 210 mg of α-cyanodiazoacetate **2d** (3 equiv was used in this case), yield of **5a** 48 mg (36%). Colorless solid, m.p. 167-169 °C. ^{1}H NMR (400 MHz, $CDCl_3$) δ 7.11 (broad, 1H), 6.40 (s, 1H), 4.21 (q, J = 7.1 Hz, 2H), 3.41 – 3.18 (m, 4H), 2.13 – 1.97 (m, 4H), 1.30 (t, J = 7.1 Hz, 3H). ^{13}C NMR (101 MHz, $CDCl_3$) δ 158.8, 152.4, 147.4, 116.0, 93.4, 70.3, 61.9, 50.3, 25.8, 14.3. HRMS (ESI) calculated for $C_{12}H_{15}N_3O_2S$ $[M+Na]^+$ 288.0777, found 288.0771.

Ethyl (2-cyano-5-(piperidin-1-yl)thiophen-3-yl)carbamate (**5b**) was prepared following general procedure from 84 mg of thioamide **1b** and 170 mg of α-cyanodiazoacetate **2d**, yield of **5b** 62 mg (44%). Colorless solid, m.p. 137-139 °C. ^{1}H NMR (400 MHz, $CDCl_3$) δ 7.09 (broad, 1H), 6.72 (s, 1H), 4.22 (q, J = 7.1 Hz, 2H), 3.32 – 3.09 (m, 4H), 1.77 – 1.55 (m, 6H), 1.31 (t, J = 7.1 Hz, 3H). ^{13}C NMR (101 MHz, $CDCl_3$) δ 162.8, 152.4, 147.3, 115.4, 96.1, 72.2, 62.0, 50.8, 24.8, 23.4, 14.4. HRMS (ESI) calculated for $C_{13}H_{17}N_3O_2S$ $[M+Ag]^+$ 386.0087, found 386.0084.

Ethyl (5-(azepan-1-yl)-2-cyanothiophen-3-yl)carbamate (**5c**) was prepared according to the general procedure from 91 mg of thioamide **1c** and 170 mg of α-cyanodiazoacetate **2d**, yield of **5c** 68 mg (46%). Colorless solid, m.p. 137-139 °C. ^{1}H NMR (400 MHz, $CDCl_3$) δ 7.11 (broad, 1H), 6.49 (s, 1H), 4.20 (q, J = 7.1 Hz, 2H), 3.45 – 3.25 (m, 4H), 1.86 – 1.71 (m, 4H), 1.66 – 1.49 (m, 4H), 1.30 (t, J = 7.1 Hz, 3H). ^{13}C NMR (101 MHz, $CDCl_3$) δ 161.5, 152.4, 147.6, 115.9, 92.8, 69.6, 61.9, 51.9, 27.3, 27.2, 14.3. HRMS (ESI) calculated for $C_{14}H_{19}N_3O_2S$ $[M+Ag]^+$ 400.0243, found 400.0250.

Ethyl (5-(4-benzylpiperidin-1-yl)-2-cyanothiophen-3-yl)carbamate (**5d)** was prepared following general procedure from 129 mg of thioamide **1d** and 170 mg of α-cyanodiazoacetate **2d**, yield of **5c** 94 mg (51%), bright yellow oil. ^{1}H NMR (400 MHz, $CDCl_3$) δ 7.35 – 7.23 (m, 2H), 7.25 – 7.17 (m, 1H), 7.17 – 7.06 (m, 3H), 6.69 (s, 1H), 4.22 (q, J = 7.1 Hz, 2H), 3.58 (d, J = 12.5 Hz, 2H), 2.89 (td, J = 12.5, 2.4 Hz, 2H), 2.57 (d, J = 6.7 Hz, 2H), 1.80 – 1.62 (m, 3H), 1.44 – 1.34 (m, 2H), 1.31 (t, J = 7.1 Hz, 3H). ^{13}C NMR (101 MHz, $CDCl_3$) δ 162.7, 152.4, 147.3, 139.6, 129.0, 128.3, 126.1, 115.4, 96.0, 72.1, 61.9, 50.0, 42.7, 37.1, 30.8, 14.3. HRMS (ESI) calculated for $C_{20}H_{23}N_3O_2S$ $[M+Ag]^+$ 476.0556, found 476.0548.

Ethyl (2-cyano-5-morpholinothiophen-3-yl)carbamate (**5e**) was prepared according to the general procedure from 85 mg of thioamide **1e** and 170 mg of α-cyanodiazoacetate **2d**, yield of **5e** 39 mg (28%). Colorless solid, m.p. 163-165 °C. ^{1}H NMR (400 MHz, $CDCl_3$) δ 7.15 (broad, 1H), 6.78 (s, 1H), 4.22 (q, J = 7.1 Hz, 2H), 3.87 – 3.69 (m, 4H), 3.31 – 2.95 (m, 4H), 1.31 (t, J =

7.1 Hz, 3H). ^{13}C NMR (101 MHz, $CDCl_3$) δ 162.6, 152.4, 147.1, 114.9, 96.8, 73.5, 65.8, 62.1, 49.3, 14.3. HRMS (ESI) calculated for $C_{12}H_{15}N_3O_3S$ [M+Ag]$^+$ 387.9880, found 288.9873.

General procedure for the preparation of complexes 6. A mixture of 0.1 mmol of $Rh_2(piv)_4$ (or 0.05 mmol of $Rh_2(oct)_4$) and 2 equiv of the corresponding thioamide **1** in 5 ml of DCM was stirred at room temperature for 24 h, then the solvent was removed in vacuo to give complexes **6** in virtually quantitative yields.

Complex of thioamide 1a with dirhodium(II) tetra(trimethylacetate) (**6a**), was prepared according to the general procedure from 61 mg of $Rh_2(piv)_4$ and 31 mg of thioamide **1a**, yield 91 mg (quant.), green oil. ^{1}H NMR (400 MHz, $CDCl_3$) δ 4.52 (s, 4H), 4.28 (t, *J* = 6.5 Hz, 4H), 4.06 (t, *J* = 6.3 Hz, 4H), 2.36 – 2.05 (m, 8H), 1.00 (s, 36H).

Complex of thioamide 1b with dirhodium(II) tetra(trimethylacetate) (**6b**), was prepared according to the general procedure from 61 mg of $Rh_2(piv)_4$ and 34 mg of thioamide **1b**, yield 94 mg (quant.), green oil. ^{1}H NMR (400 MHz, $CDCl_3$) δ ^{1}H NMR (400 MHz, $CDCl_3$) δ 4.77 – 4.38 (m, 8H), 4.15 – 3.78 (m, 4H), 2.06 – 1.79 (m, 12H), 0.98 (s, 36H).

Complex of thioamide 1c with dirhodium(II) tetra(trimethylacetate) (**6c**) was prepared according to the general procedure from 61 mg of $Rh_2(piv)_4$ and 36 mg of thioamide **1c**, yield 96 mg (quant.), green oil. ^{1}H NMR (400 MHz, $CDCl_3$) δ 4.57 (s, 4H), 4.51 – 4.31 (m, 4H), 4.04 (t, *J* = 6.0 Hz, 4H), 2.24 – 2.07 (m, 4H), 2.05 (d, *J* = 5.1 Hz, 4H), 1.77 (d, *J* = 2.2 Hz, 8H), 0.98 (s, 36H).

Complex of thioamide 1d with dirhodium(II) tetra(trimethylacetate) (**6d**) was prepared according to the general procedure from 61 mg of $Rh_2(piv)_4$ and 52 mg of thioamide **1d**, yield 112 mg (quant.), green oil. ^{1}H NMR (400 MHz, $CDCl_3$) δ 7.44 – 7.10 (m, 10H), 5.87 (d, J = 13.4 Hz, 2H), 4.74 (d, J = 17.9 Hz, 2H), 4.58 – 4.34 (m, 4H), 3.55 – 3.38 (m, 1H), 3.39 – 3.15 (m, 1H), 2.79 – 2.51 (m, 4H), 2.14 – 1.86 (m, 6H), 1.80 – 1.58 (m, 4H), 1.12 – 0.89 (m, 36H).

Complex of thioamide 1e with dirhodium(II) tetra(trimethylacetate) (**6e**), was prepared according to the general procedure from 61 mg of $Rh_2(piv)_4$ and 34 mg of thioamide **1e**. Yield 94 mg (quant.), green oil was crystallized from a mixture of hexane/toluene to afford green solid (90 mg, 96 %), m.p. 213-215 °C (with darkening of the sample at 190 °C on fast heating). ^1H NMR (400 MHz, $CDCl_3$) δ 4.52 (s, 4H), 4.28 (t, J = 6.5 Hz, 4H), 4.06 (t, J = 6.3 Hz, 4H), 2.36 – 2.05 (m, 8H), 1.00 (s, 36H).

Complex of thioamide 1b with dirhodium(II) tetraoctanoate (**6b'**), was prepared according to the general procedure from 39 mg of $Rh_2(oct)_4$ and 17 mg of thioamide **1b**, yield 55 mg (quant.), green oil. ^1H NMR (400 MHz, $CDCl_3$) δ 4.65 – 4.54 (m, 4H), 4.50 (s, 4H), 4.06 – 3.87 (m, 4H), 2.16 (t, J = 7.6 Hz, 8H), 2.05 – 1.76 (m, 12H), 1.53 – 1.36 (m, 8H), 1.30 – 1.01 (m, 32H), 0.85 (t, J = 7.0 Hz, 12H).

Complex of thioamide 1c with dirhodium(II) tetraoctanoate (**6c'**), was prepared according to the general procedure from 39 mg of $Rh_2(oct)_4$ and 18 mg of thioamide **1d**, yield 56 mg (quant.), green oil. ^1H NMR (400 MHz, $CDCl_3$) δ 4.62 – 4.33 (m, 8H), 4.04 (t, J = 6.1 Hz, 4H), 2.27 – 2.07 (m, 12H), 2.07 – 1.94 (m, 4H), 1.91 – 1.61 (m, 8H), 1.56 – 1.34 (m, 8H), 1.33 – 0.99 (m, 32H), 0.85 (t, J = 7.0 Hz, 12H).

Complex of thioamide 1d with dirhodium(II) tetraoctanoate (**6d'**), was prepared according to the general procedure from 39 mg of $Rh_2(oct)_4$ and 26 mg of thioamide **1c**, yield 64 mg (quant.), green oil. ^1H NMR (400 MHz, $CDCl_3$) δ 7.51 – 6.98 (m, 10H), 5.88 (d, J = 13.5 Hz, 2H), 4.61 (d, J = 17.8 Hz, 2H), 4.50 – 4.24 (m, 4H), 3.55 – 3.37 (m, 2H), 3.37 – 3.08 (m, 2H), 2.80 – 2.56 (m, 4H), 2.25 – 2.09 (m, 8H), 2.10 – 1.83 (m, 6H), 1.80 – 1.52 (m, 4H), 1.52 – 1.31 (m, 8H), 1.31 – 1.00 (m, 32H), 0.94 – 0.71 (m, 12H).

Complex of thioamide 1e with dirhodium(II) tetraoctanoate (**6e'**), was prepared according to the general procedure from 39 mg of $Rh_2(oct)_4$ and 17 mg of thioamide **1e**, yield 55 mg (quant.),

green oil. ^{1}H NMR (400 MHz, $CDCl_3$) δ 4.79 – 4.61 (m, 4H), 4.53 (s, 4H), 4.19 – 3.88 (m, 12H), 2.30 – 2.03 (m, 8H), 1.55 – 1.34 (m, 8H), 1.33 – 1.02 (m, 32H), 0.86 (t, J = 7.0 Hz, 12H).

Decomposition of dimethyl diazomalonate 2a with Rh-complex 6e. Diazomalonate **2a** (85 mg, 3 eq) was added in one portion to a solution of Rh-complex **6e** (200 mg, 1 eq) in 5 ml of toluene. No reaction was observed at r. t. for one day, whereas on reflux of the reaction mixture at 110 °C during one hour the initial diazo compound was completely decomposed to produce a mixture of reaction products including thiophenes **3e** (16%) and **4e** (36%) (by ^{1}H NMR).

References

1. Palatinus, L.; Chapuis, G. *J. Appl. Cryst.* **2007**, *40*, 786-790.

2. Palatinus, L.; van der Lee, A. *J. Appl. Cryst.* **2008**, *41*, 975-984.

3. Palatinus, L.; Prathapa, S. J.; van Smaalen, S. *J. Appl. Cryst.* **2012**, *45*, 575-580.

4. Sheldrick, G. M. *Acta Cryst., Section A: Foundations of Crystallography* **2008**, *64*, 112-122.

5. Sheldrick, G. M. *Acta Cryst., Section A: Foundations and Advances* **2015**, *71*, 3-8.

6. Dolomanov, O. V.; Bourhis, L. J.; Gildea, R. J.; Howard, J. A. K.; Puschmann, H. *J. Appl. Cryst.* **2009**, *42*, 339-341.

7. Regitz, M. R.; Maas, G. *Diazo Compounds. Properties and Synthesis*, Academic Press, New York, **1986**, 569.

8. Popik, V. V.; Korneev, S. M.; Nikolaev, V. A.; Korobitsyna, I. K. *Synthesis* **1991**, 195-198.

9. Nikolaev, V. A.; Shevchenko, V. V.; Platz, M. S.; Khimich, N. N. *Russ. J. Org. Chem.* **2006**, *42*, 815-827.

10. Shafran, Yu. M.; Bakulev, V. A.; Mokrushin, V. S.; Alexeev, S. G. *Khim. Geterotsikl. Soedin.* **1984**, *9*, 1266-1270.

11. Filimonov, V. O.; Dianova, L. N.; Galata, K.A.; Beryozkina, T. V.; Novikov, M. S.; Berseneva, V. S.; Eltsov, O. S.; Lebedev, A. T.; Slepukhin, P. A.; Bakulev, V. A. *J. Org. Chem.* **2017**, *82*, 4056-4071.
